# 2-Iodo-*N*-isopropyl-5-methoxybenzamide as a highly reactive and environmentally benign catalyst for alcohol oxidation

**DOI:** 10.3762/bjoc.14.82

**Published:** 2018-04-30

**Authors:** Takayuki Yakura, Tomoya Fujiwara, Akihiro Yamada, Hisanori Nambu

**Affiliations:** 1 Graduate School of Medicine and Pharmaceutical Sciences, University of Toyama, Sugitani, Toyama 930-0194, Japan

**Keywords:** hypervalent iodine, iodobenzamide, organic catalysis, oxidation, oxone

## Abstract

Several *N*-isopropyliodobenzamides were evaluated as catalysts for the oxidation of benzhydrol to benzophenone in the presence of Oxone^®^ (2KHSO_5_·KHSO_4_·K_2_SO_4_) as a co-oxidant at room temperature. A study on the substituent effect of the benzene ring of *N*-isopropyl-2-iodobenzamide on the oxidation revealed that its reactivity increased in the following order of substitution: 5-NO_2_ < 5-CO_2_Me, 3-OMe < 5-OAc < 5-Cl < H, 4-OMe < 5-Me < 5-OMe. The oxidation of various benzylic and aliphatic alcohols using a catalytic amount of the most reactive 5-methoxy derivative successfully resulted in moderate to excellent yields of the corresponding carbonyl compounds. The high reactivity of the 5-methoxy derivative at room temperature is a result of the rapid generation of the pentavalent species from the trivalent species during the reaction. 5-Methoxy-2-iodobenzamide would be an efficient and environmentally benign catalyst for the oxidation of alcohols, especially benzylic alcohols.

## Introduction

**Figure 1:**
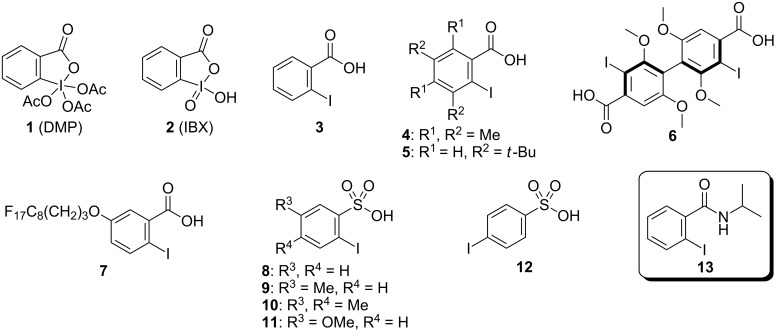
Structures of pentavalent iodine oxidants **1** and **2**, and iodine catalysts **3**–**13**.

The development of an efficient and environmentally benign organic synthesis is required for minimizing material use, energy consumption, and environmental pollution in the production of both bulk and fine chemicals. Oxidation is a fundamental and frequently used transformation in organic synthesis. Heavy metal-based oxidants such as chromium(VI), lead(IV), and mercury(II) have been extensively used for this purpose for a long time. However, these oxidants are highly toxic and produce hazardous waste. Recently, hypervalent iodine oxidants have been widely employed for oxidation in organic synthesis [1–9] because they are nonmetallic, less toxic, and easy to handle, and they allow mild reaction conditions in most cases. Pentavalent iodine reagents such as Dess–Martin periodinane (DMP, **1**) [10] and 2-iodoxybenzoic acid (IBX, **2**) [11] are well known as representative environmentally benign oxidants for alcohol oxidation (Figure 1). However, despite the utility and versatility of these oxidants, they still have several drawbacks: both are potentially explosive, DMP is moisture-sensitive, and IBX is insoluble in common organic solvents. To overcome these drawbacks, IBX analogs [12–22] and several iodoxyarene derivatives [23–38] have been developed, and the stabilization of IBX by combining it with benzoic and isophthalic acids (SIBX) [39] has been reported. Nevertheless, from a green chemistry viewpoint, pentavalent iodine oxidants are not ideal because oxidation reactions require a stoichiometric amount of the oxidant that produces an equimolar amount of organoiodine waste. The catalytic use of pentavalent iodine species is an effective method for reducing the use of iodine compounds and the produced waste [40–44]. 2-Iodobenzoic acid (**3**) [45–46] and its derivatives such as **4**–**7** [20,47–51] and 2-iodobenzenesulfonic acid (**8**) and its derivatives **9**–**11** [52–57] have been developed as catalysts for the oxidation of alcohols in the presence of Oxone^®^ (2KHSO_5_·KHSO_4_·K_2_SO_4_) as a co-oxidant. In these reported systems, high temperatures (40–70 °C) are often required to generate potentially explosive pentavalent iodine compounds in situ except for the reactions involving multisubstituted benzoic acids and benzenesulfonic acid (**4**–**6** and **10**) which can be performed at room temperature [20,48–51,57]. In contrast, the use of a combination of a catalytic amount of RuCl_3_ and a stoichiometric amount of Oxone^®^ as the co-oxidation system does not require heating despite the use of iodobenzene and 4-iodobenzenesulfonic acid (**12**) as catalysts [58–59]. As part of our study on the development of multifunctionalized organocatalysts based on hypervalent iodine chemistry [60–66], we found that *N*-isopropyl-2-iodobenzamide (**13**), when utilized as a catalyst with Oxone^®^ at room temperature, appears to be a promising catalyst for efficient and environmentally benign alcohol oxidation reactions [67]. Herein, we report our efforts on improving the reactivity of 2-iodobenzamide catalysts.

## Results and Discussion

**Figure 2:**
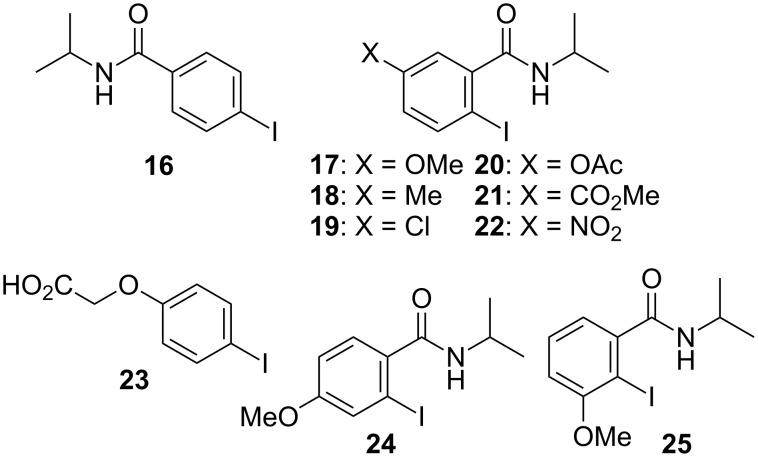
Structures of the catalysts **16**–**25**.

We evaluated several 2-iodobenzoic acid derivatives including esters and amides as catalysts for alcohol oxidation in the presence of Oxone^®^, and we found that the simply modified derivative, *N*-isopropylamide **13**, exhibited excellent catalytic properties at room temperature [67]. Interestingly, the reactivity of **13** was much higher than that of **3** at room temperature but was lower at 70 °C. However, the reactivity of **13** itself was not so high: the oxidation of benzhydrol (**14a**) with 0.3 equiv of **13**, 2.5 equiv of Oxone^®^, and 1 equiv of Bu_4_NHSO_4_ in MeNO_2_/H_2_O (8:3) completed in 12 h at room temperature (25 °C) to produce a 98% yield of benzophenone (**15a,** Table 1, entry 1) [67]. Therefore, enhancement of the reactivity of the catalyst was desired for practical use. To develop a more reactive catalyst, we evaluated several types of *N*-isopropyliodobenzamides (Table 1). Although most of the previously reported iodoarene catalysts based on pentavalent iodine compounds have a carboxyl or sulfoxyl group at the *ortho*-position to the iodine atom, Zhdankin and colleagues reported that both 2-iodobenzenesulfonic acid (**8**) and 4-iodobenzensulfonic acid (**12**) exhibited high reactivity in their RuCl_3_–Oxone^®^ co-oxidant system [58–59]. Therefore, we expected that the reactivity of 4-iodobenzamide **16** (Figure 2) would be similar to that of **13**. However, the oxidation of **14a** with **16** under the above conditions did not complete even after 24 h, and the yield of **15a** and of recovered **14a** was 30% and 67%, respectively (Table 1, entry 2). These results indicate that the reactivity of the iodobenzamides depends on the *ortho*-relationship of the iodine atom to the amide group. Therefore, we then investigated *N*-isopropyl-2-iodobenzamides that have an additional functional group on the benzene ring. Based on the results of our studies on phenol oxidation [60,62] and Ishihara’s [52] and Moorthy’s [20,48] studies on alcohol oxidation, an electron-donating group at the *para*-position to the iodine should be expected to enhance the reactivity of iodoarene catalysts. The investigation began with the 5-substituted 2-iodobenzamides **17**–**22** (Figure 2). Oxidation of **14a** with 5-methoxy-2-iodobenzamide **17** was much faster than that with **13** and was completed within 6 h to produce **15a** in 97% yield (Table 1, entry 3). The 5-methyl derivative **18** exhibited a slightly higher reactivity than **13** (Table 1, entry 4). The introduction of an electron-withdrawing group such as a chloro, acetoxy, methoxycarbonyl, or nitro group at the 5-position led to a decrease in reactivity (Table 1, entries 5–8). These results are in good agreement with the reported studies [20,48,52,60,62]. To confirm the importance of the electron-withdrawing carboxyamide group at the *ortho*-position, we examined the oxidation of **14a** with 4-iodophenoxyacetic acid (**23**); the results showed excellent reactivity for phenol oxidations [60–64]. When **14a** was oxidized with **23**, the reaction was very slow and yielded only 23% of **15a** and 75% of recovered **14a** even after 48 h (Table 1, entry 9) [68]. Since the 5-methoxy derivative **17** was the most reactive, we then investigated the reactivities of **24** and **25**, which have methoxy groups at the *meta*- and *ortho*-positions to the iodine atom, and found that 4-methoxyamide **24** exhibited the same reactivity as nonsubstituted **13** (Table 1, entry 10). On the other hand, oxidation with 3-methoxyamide **25** was much slower than that with **13** and needed 23 h to complete (Table 1, entry 11). Although the *ortho*- and the *para*-methoxy groups should show a similar electronic effect on the iodine atom, the lower reactivity of **25** might be explained by the steric hindrance around the iodine atom of **25**. Consequently, the reactivities of the 2-iodobenzamides **17**–**22**, **24**, and **25** decreased in the following order of substitution: 5-OMe (**17**) > 5-Me (**18**) > H (**13**), 4-OMe (**24**) > 5-Cl (**19**) > 5-OAc (**20**) > 5-CO_2_Me (**21**), 3-OMe (**25**) > 5-NO_2_ (**22**).

**Table 1:** Oxidation of benzhydrol (**14a**) to benzophenone (**15a**) catalyzed by **13** and **16**–**25**.

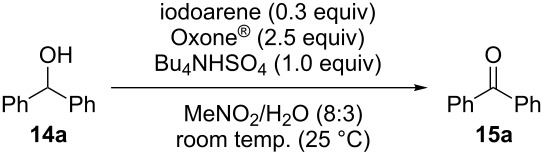				

entry^a^	iodobenzamide	time (h)	yield (%)^b^	recovery of **14a** (%)^b^

1^c^	**13**	12	98	–
2	**16**	24	30	67
3	**17**	6	97	–
4	**18**	10	98	–
5	**19**	18	98	–
6	**20**	19	98	–
7	**21**	23	96	–
8	**22**	46	98	–
9	**23**	48	23	75
10	**24**	13	98	–
11	**25**	23	99	–

^a^All reactions were performed on a 0.5 mmol scale. ^b^Isolated yield. ^c^Ref. [67]

With a highly reactive catalyst in hand, we examined the oxidation of various secondary alcohols **14b**–**f** and primary alcohols **14g**–**k** with 0.3 equiv of **17** in the presence of 2.5 equiv of Oxone^®^ and 1 equiv of Bu_4_NHSO_4_ in an 8:3 mixture of MeNO_2_ and water at room temperature. These results as well as those obtained from a similar oxidation using **13** as a catalyst are summarized in Table 2. The secondary benzylic alcohols **14b**–**e** were oxidized with **17** in much shorter reaction times than those oxidized with **13** to give the corresponding ketones **15b**–**e** in good to excellent yields (Table 2, entries 1–4). Oxidation of the aliphatic secondary alcohol **14f** with **17** required a slightly longer reaction time than that with **13** (Table 2, entry 5). The primary alcohols **14g**–**k** were converted into the corresponding carboxylic acids **26g**–**k** in moderate to excellent yields (Table 2, entries 6–10). However, the reaction times of the oxidations of **14h**, **14i,** and **14k** with **17** were similar to those involving **13**. These results may be due to the slow oxidation of the aldehydes to carboxylic acids [69]. The catalyst **17** was stable under the oxidation conditions and it was recovered in 67–92% after reductive treatment.

**Table 2:** Oxidation of various alcohols **14b**–**k** with **17**.^a^

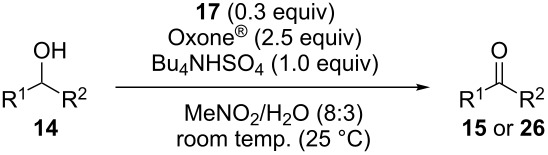				

entry	alcohol	carbonyl compound	time (h)^b^	yield (%)^b,c^

1	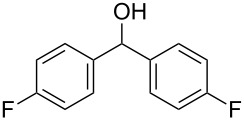 **14b**	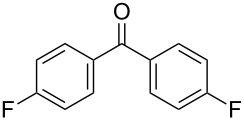 **15b**	9 (15)	95 (74)
2	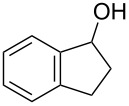 **14c**	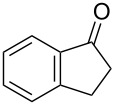 **15c**	7 (17)	86 (71)
3	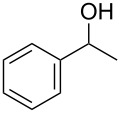 **14d**	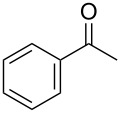 **15d**	11 (14)	62 (70)
4	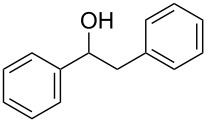 **14e**	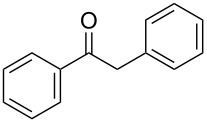 **15e**	10 (20)	98 (97)
5	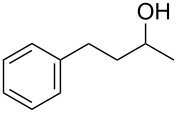 **14f**	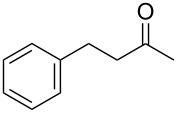 **15f**	36 (30)	64 (74)
6	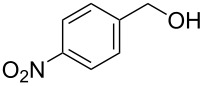 **14g**	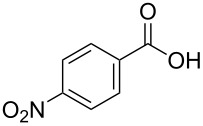 **26g**	10 (20)	89 (82)
7	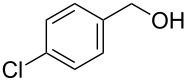 **14h**	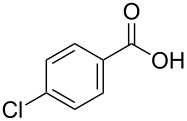 **26h**	24 (24)	83 (74)
8	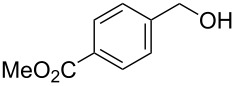 **14i**	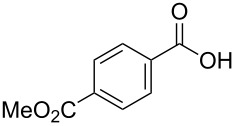 **26i**	16 (16)	96 (89)
9	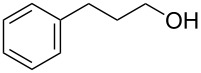 **14j**	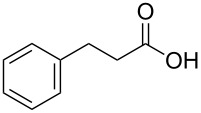 **26j**	9 (20)	85 (90)
10	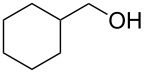 **14k**	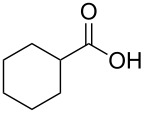 **26k**	36 (36)	59 (58)

^a^All reactions were performed on a 0.5 mmol scale. ^b^The results obtained for the oxidation using **13** are shown in parentheses [67]. ^c^Isolated yield.

**Scheme 1:**
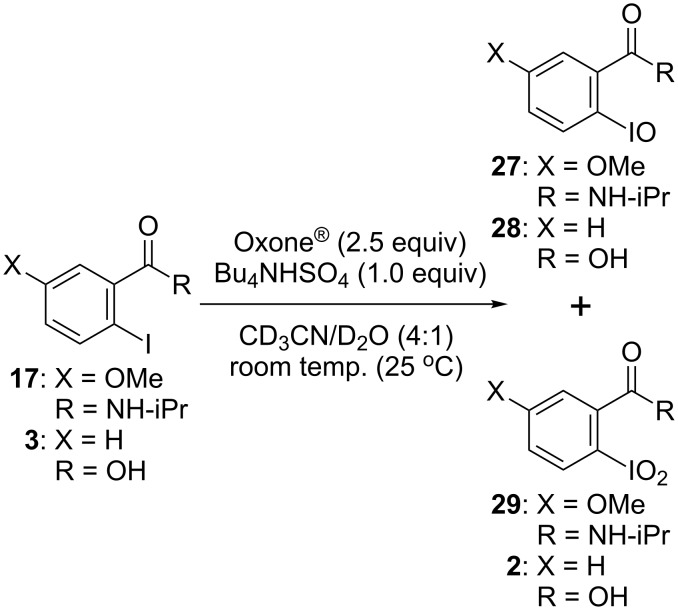
Oxidation of the monovalent iodine derivatives **17** and **3** to the pentavalent iodine derivatives **29** and **2** using Oxone^®^.

**Figure 3:**
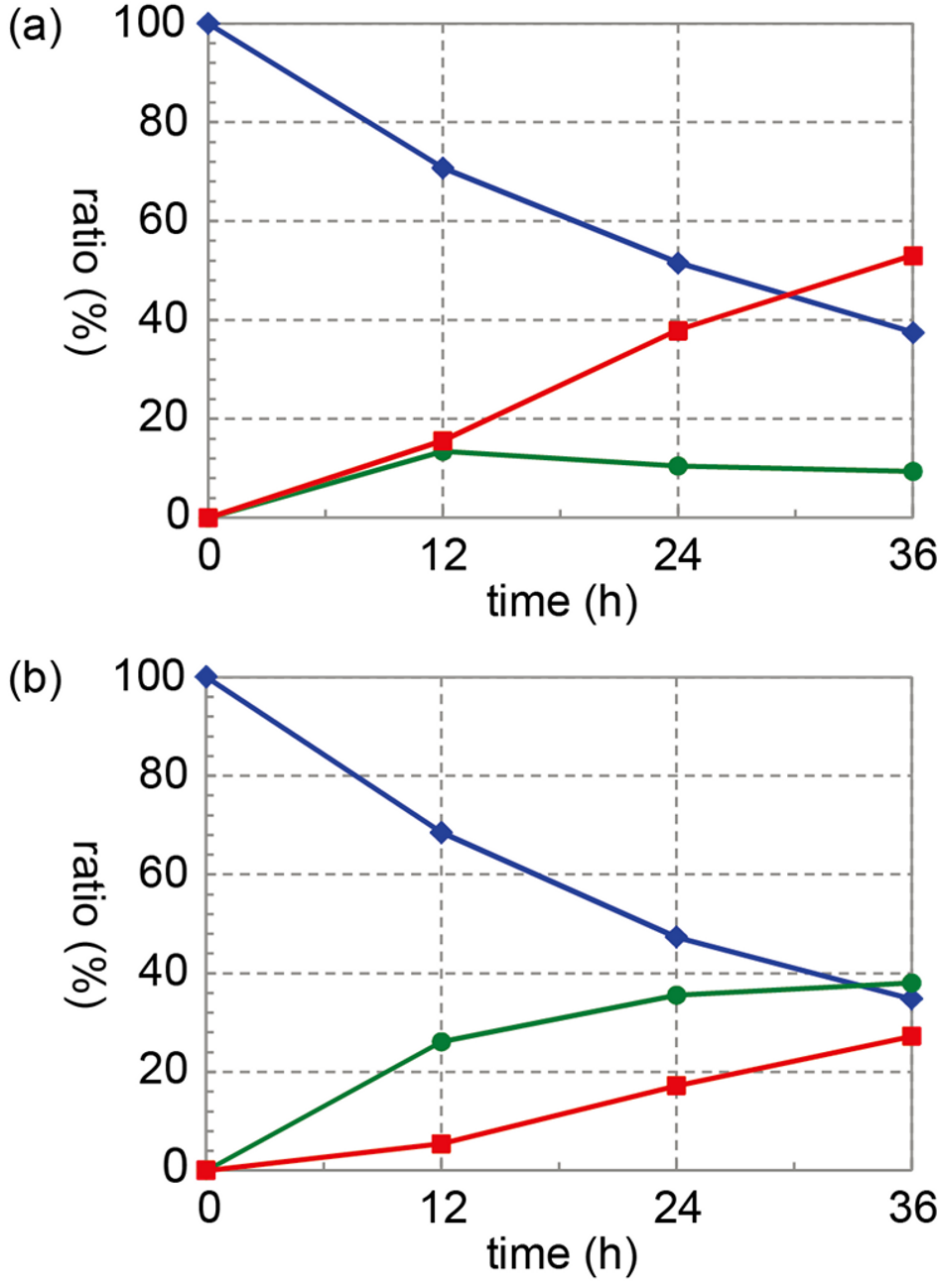
Reaction profile of the oxidation of (a) iodobenzamide **17** and (b) 2-iodobenzoic acid (**3**) with Oxone^®^ in the presence of Bu_4_NHSO_4_ in a 4:1 mixture of CD_3_CN and D_2_O: monovalent iodine derivatives **17** and **3** (blue), trivalent iodine derivatives **27** and **28** (green), and pentavalent iodine derivatives **29** and **2** (red).

The next objective was to investigate the oxidation mechanism of 2-iodobenzamide catalysts. Zhdankin and colleagues reported the oxidation of alcohols to their corresponding carbonyl compounds using several 2-iodoxybenzamides [23]. Therefore, we tried to confirm the formation of 2-iodoxybenzamide **29** from 2-iodobenzamide **17** in an oxidation reaction (Scheme 1). Iodoarene **17** was treated with 2.5 equiv of Oxone^®^ and 1 equiv of Bu_4_NHSO_4_ in a 4:1 mixture of acetonitrile-*d*_3_ and D_2_O at a low concentration (0.01 M) at room temperature, and the reaction was monitored by ^1^H NMR spectroscopy. The reaction profiles for **17** as well as for 2-iodobenzoic acid (**3**) are summarized in Figure 3. In the reaction involving **17**, the amount of pentavalent iodine derivative **29** gradually increased with a decrease in the amount of **17** and only a small amount of trivalent iodine derivative **27** was observed during the reaction. The ratio of **17** to trivalent **27** and pentavalent **29** was determined to be 38:9:53 after 36 h. In the oxidation of **3**, monovalent **3** was consumed at almost the same rate as the oxidation of **17**. However, the formation of pentavalent **2** was much slower than that of **29**, and a considerable amount of trivalent **28** remained during the reaction. After 36 h, the oxidation of **3** generated 27% of **2** with 35% unreacted **3** and 38% trivalent **28**. Thus, it is clear that the pentavalent iodine species **2** and **29** were generated during the oxidation reactions of monovalent **3** and **17** and that the oxidation of trivalent **27** to pentavalent **29** is much faster than that of **28** to **2** at room temperature. These results also suggest that the formation of the pentavalent iodine species from the corresponding trivalent iodine species might be the rate-determining step in the catalytic hypervalent iodine oxidation of **17** [70].

**Scheme 2:**
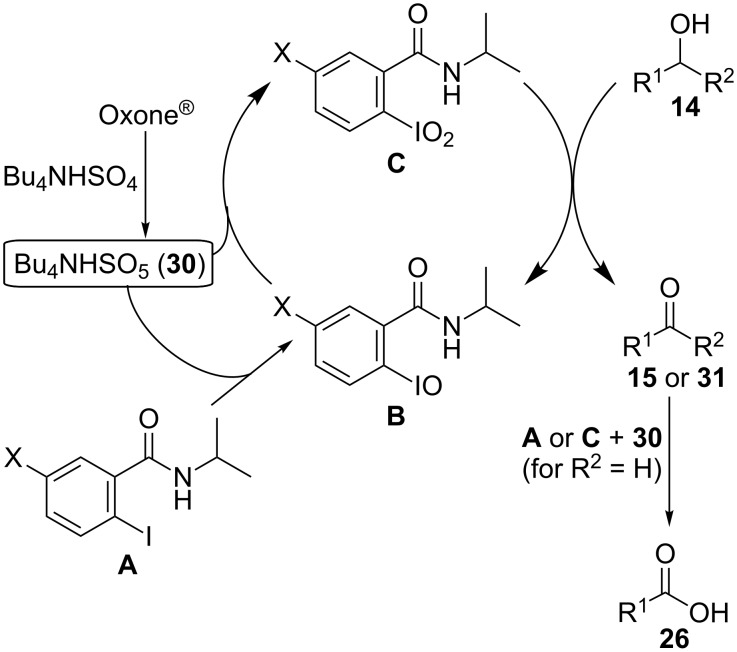
Plausible reaction mechanism for the oxidation of alcohols catalyzed by the 2-iodobenzamides.

On the basis of the above results, a plausible mechanism for the oxidation catalyzed by the 2-iodobenzamides is shown in Scheme 2. Iodobenzamide **A** is readily oxidized by tetra-*n*-butylammonium peroxymonosulfate (Bu_4_NHSO_5_, **30**), which is derived from Bu_4_NHSO_4_ and Oxone^®^, to pentavalent iodine species **C** at room temperature. The resultant **C** oxidizes alcohol **14** to ketone **15** or aldehyde **31** during its reduction to trivalent iodine **B**. Aldehyde **31** is further oxidized with **30** with the assistance of **A** or **C** [67] to give carboxylic acid **26**. Iodine **B** is re-oxidized with **30** to regenerate **C**. The oxidation proceeds at room temperature because of the fast oxidation of trivalent **B** to pentavalent **C**.

## Conclusion

In summary, we have developed 2-iodo-*N*-isopropyl-5-methoxybenzamide (**17**) as an efficient catalyst for the oxidation of primary and secondary alcohols. The reaction of benzylic and aliphatic alcohols **14** with a catalytic amount of **17** in the presence of Oxone^®^ and Bu_4_NHSO_4_ at room temperature proceeded smoothly to provide good to excellent yields of the corresponding carbonyl compounds **15** and **26**. The higher reactivity of **17** at room temperature results from the rapid oxidation of trivalent iodine compound **27** to the pentavalent compound **29**. 5-Methoxy-2-iodobenzamide **17** promises to be an efficient and environmentally benign catalyst for oxidation of alcohol, especially benzylic alcohols.

## Experimental

**Typical experimental procedure for the oxidation of secondary alcohols 14a**–**f:** Secondary alcohol **14** (0.50 mmol) was added to a solution of the catalyst (0.15 mmol) and Bu_4_NHSO_4_ (170 mg, 0.50 mmol) in a mixture of MeNO_2_ (1.6 mL) and water (0.6 mL), followed by Oxone^®^ (768 mg, 1.25 mmol) at room temperature (25 °C). After **14** was completely consumed, as indicated by TLC, the resulting mixture was diluted using EtOAc and was washed with water. The organic layer was then washed with saturated aqueous Na_2_S_2_O_3_ and saturated aqueous NaHCO_3_, dried over MgSO_4_, filtered, and concentrated under reduced pressure. The residue was purified by silica gel column chromatography to give pure ketone **15** and the catalyst.

**Typical experimental procedure for the oxidation of primary alcohols 14g**–**k:** Primary alcohol **14** (0.50 mmol) was added to a solution of the catalyst (0.15 mmol) and Bu_4_NHSO_4_ (170 mg, 0.50 mmol) in a mixture of MeNO_2_ (1.6 mL) and water (0.6 mL), followed by Oxone^®^ (768 mg, 1.25 mmol) at room temperature (25 °C). After **14** was completely consumed, as indicated by TLC, the resulting mixture was diluted with EtOAc, water, and saturated aqueous Na_2_S_2_O_3_. The organic layer was then washed with saturated aqueous Na_2_S_2_O_3_, saturated aqueous NaHCO_3_, and brine, dried over MgSO_4_, filtered, and concentrated under reduced pressure. The residue was purified by silica gel column chromatography to give the catalyst. The combined aqueous layers were acidified with 10% HCl and extracted with EtOAc. The organic layer was washed with brine, dried over MgSO_4_, filtered, and concentrated under reduced pressure. The residue was purified by silica gel column chromatography to give pure carboxylic acid **26**.

## Supporting Information

File 1 Experimental details and the ^1^H and ^13^C NMR spectra of the catalysts, the substrates, and the products.
