# Supplementary material for: 2-Iodo-N-isopropyl-5-methoxybenzamide as a highly reactive and environmentally benign catalyst for alcohol oxidation
Source: Beilstein J Org Chem. 2018 Apr 30;14:971–8. doi: 10.3762/bjoc.14.82 (PMC6009128; doi:10.3762/bjoc.14.82)
Supplement: File 1 — Experimental details and the 1H and 13C NMR spectra of the catalysts, the substrates, and the products. [file Beilstein_J_Org_Chem-14-971-s001.pdf]

Supporting Information

for

2-Iodo-*N*-isopropyl-5-methoxybenzamide as a highly reactive and environmentally benign catalyst for alcohol oxidation

Takayuki Yakura*, Tomoya Fujiwara, Akihiro Yamada, and Hisanori Nambu

Address: Graduate School of Medicine and Pharmaceutical Sciences, University of Toyama, Sugitani, Toyama 930-0194, Japan

Email: Takayuki Yakura* - yakura@pha.u-toyama.ac.jp

*Corresponding author

Experimental details and the ^{1}H and ^{13}C NMR spectra of the catalysts, the substrates, and the products

Contents

**1. General experimental**	**S1**
**2. Experimental details and analytical data**	**S2–S12**
**3. ^{1}H and ^{13}C NMR Spectra**	**S13-S37**
**4. References**	**S38**

1. General experimental

Melting points were determined using a Yanaco micro melting point apparatus and are uncorrected. Infrared (IR) spectra were recorded using a JEOL FT/IR-460Plus spectrometer. All NMR spectra were recorded using JEOL ECX-400P or JEOL ECA-500II spectrometers. Proton (^{1}H) NMR spectra were recorded at 400 or 500 MHz. Carbon-13 (^{13}C) NMR spectra were recorded using the broadband proton decoupling at 100 or 126 MHz. All chemical shifts, δ, are stated in units of parts per million (ppm), relative to a standard. For ^{1}H NMR, the reference point is tetramethylsilane (= 0.00 ppm), CD_3CN (= 1.93 ppm, solvent residual signal), or DMSO-d_6 (= 2.49 ppm, solvent residual signal). For ^{13}C NMR, the reference point is $CDCl_3$ (= 77.0 ppm), CD_3CN (= 1.3 ppm), or DMSO-d_6 (= 39.7 ppm). Electron ionization (EI) mass spectra were recorded using a JEOL JMS-GCmate II spectrometer. Values are reported as a ratio of mass to charge (*m*/*z*). Column chromatography was performed on Nacalai Tesque Silica Gel 60 PF_{254} (0.005–0.050 mm), Kanto chemical silica gel 60N (0.040–0.050 mm) or Merck 9385 silica gel 60 (0.040–0.063 mm). Thin layer chromatography was performed on Merck 5715 silica gel 60 F_{254} or Merck 5554 silica gel 60 F_{254}.

2. Experimental details and analytical data

Preparation of catalyst

4-Iodo-*N*-isopropylbenzamide (**16**)

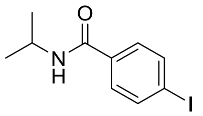

16

A solution of 4-iodobenzoic acid (248 mg, 1.0 mmol) in thionyl chloride (1.25 mL) was heated at reflux with stirring for 2 h. The resulting solution was concentrated under reduced pressure. The remaining thionyl chloride was removed by azeotropic distillation with benzene. The residue was dissolved in anhydrous CH_2Cl_2 (3.3 mL). To the mixture were added isopropylamine (71 mg, 1.2 mmol) and triethylamine (304 mg, 3.0 mmol) at 0 ºC under a nitrogen atmosphere. After stirring at room temperature for 17 h, the resulting solution was diluted with EtOAc. The mixture was washed with 10% HCl, saturated aqueous $NaHCO_3$, water, and brine; dried over Na_2SO_4; filtered; and concentrated under reduced pressure. The residue was purified by recrystallization from hexane and $CHCl_3$ to give **16** (105 mg, 36% in 2 steps) as colorless needles: mp 175–176 °C (hexane/$CHCl_3$); ^{1}H NMR (400 MHz, $CDCl_3$) δ 7.79–7.76 (2H, m), 7.49–7.46 (2H, m), 5.86 (1H, br s), 4.33–4.21 (1H, m), 1.26 (6H, d, J = 6.9 Hz). The data were in accordance with the literature values [1].

2-Iodo-*N*-isopropyl-5-methoxybenzamide (**17**)

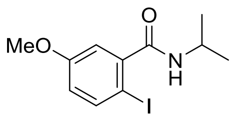

17

A solution of sodium nitrite (1.56 g, 22.5 mmol) in water (4.5 mL) was added dropwise to a solution of 2-amino-5-hydroxybenzoic acid (2.30 g, 15.0 mmol) in a mixture of water (20 mL) and concentrated sulfuric acid (2.8 mL) at 0 ºC. After stirring for 5 min, a solution of potassium iodide (3.74 g, 22.5 mmol) in water (4.5 mL) was added dropwise to the mixture. The resulting solution was heated at 100 ºC with stirring for 1 h and then allowed to cool to room temperature. After stirring for 14 h, the mixture was cooled to 0 ºC. The precipitate was collected by filtration, washed with cold water, and dried in vacuo to give crude 5-hydroxy-2-iodobenzoic acid (3.26 g) as a brown solid, which was dissolved in anhydrous DMF (65 mL). To this solution were added potassium carbonate (12.8 g, 92.7 mmol) and iodomethane (5.8 mL, 92.7 mmol) at room temperature under a nitrogen atmosphere. After stirring for 12 h, the mixture was filtered through a pad of celite and the pad was washed with Et_2O. The filtrate was acidified with 10% HCl and then extracted with Et_2O. The organic layer was washed with saturated aqueous $NaHCO_3$, water, and brine, dried over Na_2SO_4; filtered; and concentrated under reduced pressure. The residue was purified by silica gel column chromatography (eluent: hexane/EtOAc = 4/1) to give methyl 2-iodo-5-methoxybenzoate [2] (3.44 g, 79% in 2 steps) as a pale yellow oil: ^{1}H NMR (500 MHz, $CDCl_3$) δ 7.83 (1H, d, J = 8.6 Hz), 7.35 (1H, d, J = 2.9 Hz), 6.75 (1H, dd, J = 8.6, 2.9 Hz), 3.93 (3H, s), 3.82 (3H, s). To a solution of methyl 2-iodo-5-methoxybenzoate (1.21 g, 4.13 mmol) in a 3:1 mixture of MeOH and water (47 mL) was added lithium hydroxide hydrate (260 mg, 6.2 mmol) at room temperature. After stirring for 15 h, the resulting mixture was diluted with saturated aqueous $NaHCO_3$ and washed with CH_2Cl_2. The aqueous layer was acidified with 10% HCl and then extraceted with CH_2Cl_2. The last organic layer was washed with brine, dried over Na_2SO_4, filtered, and concentrated under reduced pressure. The residue was dissolved in thionyl chloride (5.1 mL). After heating at reflux with stirring for 2 h, the resulting solution was concentrated under reduced pressure. The remaining thionyl chloride was removed by azeotropic distillation with benzene. The residue was dissolved in anhydrous CH_2Cl_2 (13.5 mL). To the mixture were added isopropylamine (290 mg, 4.9 mmol) and triethylamine (1.24 g, 12.2 mmol) at 0 °C under a nitrogen atmosphere. After stirring at room temperature for 6 h, the resulting solution was diluted with EtOAc. The mixture was washed with 10% HCl, saturated aqueous $NaHCO_3$, water, and brine; dried over Na_2SO_4; filtered; and concentrated under reduced

pressure. The residue was purified by recrystallization from hexane and $CHCl_3$ to give **17** (991 mg, 75% in 3 steps) as colorless needles: mp 147–148.5 °C (hexane/$CHCl_3$); IR (KBr) *ν* 3278, 2972, 1640, 1584, 1542, 1468, 1391, 1351, 1312, 1278, 1262, 1236, 1146, 1114, 1041, 1012, 927, 867, 816, 798, 714 cm^{-1}; ^{1}H NMR (500 MHz, $CDCl_3$) δ 7.68 (1H, d, *J* = 8.6 Hz), 6.96 (1H, d, *J* = 2.9 Hz), 6.68 (1H, dd, *J* = 8.6, 2.9 Hz), 5.61 (1H, br d, *J* = 6.3 Hz), 4.34–4.24 (1H, m), 3.80 (3H, s), 1.29 (6H, d, *J* = 6.3 Hz); ^{13}C NMR (126 MHz, $CDCl_3$) δ 168.2, 159.7, 143.2, 140.4, 117.6, 114.0, 80.6, 55.5, 42.2, 22.6; HRMS (EI) calcd for $C_{11}H_{14}INO_2$ ($[M]^+$): 319.0070; found 319.0060.

2-Iodo-*N*-isopropyl-5-methylbenzamide (**18**)

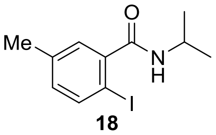

18

A solution of sodium nitrite (513 mg, 7.44 mmol) in water (3.7 mL) was added dropwise to a solution of 2-amino-5-methylbenzoic acid (562 mg, 3.72 mmol) in a mixture of water (9.3 mL), acetone (3.1 mL), and concentrated HCl (1.8 mL) at 0 °C. After stirring for 2 h, potassium iodide (1.24 g, 7.44 mmol) was added to the mixture. The resulting solution was stirred at 0 ºC for 0.5 h, heated at 90 ºC for 10 min, and then allowed to cool to room temperature. The mixture was extracted with $CHCl_3$. The organic layer was washed with saturated aqueous $Na_2S_2O_3$ and water, dried over Na_2SO_4, filtered, and concentrated under reduced pressure. The residue was purified by silica gel column chromatography (eluent: hexane/EtOAc = 4/1) to give 2-iodo-5-methylbenzoic acid [3] (829 mg, 85%) as a colorless solid: ^{1}H NMR (400 MHz, $CDCl_3$) δ 7.91 (1H, d, *J* = 8.2 Hz), 7.84 (1H, d, *J* = 1.8 Hz), 7.02 (1H, dd, *J* = 8.2, 1.8 Hz), 2.36 (3H, s). To a solution of 2-iodo-5-methylbenzoic acid (252 mg, 0.96 mmol) was added thionyl chloride (1.2 mL). After heating at reflux with stirring for 2 h, the resulting solution was concentrated under reduced pressure. The remaining thionyl chloride was removed by azeotropic distillation with benzene. The residue was dissolved in anhydrous CH_2Cl_2 (3 mL). To the mixture were added isopropylamine (68 mg, 1.15 mmol) and triethylamine (291 mg, 2.88 mmol) at 0 °C under a nitrogen atmosphere. After stirring at room temperature for 6 h, the resulting solution was diluted with EtOAc. The mixture was washed with 10% HCl, saturated aqueous $NaHCO_3$, water, and brine; dried over Na_2SO_4; filtered; and concentrated under reduced pressure. The residue was purified by recrystallization from hexane and $CHCl_3$ to give **18** (203 mg, 70% in 2 steps) as colorless needles: mp 147–149 °C (hexane/$CHCl_3$); IR (KBr) *ν* 3278, 2970, 1640, 1591, 1568, 1540, 1465, 1455, 1363, 1346, 1302, 1266, 1221, 1170, 1159, 1147, 1130, 1017, 922, 885, 856, 810, 797, 778, 721, 692, 666 cm^{-1}; ^{1}H NMR (400 MHz, $CDCl_3$) δ 7.69 (1H, d, *J* = 8.2 Hz), 7.21 (1H, d, *J* = 2.3 Hz), 6.92–6.89 (1H, m), 5.54 (1H, br s), 4.35–4.23 (1H, m), 2.31 (3H, s), 1.29 (6H, d, *J* = 6.4 Hz); ^{13}C NMR (100 MHz, $CDCl_3$) δ 168.6, 142.2, 139.4, 138.3, 131.9, 129.1, 88.2, 42.1, 22.6, 20.8; HRMS (EI) calcd for $C_{11}H_{14}INO$ ($[M]^+$): 303.0120; found 303.0093.

5-Chloro-2-iodo-*N*-isopropylbenzamide (**19**)

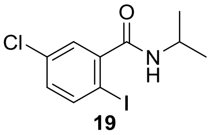

19

According to the procedure reported by Yu and co-workers [3], palladium acetate (22 mg, 0.10 mmol), iodobenzene diacetate (644 mg, 2.0 mmol), iodine (507 mg, 2.0 mmol), and tetrabutylammonium iodine (739 mmol, 2.0 mmol) were added to a solution of 3-chlorobenzoic acid (313 mg, 2.0 mmol) in 1,2-dichloroethane (20 mL). The mixture was heated at 100 ºC with stirring for 2 h and then allowed to cool to room temperature. Iodobenzene diacetate (644 mg, 2.0 mmol) and iodine (507 mg, 2.0 mmol) were added to the mixture. After stirring at 100 °C for 4 h, the resulting mixture was diluted with 10% sodium carbonate. The aqueous layer was separated, washed with Et_2O, and then acidified with 10% HCl. The resulting mixture was extracted with EtOAc and the organic layer was dried over Na_2SO_4; filtered; and concentrated

under reduced pressure. The residue was purified by silica gel column chromatography (eluent: hexane/EtOAc = 4/1) to give crude 5-chloro-2-iodobenzoic acid (223 mg) as a colorless solid, which was suspended in thionyl chloride (2 mL). After stirring under reflux conditions for 2 h, the resulting mixture was concentrated under reduced pressure. The remaining thionyl chloride was removed by azeotropic distillation with benzene. The residue was dissolved in anhydrous CH_2Cl_2 (3 mL). To the mixture were added isopropylamine (56 mg, 0.95 mmol) and triethylamine (240 mg, 2.37 mmol) at 0 °C under a nitrogen atmosphere. After stirring at room temperature for 13 h, the resulting solution was diluted with EtOAc. The mixture was washed with 10% HCl, saturated aqueous $NaHCO_3$, water, and brine; dried over Na_2SO_4; filtered; and concentrated under reduced pressure. The residue was purified by recrystallization from hexane and $CHCl_3$ to give **19** (138 mg, 21% in 3 steps) as colorless needles: mp 160.5–162 °C (hexane/$CHCl_3$); IR (KBr) ν 3259, 2972, 1641, 1577, 1546, 1452, 1378, 1343, 1304, 1261, 1155, 1096, 1020, 906, 884, 814, 725 cm^{-1}; ^{1}H NMR (400 MHz, $CDCl_3$) δ 7.75 (1H, d, J = 8.7 Hz), 7.37 (1H, d, J = 2.8 Hz), 7.08 (1H, dd, J = 8.7, 2.8 Hz), 5.54 (1H, br s), 4.34–4.23 (1H, m), 1.29 (6H, d, J = 6.4 Hz); ^{13}C NMR (100 MHz, $CDCl_3$) δ 167.2, 143.8, 140.8, 134.7, 131.1, 128.3, 89.5, 42.4, 22.5; HRMS (EI) calcd for $C_{10}H_{11}ClINO$ ($[M]^+$): 322.9574; found 322.9580.

5-Acetoxy-2-iodo-*N*-isopropylbenzamide (**20**)

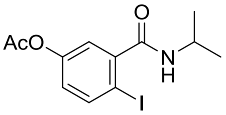

20

To a solution of 5-hydroxy-2-iodobenzoic acid (264 mg, 1.0 mmol) in pyridine (0.57 mL) was added acetic anhydride (410 mg, 4.0 mmol) at room temperature under a nitrogen atmosphere. After stirring for 1 h, the resulting mixture was acidified with 10% HCl and then extracted with EtOAc. The organic layer was washed with 10% HCl and brine; dried over Na_2SO_4; filtered; and concentrated under reduced pressure. The residue was purified by silica gel column chromatography (eluent: hexane/EtOAc = 1/1) to give 5-acetoxy-2-iodobenzoic acid [3] (296 mg, 97%) as a colorless crystalline solid: ^{1}H NMR (400 MHz, $CDCl_3$) δ 8.05 (1H, d, J = 8.7 Hz), 7.79 (1H, d, J = 2.8 Hz), 7.02 (1H, dd, J = 8.7, 2.8 Hz), 2.33 (3H, s). To a solution of 5-acetoxy-2-iodobenzoic acid (296 mg, 0.97 mmol) was added thionyl chloride (2 mL). After heating at reflux with stirring for 3 h, the resulting solution was concentrated under reduced pressure. The remaining thionyl chloride was removed by azeotropic distillation with benzene. The residue was dissolved in anhydrous CH_2Cl_2 (3 mL). To the mixture were added isopropylamine (69 mg, 1.16 mmol) and triethylamine (294 mg, 2.91 mmol) at 0 ºC under a nitrogen atmosphere. After stirring at room temperature for 14 h, the resulting solution was diluted with EtOAc. The mixture was washed with 10% HCl, saturated aqueous $NaHCO_3$, water, and brine; dried over Na_2SO_4; filtered; and concentrated under reduced pressure. The residue was purified by recrystallization from hexane and $CHCl_3$ to give **20** (138 mg, 41% in 2 steps) as colorless needles: mp 143–144.5 °C (hexane/$CHCl_3$); IR (KBr) ν 3240, 3064, 2970, 1756, 1638, 1556, 1459, 1366, 1330, 1216, 1203, 1175, 1012, 947, 914, 822, 724 cm^{-1}; ^{1}H NMR (400 MHz, $CDCl_3$) δ 7.82 (1H, d, J = 8.7 Hz), 7.13 (1H, d, J = 2.8 Hz), 6.86 (1H, dd, J = 8.7, 2.8 Hz), 5.71 (1H, br d, J = 7.3 Hz), 4.32–4.20 (1H, m), 2.29 (3H, s), 1.27 (6H, d, J = 6.4 Hz) ^{13}C NMR (100 MHz, $CDCl_3$) δ 168.9, 167.4, 150.7, 143.5, 140.6, 124.5, 121.7, 88.1, 42.2, 22.5, 21.0; HRMS (EI) calcd for $C_{12}H_{14}INO_3$ ($[M]^+$): 347.0019; found 347.0027.

2-Iodo-*N*-isopropyl-5-methoxycarbonylbenzamide (**21**)

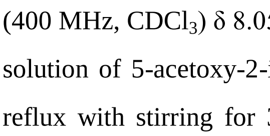

21

A solution of sodium nitrite (75 mg, 1.08 mmol) in water (0.54 mL) was added dropwise to a solution of 2-amino-5-methoxycarbonylbenzoic acid [4] (106 mg, 0.55 mmol) in a 7:3:1 mixture of water (1.9 mL), acetone (0.8 mL), and concentrated HCl (0.26 mL). After stirring

for 2 h, potassium iodide (179 mg, 1.08 mmol) was added to the mixture. The resulting mixture was stirred at 0 ºC for 0.5 h and then allowed to warm to room temperature. The mixture was extracted with $CHCl_3$. The organic layer was washed with water, dried over Na_2SO_4, filtered, and concentrated under reduced pressure. The residue was purified by silica gel column chromatography (eluent: hexane/EtOAc = 4/1) to give methyl 2-iodo-5-methoxycarbonylbenzoic acid [3] (130 mg, 77%) as a colorless solid: ^{1}H NMR (400 MHz, $CDCl_3$) δ 8.64 (1H, d, *J* = 1.8 Hz), 8.17 (1H, d, *J* = 8.2 Hz), 7.83 (1H, dd, *J* = 8.2, 1.8 Hz), 3.96 (3H, s). A solution of 2-iodo-5-methoxycarbonylbenzoic acid (118 mg, 0.39 mmol) in thionyl chloride (1.5 mL) was heated at reflux with stirring for 2 h. The resulting solution was concentrated under reduced pressure. The remaining thionyl chloride was removed by azeotropic distillation with benzene. The residue was dissolved in anhydrous CH_2Cl_2 (1.3 mL). To the mixture were added isopropylamine (28 mg, 0.47 mmol) and triethylamine (121 mg, 1.2 mmol) at 0 °C under a nitrogen atmosphere. After stirring at room temperature for 1 h, the resulting solution was diluted with EtOAc. The mixture was washed with 10% HCl, saturated aqueous $NaHCO_3$, water, and brine; dried over Na_2SO_4; filtered; and concentrated under reduced pressure. The residue was purified by recrystallization from hexane and $CHCl_3$ to give **21** (65 mg, 48% in 2 steps) as colorless prisms: mp 170–171.5 °C (hexane/$CHCl_3$); IR (KBr) *ν* 3271, 3063, 2978, 2958, 2936, 1732, 1641, 1589, 1542, 1454, 1435, 1391, 1367, 1350, 1281, 1245, 1196, 1154, 1128, 1109, 1016, 984, 918, 905, 853, 840, 815, 757, 725, 689, 656 cm^{-1}; ^{1}H NMR (400 MHz, $CDCl_3$) δ 7.99 (1H, d, *J* = 2.3 Hz), 7.95 (1H, d, *J* = 8.2 Hz), 7.70 (1H, dd, *J* = 8.2, 2.3 Hz), 5.63 (1H, br d, *J* = 6.9 Hz), 4.36–4.24 (1H, m), 3.92 (3H, s), 1.30 (6H, d, *J* = 6.4 Hz); ^{13}C NMR (100 MHz, $CDCl_3$) δ 167.7, 165.9, 142.8, 140.1, 131.3, 130.2, 128.7, 98.8, 52.5, 42.3, 22.6; HRMS (EI) calcd for $C_{12}H_{14}INO_3$ ($[M]^+$): 347.0019; found 347.0015.

2-Iodo-*N*-isopropyl-5-nitrobenzamide (**22**)

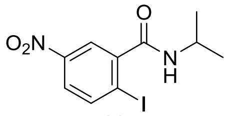

22

2-Amino-5-nitrobenzoic acid (500 mg, 2.75 mmol) was dissolved in 0.5 M NaOH (5 mL) at 70 ºC. The resulting solution was allowed to cool to 0 °C and the treated with concentrated HCl (1 mL). To the mixture was added dropwise a solution of sodium nitrite (196 mg, 2.84 mmol) in water (2.5 mL). After stirring for 0.5 h, a solution of potassium iodide (913 mg, 5.5 mmol) in water (2.5 mL) was added dropwise to the mixture. The resulting solution was stirred for 1 h and allowed to warm to room temperature. After stirring for 12 h, the precipitate was collected by filtration, washed with water, and dried *in vacuo*. The residue was purified by silica gel column chromatography (eluent: hexane/EtOAc = 1/1) to give 5-nitro-2-iodobenzoic acid [5] (453 mg, 56%) as a pale yellow solid: ^{1}H NMR (400 MHz, $CDCl_3$) δ 8.80 (1H, d, *J* = 2.8 Hz), 8.29 (1H, d, *J* = 8.7 Hz), 8.03 (1H, dd, *J* = 8.7, 2.8 Hz). A solution of 5-nitro-2-iodobenzoic acid (293 mg, 1.0 mmol) in thionyl chloride (1.3 mL) was heated at reflux with stirring for 2 h. The resulting solution was concentrated under reduced pressure. The remaining thionyl chloride was removed by azeotropic distillation with benzene. The residue was dissolved in anhydrous CH_2Cl_2 (3.3 mL). To the mixture were added isopropylamine (71 mg, 1.2 mmol) and triethylamine (304 mg, 3.0 mmol) at 0 °C under a nitrogen atmosphere. After stirring at room temperature for 21 h, the resulting solution was diluted with EtOAc. The mixture was washed with 10% HCl, saturated aqueous $NaHCO_3$, water, and brine; dried over Na_2SO_4; filtered; and concentrated under reduced pressure. The residue was purified by recrystallization from hexane and $CHCl_3$ to give **22** (69 mg, 21% in 2 steps) as colorless needles: mp 196–198 °C (hexane/$CHCl_3$); IR (KBr) *ν* 3262, 3087, 2972, 1643, 1606, 1538, 1454, 1351, 1304, 1153, 1021, 905, 864, 839, 826, 737, 730 cm^{-1}; ^{1}H NMR (400 MHz, $CDCl_3$) δ 8.19 (1H, d, *J* = 2.8 Hz), 8.07 (1H, d, *J* = 8.7 Hz), 7.92 (1H, dd, *J* = 8.7,

2.8 Hz), 5.64 (1H, br d, J = 6.0 Hz), 4.38–4.26 (1H, m), 1.32 (6H, d, J = 6.9 Hz); ^{13}C NMR (100 MHz, $CDCl_3$) δ 166.4, 147.8, 143.9, 141.1, 124.9, 122.6, 101.0, 42.6, 22.5; HRMS (EI) calcd for $C_{10}H_{11}IN_2O_3$ ($[M]^+$): 333.9815; found 333.9812.

2-Iodo-*N*-isopropyl-4-methoxybenzamide (**24**)

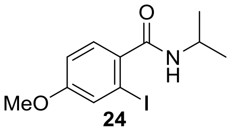

24

A solution of sodium nitrite (105 mg, 1.52 mmol) in water (0.76 mL) was added dropwise to a solution of 2-amino-4-methoxybenzoic acid (127 mg, 0.76 mmol) in a mixture of water (1.9 mL), acetone (0.6 mL), and concentrated HCl (0.4 mL) at 0 ºC. After stirring for 2 h, potassium iodide (252 mg, 1.52 mmol) was added to the mixture. The resulting solution was stirred at 0 ºC for 0.5 h, heated at 90 ºC for 10 min, and then allowed to cool to room temperature. The mixture was extracted with $CHCl_3$. The organic layer was washed with saturated aqueous $Na_2S_2O_3$ and water, dried over Na_2SO_4, filtered, and concentrated under reduced pressure to give crude 2-iodo-4-methoxybenzoic acid (829 mg) as a colorless solid, which was suspended in thionyl chloride (1.6 mL). The mixture was heated at reflux with stirring for 2 h. The resulting solution was concentrated under reduced pressure. The remaining thionyl chloride was removed by azeotropic distillation with benzene. The residue was dissolved in anhydrous CH_2Cl_2 (2.2 mL). To the mixture were added isopropylamine (46 mg, 0.78 mmol) and triethylamine (197 mg, 1.95 mmol) at 0 °C under a nitrogen atmosphere. After stirring at room temperature for 17 h, the resulting solution was diluted with EtOAc. The mixture was washed with 10% HCl, saturated aqueous $NaHCO_3$, water, and brine; dried over Na_2SO_4; filtered; and concentrated under reduced pressure. The residue was purified by recrystallization from hexane and $CHCl_3$ to give **24** (97 mg, 40% in 3 steps) as colorless needles: mp 132.5–135 °C (hexane/$CHCl_3$); IR (KBr) ν 3299, 2971, 2938, 1635, 1597, 1541, 1484, 1467, 1456, 1440, 1348, 1288, 1231, 1177, 1035, 1024, 881, 847, 822, 798, 773 cm^{-1}; ^{1}H NMR (400 MHz, $CDCl_3$) δ 7.37 (1H, d, J = 2.3 Hz), 7.35 (1H, d, J = 8.7 Hz), 6.89 (1H, dd, J = 8.7, 2.3 Hz), 5.58 (1H, br d, J = 5.0 Hz), 4.34–4.22 (1H, m), 3.80 (3H, s) 1.28 (6H, d, J = 6.4 Hz); ^{13}C NMR (100 MHz, $CDCl_3$) δ 168.1, 160.5, 134.7, 129.3, 125.0, 114.0, 92.9, 55.6, 42.2, 22.6; HRMS (EI) calcd for $C_{11}H_{14}INO_2$ ($[M]^+$): 319.0070; found 319.0078.

2-Iodo-*N*-isopropyl-3-methoxybenzamide (**25**)

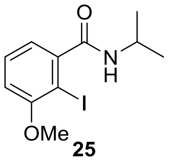

25

A solution of sodium nitrite (166 mg, 2.4 mmol) in water (1.2 mL) was added dropwise to a solution of 2-amino-3-methoxybenzoic acid (201 mg, 1.2 mmol) in a mixture of water (3 mL), acetone (1 mL), and concentrated HCl (0.6 mL) at 0 ºC. After stirring for 2 h, potassium iodide (398 mg, 2.4 mmol) was added to the mixture. The resulting solution was stirred at 0 °C for 0.5 h, heated at 90 °C for 10 min, and then allowed to cool to room temperature. The mixture was extracted with $CHCl_3$. The organic layer was washed with saturated aqueous $Na_2S_2O_3$ and water, dried over Na_2SO_4, filtered, and concentrated under reduced pressure. The residue was purified by recrystallization from hexane and $CHCl_3$ to give 2-iodo-3-methoxybenzoic acid [6] (187 mg, 56%) as a colorless solid: ^{1}H NMR (400 MHz, $CDCl_3$) δ 7.49 (1H, dd, J = 7.8, 1.4 Hz), 7.39 (1H, dd, J = 8.2, 7.8 Hz), 6.99 (1H, dd, J = 8.2, 1.4 Hz), 3.94 (3H, s). A solution of 2-iodo-3-methoxybenzoic acid (181 mg, 0.65 mmol) in thionyl chloride (1.6 mL) was heated at reflux with stirring for 2 h. The resulting solution was concentrated under reduced pressure. The remaining thionyl chloride was removed by azeotropic distillation with benzene. The residue was dissolved in anhydrous CH_2Cl_2 (2.2 mL). To the mixture were added isopropylamine (46 mg, 0.78 mmol) and triethylamine (197 mg, 1.95 mmol) at 0 °C under a nitrogen atmosphere. After stirring at room temperature for 17 h,

the resulting solution was diluted with EtOAc. The mixture was washed with 10% HCl, saturated aqueous $NaHCO_3$, water, and brine; dried over Na_2SO_4; filtered; and concentrated under reduced pressure. The residue was purified by recrystallization from hexane and $CHCl_3$ to give **25** (97 mg, 47% in 2 steps) as colorless prisms: mp 136–137.5 °C (hexane/$CHCl_3$); IR (KBr) *v* 3281, 2975, 2934, 2840, 1632, 1561, 1540, 1465, 1418, 1354, 1331, 1300, 1264, 1201, 1171, 1153, 1128, 1062, 1014, 789, 758, 713, 687 cm^{-1}; ^1H NMR (400 MHz, $CDCl_3$) δ 7.31 (1H, t, *J* = 7.8 Hz), 6.96 (1H, dd, *J* = 7.8, 0.9 Hz), 6.82 (1H, dd, *J* = 7.8, 0.9 Hz), 5.54 (1H, br d, *J* = 6.4 Hz), 4.36–4.24 (1H, m), 3.90 (3H, s), 1.28 (6H, d, *J* = 6.4 Hz); ^{13}C NMR (100 MHz, $CDCl_3$) δ 168.7, 158.2, 144.8, 129.7, 120.3, 111.4, 85.2, 56.7, 42.1, 22.6; HRMS (EI) calcd for $C_{11}H_{14}INO_2$ ($[M]^+$): 319.0070; found 319.0069.

*Typical experimental procedure for oxidation of secondary alcohols **14a–f*** [7]

Secondary alcohol **14** (0.50 mmol) was added to a solution of the catalyst (0.15 mmol) and Bu_4NHSO_4 (170 mg, 0.50 mmol) in a mixture of $MeNO_2$ (1.6 mL) and water (0.6 mL), followed by Oxone® (768 mg. 1.25 mmol) at room temperature (25 ºC). After **14** was completely consumed as indicated by TLC, the resulting mixture was diluted with EtOAc and washed with water. The organic layer was then washed with saturated aqueous $Na_2S_2O_3$ and saturated aqueous $NaHCO_3$, dried over $MgSO_4$; filtered; and concentrated under reduced pressure. The residue was purified by silica gel column chromatography to give pure ketone **15** and the catalyst. All ketones **15** were directly identified by comparison with the commercial samples.

Benzophenone (**15a**)

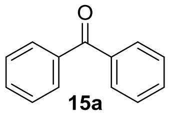

15a

^1H NMR (400 MHz, $CDCl_3$) δ 7.82–7.79 (4H, m), 7.61–7.56 (2H, m), 7.50–7.46 (4H, m); ^{13}C NMR (100 MHz, $CDCl_3$) δ 196.7, 137.5, 132.4, 130.0, 128.2.

4,4'-Difluorobenzophenone (**15b**)

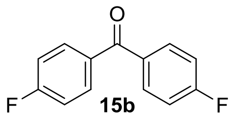

15b

^1H NMR (400 MHz, $CDCl_3$) δ 7.84–7.79 (4H, m), 7.20–7.14 (4H, m); ^{13}C NMR (100 MHz, $CDCl_3$) δ 193.8, 165.4 (d, *J* = 254 Hz), 133.7 (d, *J* = 2.9 Hz), 132.5 (d, *J* = 9.6 Hz), 115.5 (d, *J* = 22.0 Hz).

1-Indanone (**15c**)

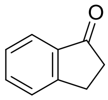

15c

^1H NMR (400 MHz, $CDCl_3$) δ 7.76 (1H, d, *J* = 7.3 Hz), 7.59 (1H, td, *J* = 7.3, 1.4 Hz), 7.50–7.47 (1H, m), 7.39–7.35 (1H, m), 3.16–3.13 (2H, m), 2.71–2.68 (2H, m); ^{13}C NMR (100 MHz, $CDCl_3$) δ 207.1, 155.1, 137.0, 134.6, 127.2, 126.6, 123.7, 36.2, 25.7.

Acetophenone (**15d**)

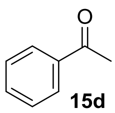

15d

^1H NMR (500 MHz, $CDCl_3$) δ 7.98–7.95 (2H, m), 7.58–7.55 (1H, m), 7.49–7.45 (2H, m), 2.61 (3H, s); ^{13}C NMR (126 MHz, $CDCl_3$) δ 198.2, 137.1, 133.1, 128.5, 128.3, 26.6.

2-Phenylacetophenone (**15e**)

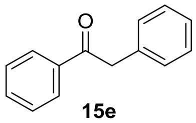

15e

^{1}H NMR (500 MHz, $CDCl_3$) δ 8.02–8.00 (2H, m), 7.56–7.53 (1H, m), 7.46–7.43 (2H, m), 7.34–7.31 (2H, m), 7.28–7.23 (3H, m), 4.28 (2H, s); ^{13}C NMR (126 MHz, $CDCl_3$) δ 197.6, 136.5, 134.5, 133.1, 129.4, 128.64, 128.61, 128.57, 126.9, 45.5.

4-Phenyl-2-butanone (**15f**)

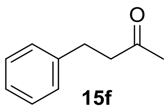

15f

^{1}H NMR (500 MHz, $CDCl_3$) δ 7.29–7.25 (2H, m), 7.21–7.17 (3H, m), 2.89 (2H, t, J = 7.5 Hz), 2.76 (2H, t, J = 7.5 Hz), 2.14 (3H, s); ^{13}C NMR (126 MHz, $CDCl_3$) δ 207.9, 140.9, 128.5, 128.2, 126.1, 45.1, 30.0, 29.7.

Typical experimental procedure for oxidation of primary alcohols ***14g–k*** [7]

Primary alcohol **14** (0.50 mmol) was added to a solution of the catalyst (0.15 mmol) and Bu_4NHSO_4 (170 mg, 0.50 mmol) in a mixture of $MeNO_2$ (1.6 mL) and water (0.6 mL), followed by Oxone® (768 mg. 1.25 mmol) at room temperature (25 °C). After **14** were completely consumed as indicated by TLC, the resulting mixture was diluted with EtOAc, water, and saturated aqueous $Na_2S_2O_3$. The organic layer was then washed with saturated aqueous $Na_2S_2O_3$, saturated aqueous $NaHCO_3$, and brine; dried over $MgSO_4$; filtered; and concentrated under reduced pressure. The residue was purified by silica gel column chromatography to give the catalyst. The combined aqueous layers were acidified with 10% HCl and extracted with EtOAc. The organic layer was washed with brine, dried over $MgSO_4$, filtered, and concentrated under reduced pressure. The residue was purified by silica gel column chromatography to give pure carboxylic acid **26**. All carboxylic acids **26** were directly identified by comparison with the commercial samples.

4-Nitrobenzoic acid (**26g**)

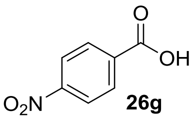

26g

^{1}H NMR (400 MHz, $CDCl_3$/DMSO-d_6 = 4/1) δ 8.28 (2H, dt, J = 8.7, 2.3 Hz), 8.21 (2H, dt, J = 8.7, 2.3 Hz); ^{13}C NMR (100 MHz, $CDCl_3$/DMSO-d_6 = 4/1) δ 165.2, 149.1, 135.7, 129.7, 122.3.

4-Chlorobenzoic acid (**26h**)

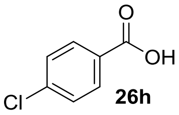

26h

^{1}H NMR (400 MHz, $CDCl_3$/DMSO-d_6 = 4/1) δ 7.99–7.95 (2H, m), 7.44–7.40 (2H, m); ^{13}C NMR (100 MHz, $CDCl_3$/DMSO-d_6 = 4/1) δ 166.1, 137.5, 130.1, 128.6, 127.4.

Monomethyl terephthalate (**26i**)

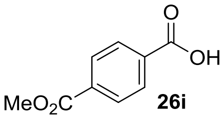

26i

^{1}H NMR (400 MHz, $CDCl_3$/DMSO-d_6 = 4/1) δ 8.12–8.06 (4H, m), 3.94 (3H, s); ^{13}C NMR (100 MHz, $CDCl_3$/DMSO-d_6 = 4/1) δ 166.8, 165.6, 134.3, 132.9, 129.0, 128.7, 51.7.

3-Phenylpropionic acid (**26j**)

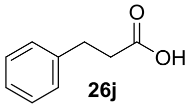

26j

^{1}H NMR (500 MHz, $CDCl_3$) δ 7.32–7.28 (2H, m), 7.23–7.20 (3H, m), 2.97 (2H, t, J = 8.0 Hz), 2.69 (2H, t, J = 8.0 Hz); ^{13}C NMR (126 MHz, $CDCl_3$) δ 178.4, 140.1, 128.5, 128.2, 126.4, 35.5, 30.6.

Cyclohexanecarboxylic acid (**26k**)

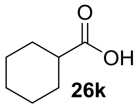

26k

^{1}H NMR (500 MHz, $CDCl_3$) δ 2.33 (1H, tt, *J* = 11.5, 4.0 Hz), 1.97–1.91 (2H, m), 1.79–1.74 (2H, m), 1.67–1.62 (1H, m), 1.50–1.42 (2H, m), 1.34–1.19 (3H, m); ^{13}C NMR (126 MHz, $CDCl_3$) δ 182.4, 42.9, 28.8, 25.7, 25.3.

Preparation of 2-iodoxybenzamide

2-Iodyl-5-methoxy-*N*-isopropylbenzamide (**29**)

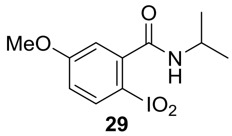

29

According to the literature [8], 0.065 M dimethyldioxirane in acetone (40 mL, 2.6 mmol) was added to a solution of **17** (275 mg, 0.86 mmol) in anhydrous CH_2Cl_2 (4 mL) at room temperature under nitrogen atmosphere. After stirring for 9.5 h, the precipitate was collected by filtration, washed with anhydrous CH_2Cl_2 and Et_2O, and dried *in vacuo* to give **29** (221 mg, 73%) as a colorless solid: mp 164 °C (dec.); IR (KBr) *ν* 3417 (br), 3362, 3208 (br), 3060 (br), 2975, 2942, 1623, 1609, 1588, 1572, 1553, 1467, 1440, 1400, 1362, 1335, 1318, 1300, 1251, 1187, 1172, 1142, 1028, 921, 891, 869, 832, 773, 760 cm^{-1}; ^{1}H NMR (400 MHz, DMSO-d_6) δ 9.02 (1H, br d, *J* = 7.8 Hz), 8.17 (1H, d, *J* = 8.7 Hz), 7.77 (1H, d, *J* = 2.8 Hz), 7.45 (1H, dd, *J* = 8.7, 2.8 Hz), 4.19–4.11 (1H, m), 3.90 (3H, s), 1.21 (6H, d, *J* = 6.9 Hz); ^{13}C NMR (125 MHz, DMSO-d_6) δ 164.9, 162.0, 139.9, 130.8, 125.4, 117.7, 112.8, 56.5, 42.4, 22.2.

*In situ generation of trivalent iodine derivative **27** from **29***

Since the synthesis of trivalent iodine derivative **27** according to the reported synthetic procedure for 2-iodosylbenzoic acid **28** [9] was unsuccessful, we examined the in situ generation of **27** by oxidation of 2-propanol to acetone with pentavalent iodine derivative **29**. To a solution of **29** (2.6 mg, 7.5 μmol), $KHSO_4$ (2.6 mg, 18.75 μmol), and Bu_4NHSO_4 (2.6 mg, 7.5 μmol) in a 4:1 mixture of CD_3CN and D_2O (0.75 mL) was added 1 M 2-propanol in D_2O (15 μL, 15 μmol). The mixture was stirred at 40 °C for 143 h and the supernatant was then taken for the NMR measurement. An unknown iodine compound together with pentavalent **29** and monovalent **17** observed in the ^{1}H NMR spectrum (Figure S1). The mixture was then kept at room temperature for ca. 4 months. The ^{1}H NMR spectrum indicated that only **17** and the unknown iodine compound remained in the mixture. The ^{13}C NMR data for **17** and the unknown iodine compound were well matched with those for 5-(*n*-octyloxy)-2-iodobenzoic acid and its trivalent derivative [10], respectively. Therefore, the unknown iodine compound was identified as **27**.

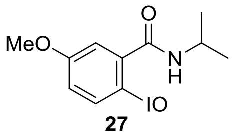

27

27 (as a mixture with $KHSO_4$, Bu_4NHSO_4, 2-propanol, and acetone): ^{1}H NMR (400 MHz, CD_3CN/D_2O = 4/1) δ 7.90 (1H, d, *J* = 2.8 Hz), 7.77 (1H, d, *J* = 9.2 Hz), 7.57 (1H, dd, *J* = 9.2, 2.8 Hz), 4.20 (1H, sep, *J* = 6.4 Hz), 3.91 (3H, s), 1.26 (6H, d, *J* = 6.4 Hz); ^{13}C NMR (100 MHz, CD_3CN/D_2O = 4/1) δ 168.3, 163.3, 129.1, 128.2, 124.1, 114.9, 109.0, 57.4, 45.7, 21.8.

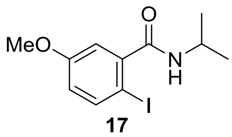

17

17 (as a mixture with $KHSO_4$ and Bu_4NHSO_4): ^{1}H NMR (400 MHz, CD_3CN/D_2O = 4/1) δ 7.69 (1H, d, *J* = 8.7 Hz), 6.86 (1H, d, *J* = 3.2 Hz), 6.73 (1H, dd, *J* = 8.7, 3.2 Hz), 4.05 (1H, sep, *J* = 6.4 Hz), 3.74 (3H, s), 1.17 (6H, d, *J* = 6.4 Hz); ^{13}C NMR (100 MHz, CD_3CN/D_2O = 4/1) δ 170.1, 160.6, 144.4, 141.2, 118.0, 114.8, 81.4, 56.3, 42.8, 22.4.

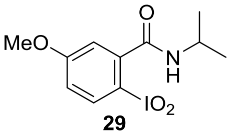

29

29 (as a mixture with $KHSO_4$ and Bu_4NHSO_4): ^{1}H NMR (400 MHz, CD_3CN/D_2O = 4/1) δ 8.19 (1H, d, *J* = 8.7 Hz), 7.65 (1H, d, *J* = 2.8 Hz), 7.46 (1H, dd, *J* = 8.7, 2.8 Hz), 4.22 (1H, sep, *J* = 6.9 Hz), 3.91 (3H, s), 1.24 (6H, d, *J* = 6.9 Hz).

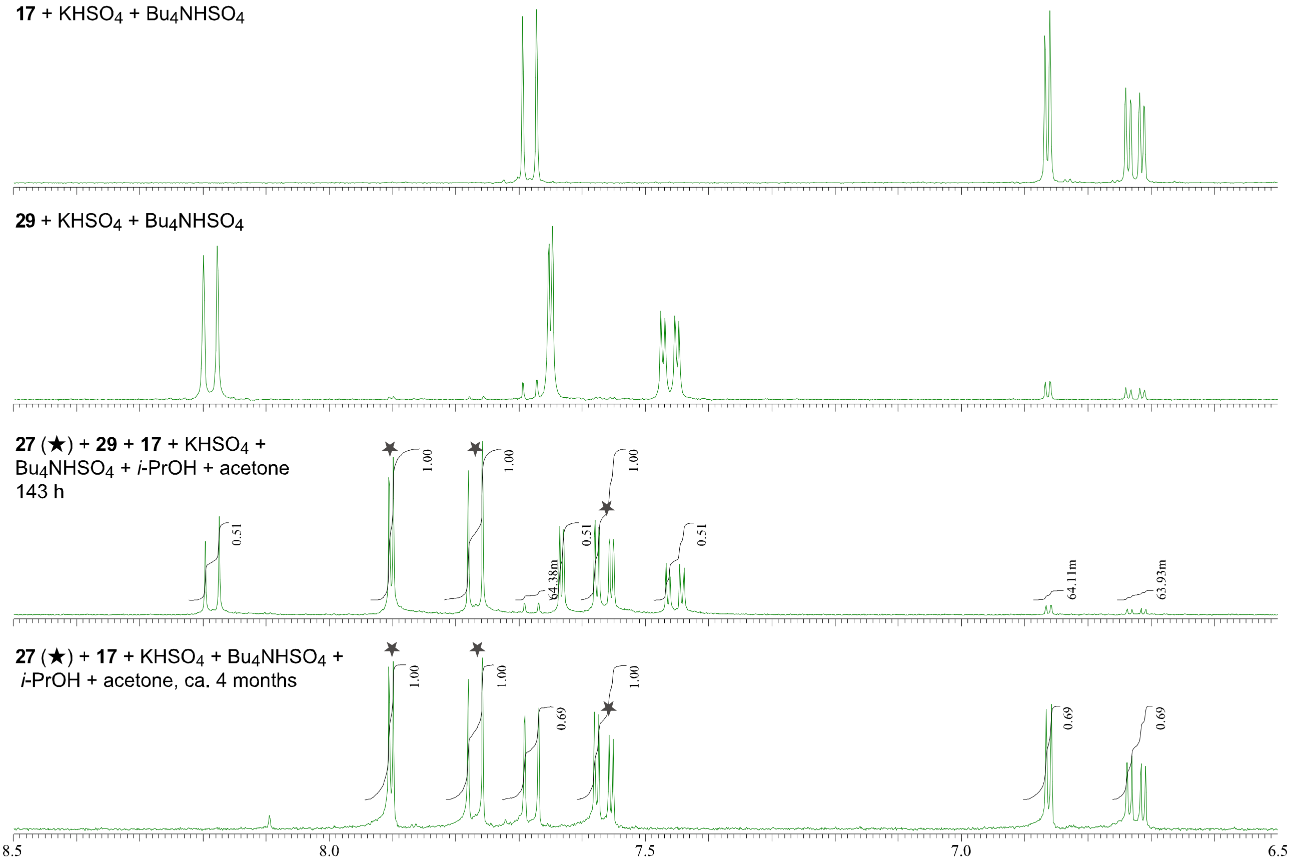

Figure S1. ^{1}H NMR spectra of the reaction mixture of oxidation of 2-propanol with pentavalent iodine derivatives **29**.

Oxidation of monovalent iodine derivatives to pentavalent iodine derivatives with Oxone®

To a solution of monovalent iodine derivative **17** or **3** (7.5 μmol) and Bu_4NHSO_4 (2.6 mg, 7.5 μmol) in a 4:1 mixture of CD_3CN and D_2O (0.75 mL) was added Oxone® (11.5 mg, 18.75 μmol) at room temperature. The supernatant was taken for the NMR measurement after 12, 24, and 36 h (Figure S2 and S3). The NMR spectra of **3**, **28** [9], and **2** [11] in the presence of $KHSO_4$ and Bu_4NHSO_4 in a 4:1 mixture of CD_3CN and D_2O were measured as reference.

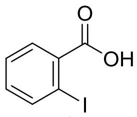

3

3 (as a mixture with $KHSO_4$ and Bu_4NHSO_4): ^{1}H NMR (400 MHz, CD_3CN/D_2O = 4/1) δ 7.98 (1H, dd, *J* = 7.8, 1.4 Hz), 7.76 (1H, dd, *J* = 7.8, 1.8 Hz), 7.44 (1H, td, *J* = 7.8, 1.4 Hz), 7.19 (1H, td, *J* = 7.8, 1.8 Hz).

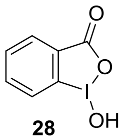

28

28 (as a mixture with $KHSO_4$ and Bu_4NHSO_4): ^{1}H NMR (400 MHz, CD_3CN/D_2O = 4/1) δ 8.08 (1H, dd, *J* = 7.8, 1.4 Hz), 7.92 (1H, ddd, *J* = 8.2, 6.9, 1.4 Hz), 7.85 (1H, dd, *J* = 8.2, 1.4 Hz), 7.69 (1H, ddd, *J* = 7.8, 6.9, 1.4 Hz).

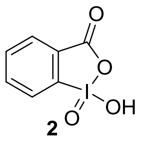

2 (as a mixture with $KHSO_4$ and Bu_4NHSO_4): ^1H NMR (400 MHz, CD_3CN/D_2O = 4/1) δ 8.24 (1H, d, *J* = 7.3 Hz), 8.14 (1H, d, *J* = 7.3 Hz), 8.03 (1H, t, *J* = 7.3 Hz), 7.88 (1H, t, *J* = 7.3 Hz).

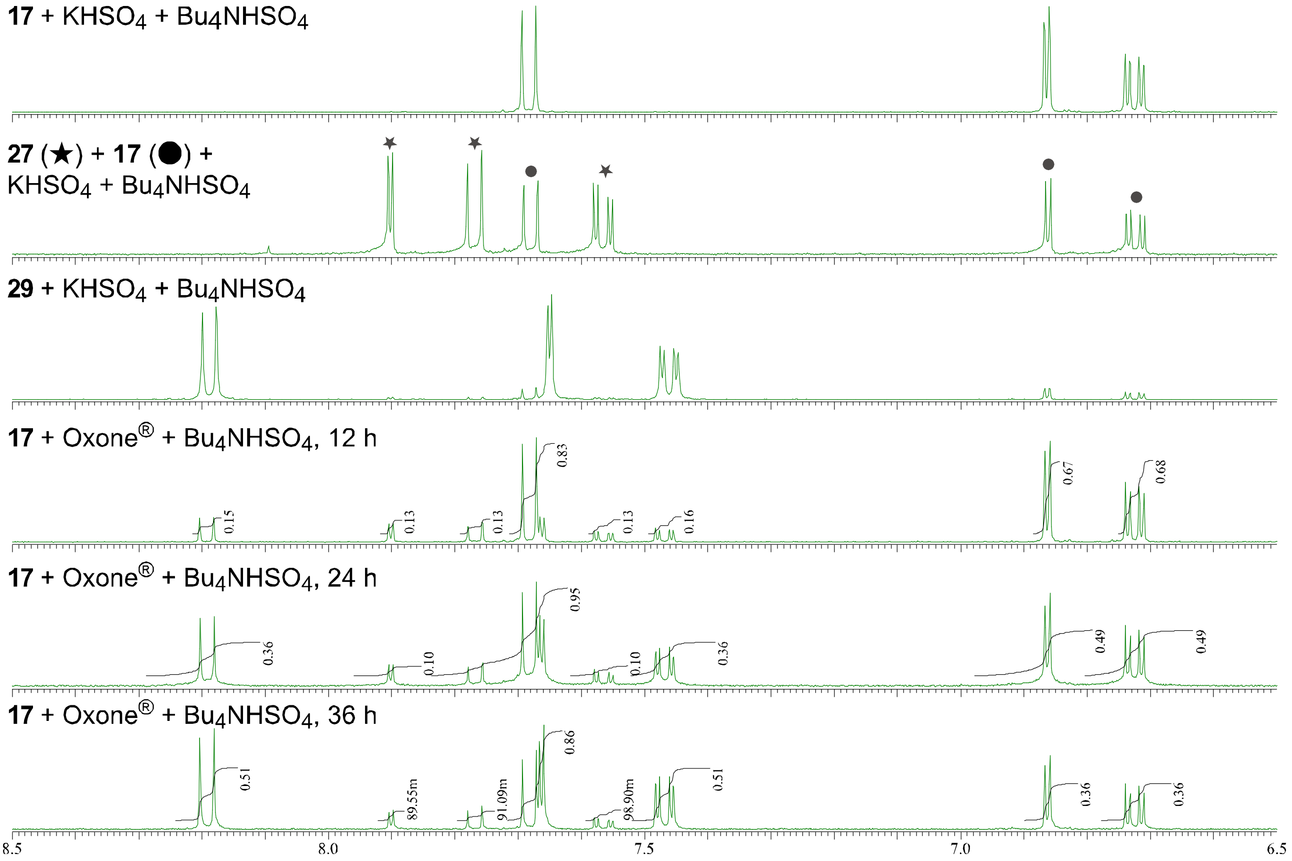

Figure S2. ^1H NMR spectra of the reaction mixture of oxidation of **17** with Oxone® in the presence of Bu_4NHSO_4.

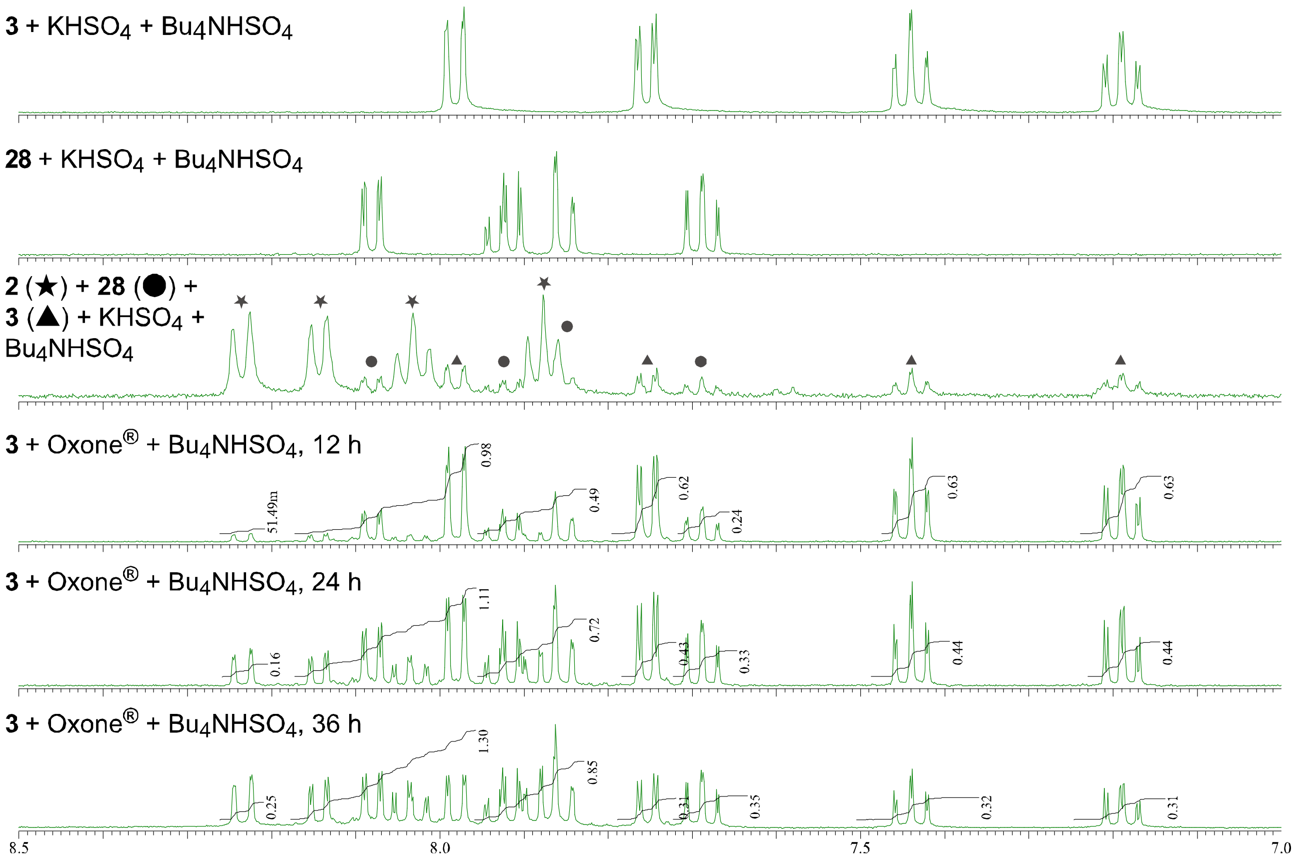

Figure S3. ^{1}H NMR spectra of the reaction mixture of oxidation of **3** with Oxone® in the presence of Bu_4NHSO_4.

3. ^{1}H and ^{13}C NMR Spectra

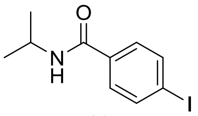

16

^{1}H NMR (400 MHz, $CDCl_3$) of **16**

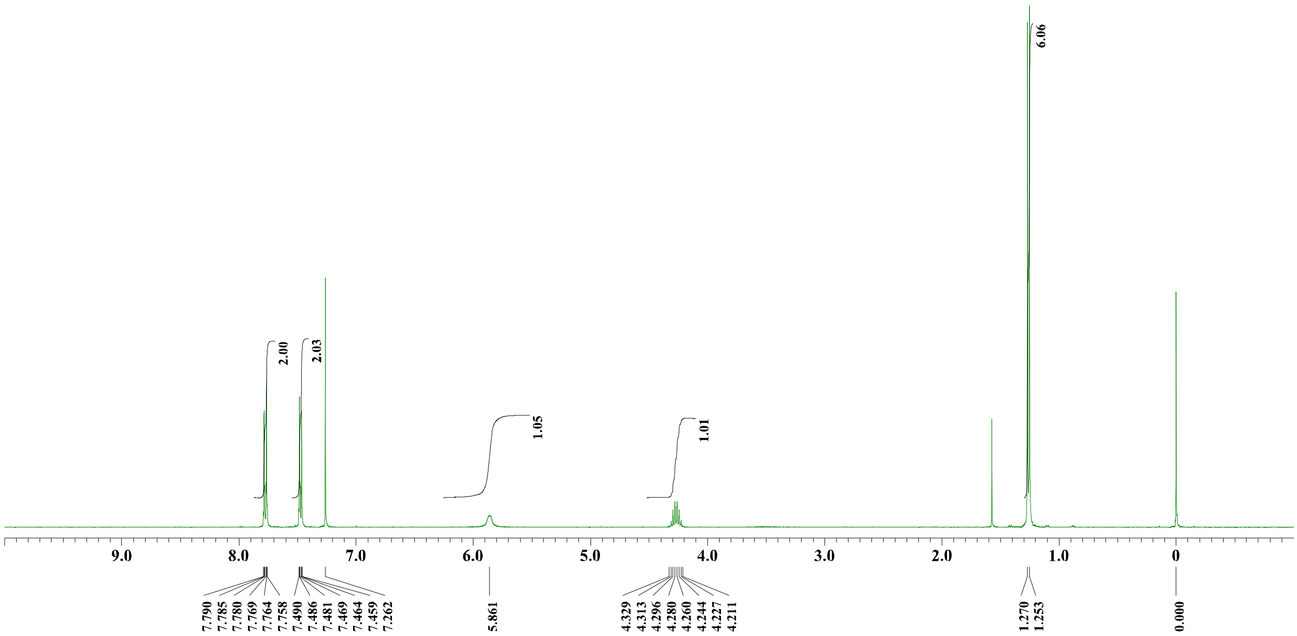

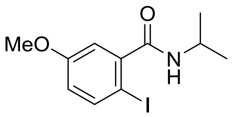

17

^{1}H NMR (500 MHz, $CDCl_3$) of **17**

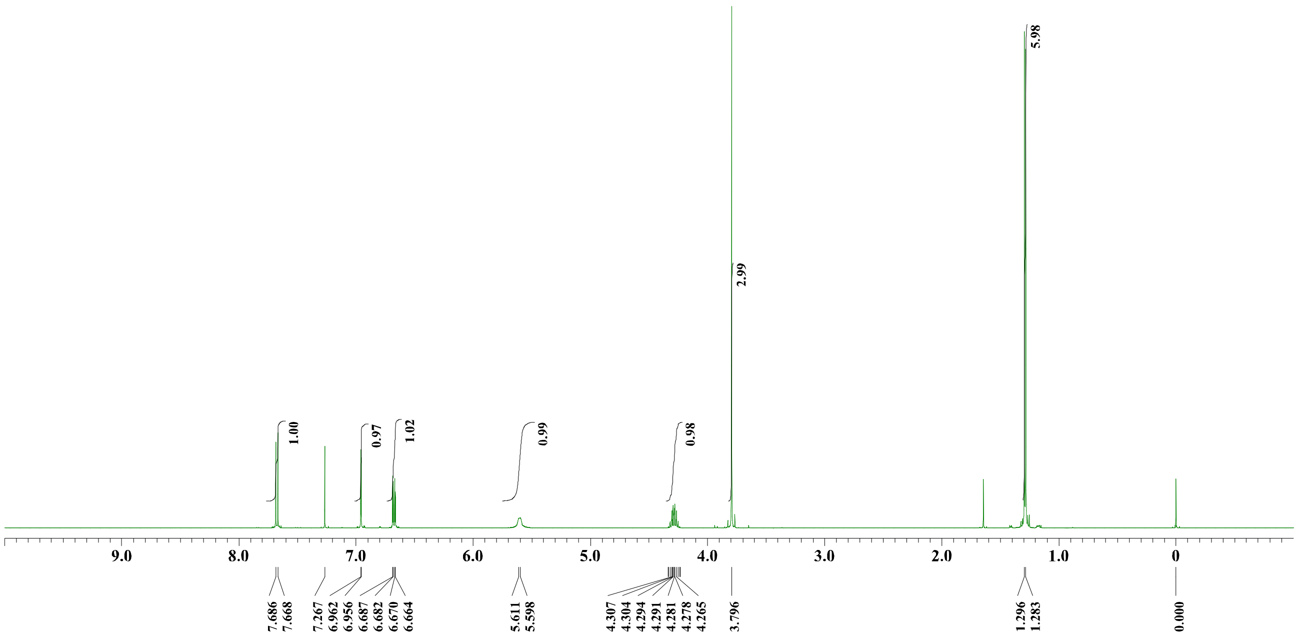

^{13}C NMR (126 MHz, $CDCl_3$) of **17**

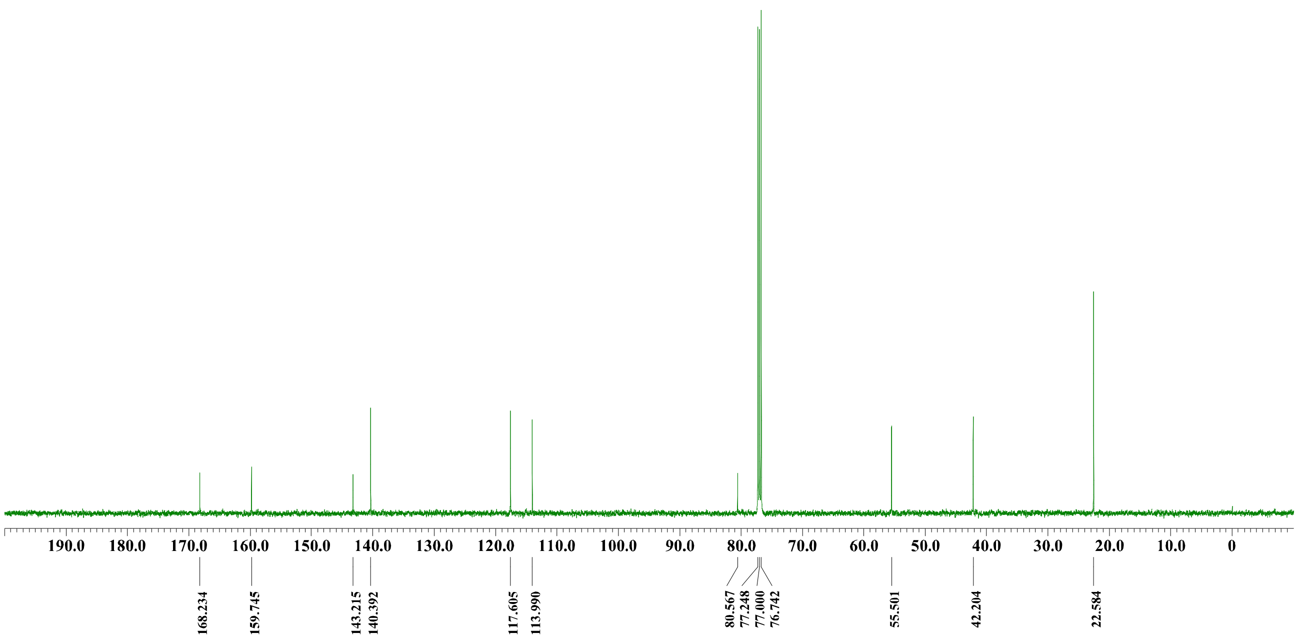

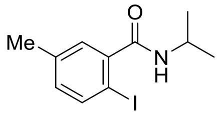

18

^{1}H NMR (400 MHz, $CDCl_3$) of **18**

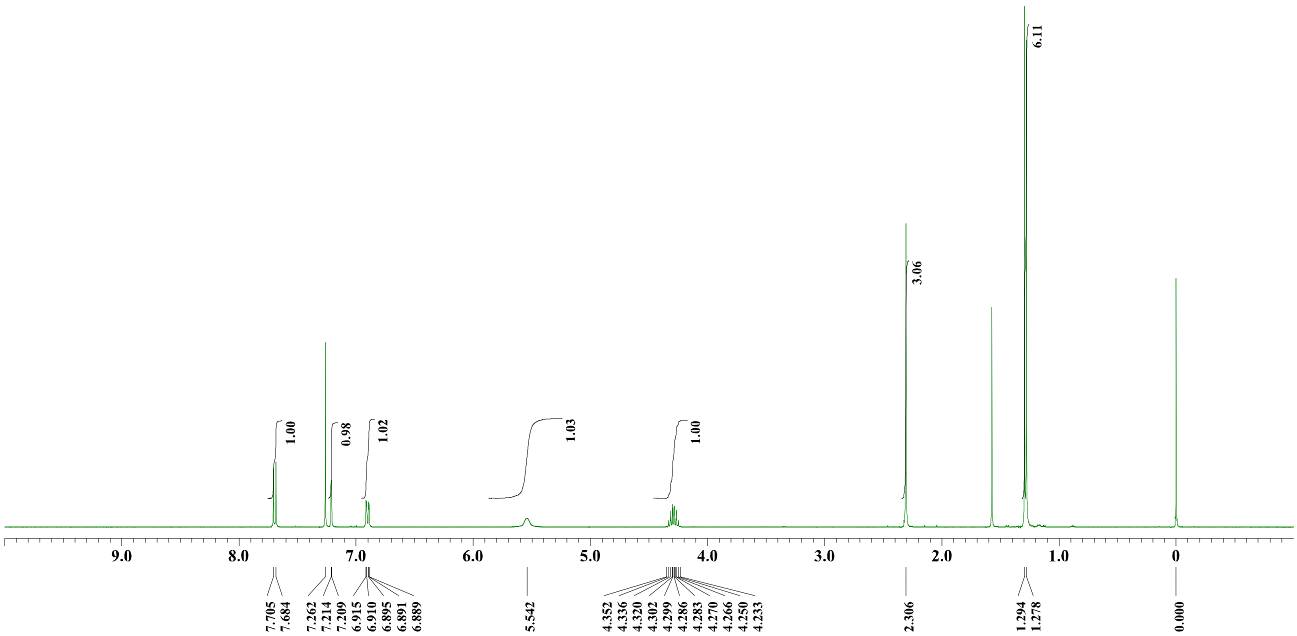

^{13}C NMR (100 MHz, CDCl$_3$) of **18**

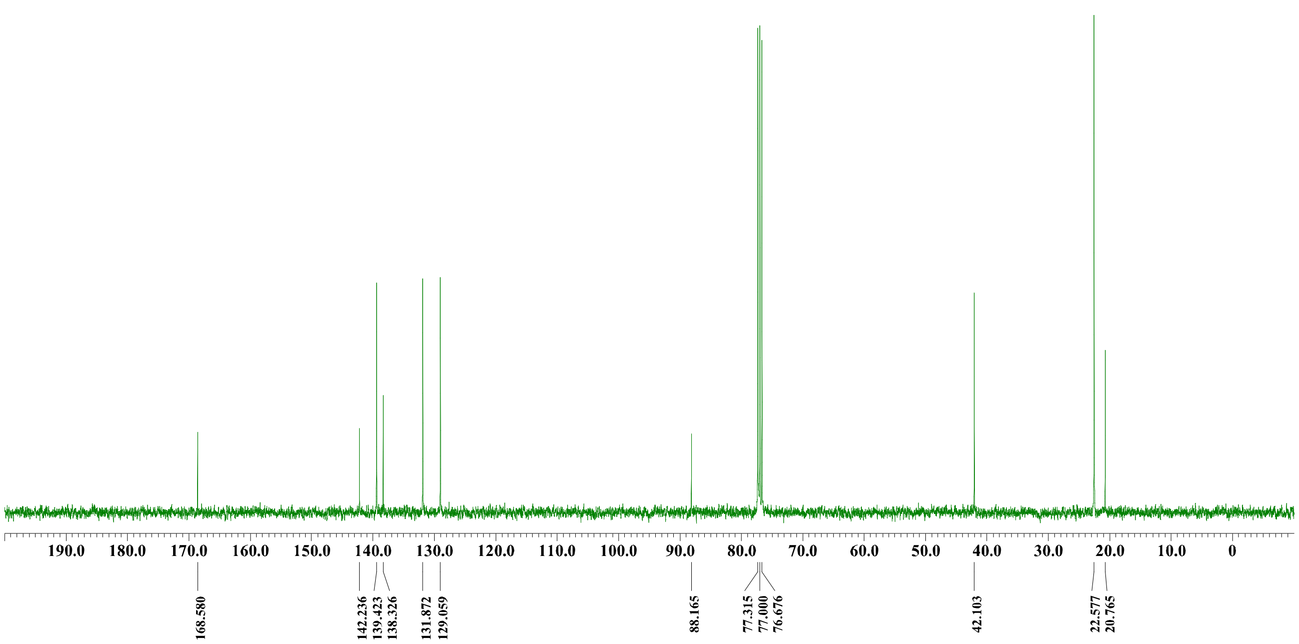

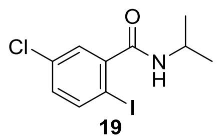

19

^{1}H NMR (400 MHz, CDCl$_3$) of **19**

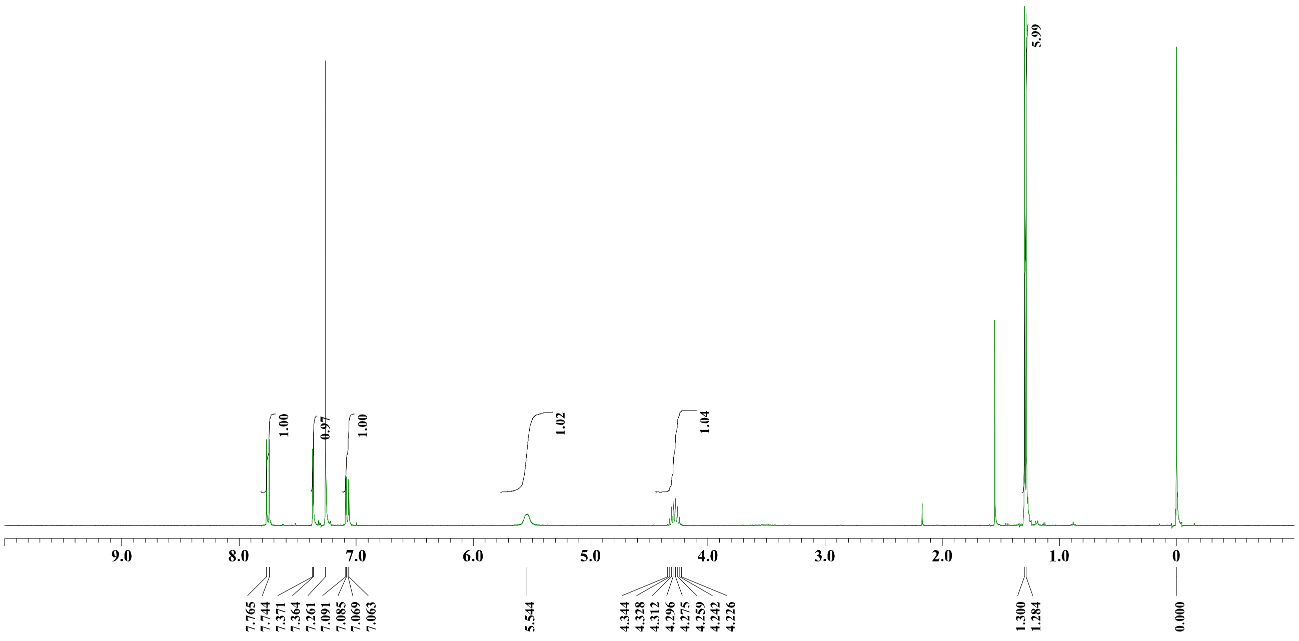

^{13}C NMR (100 MHz, $CDCl_3$) of **19**

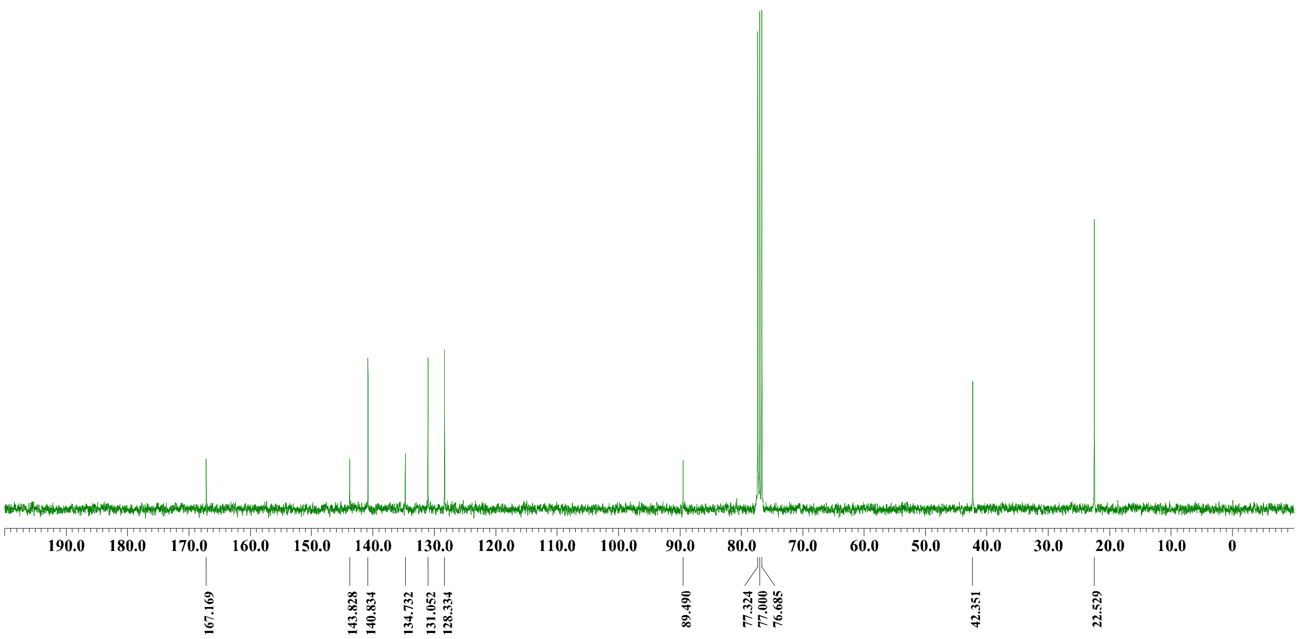

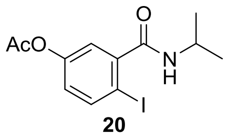

20

^{1}H NMR (400 MHz, $CDCl_3$) of **20**

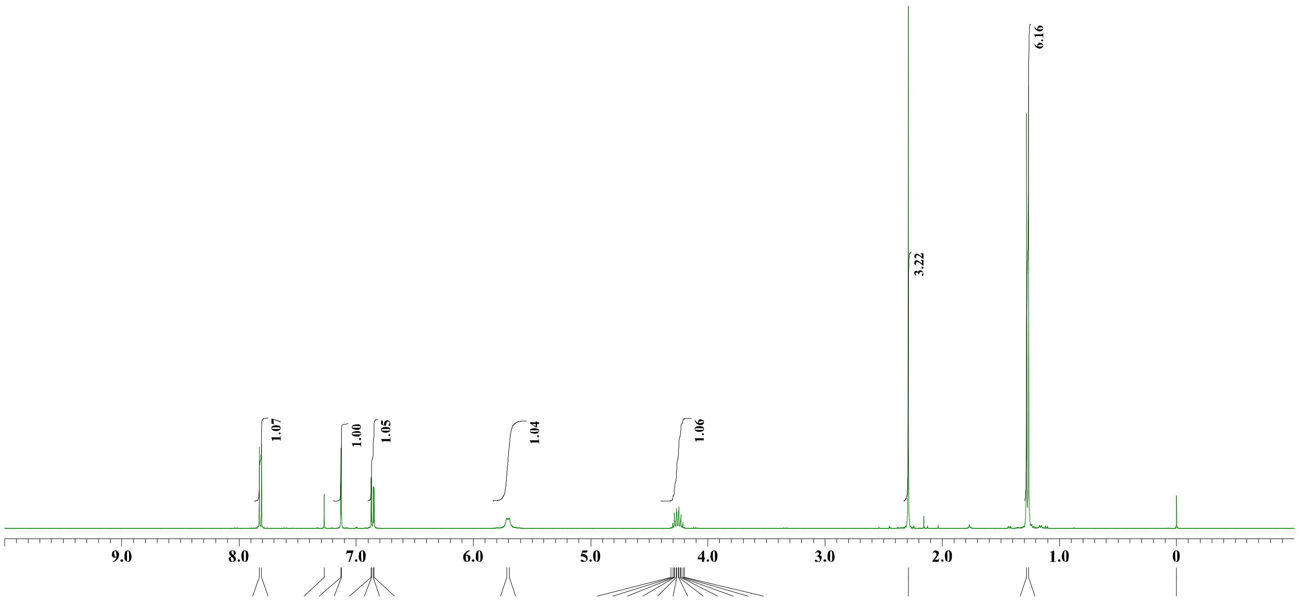

^{13}C NMR (100 MHz, $CDCl_3$) of **20**

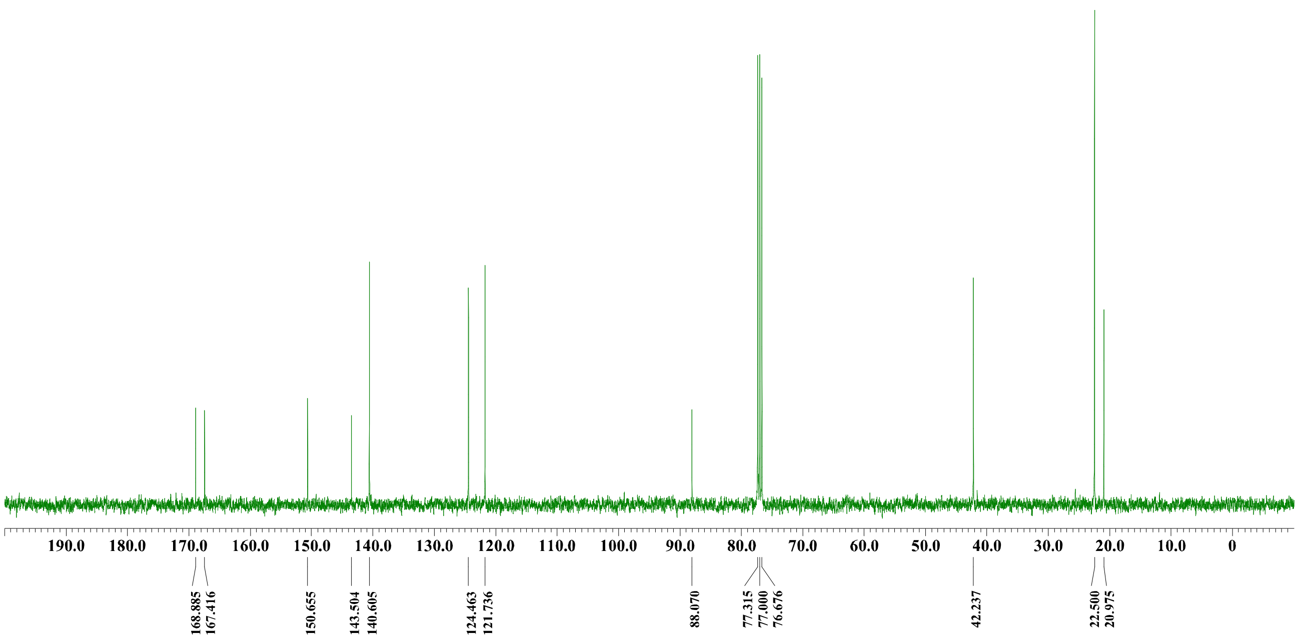

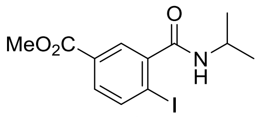

21

^{1}H NMR (400 MHz, $CDCl_3$) of **21**

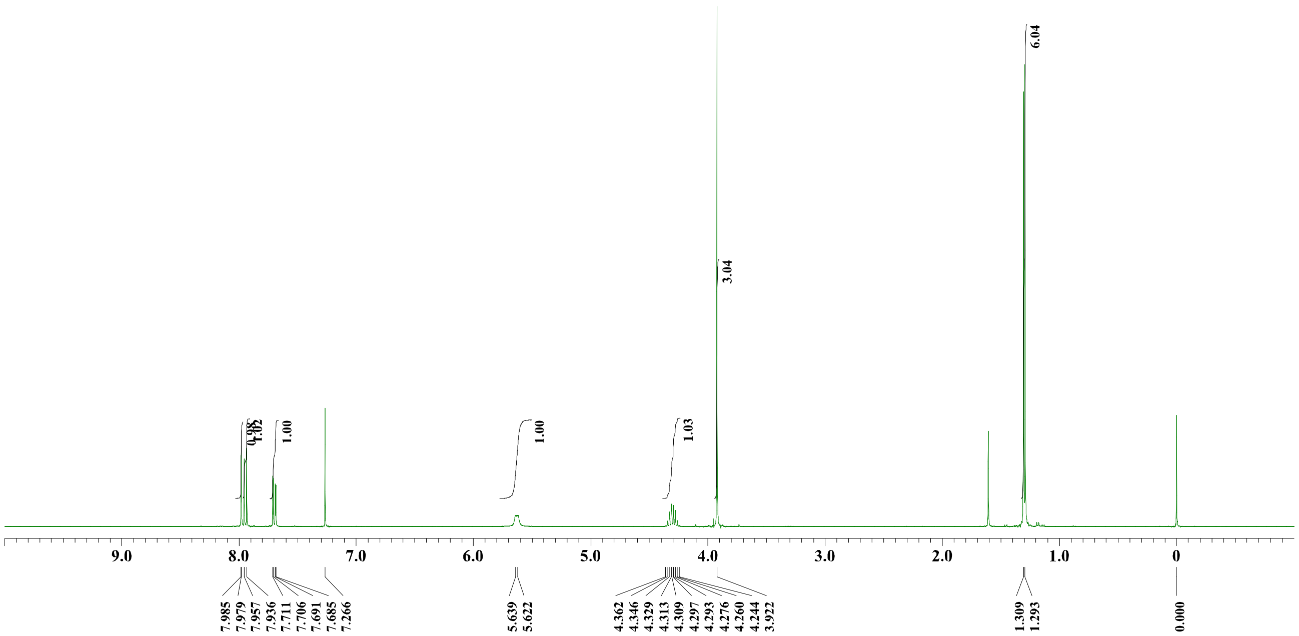

^{13}C NMR (100 MHz, $CDCl_3$) of **21**

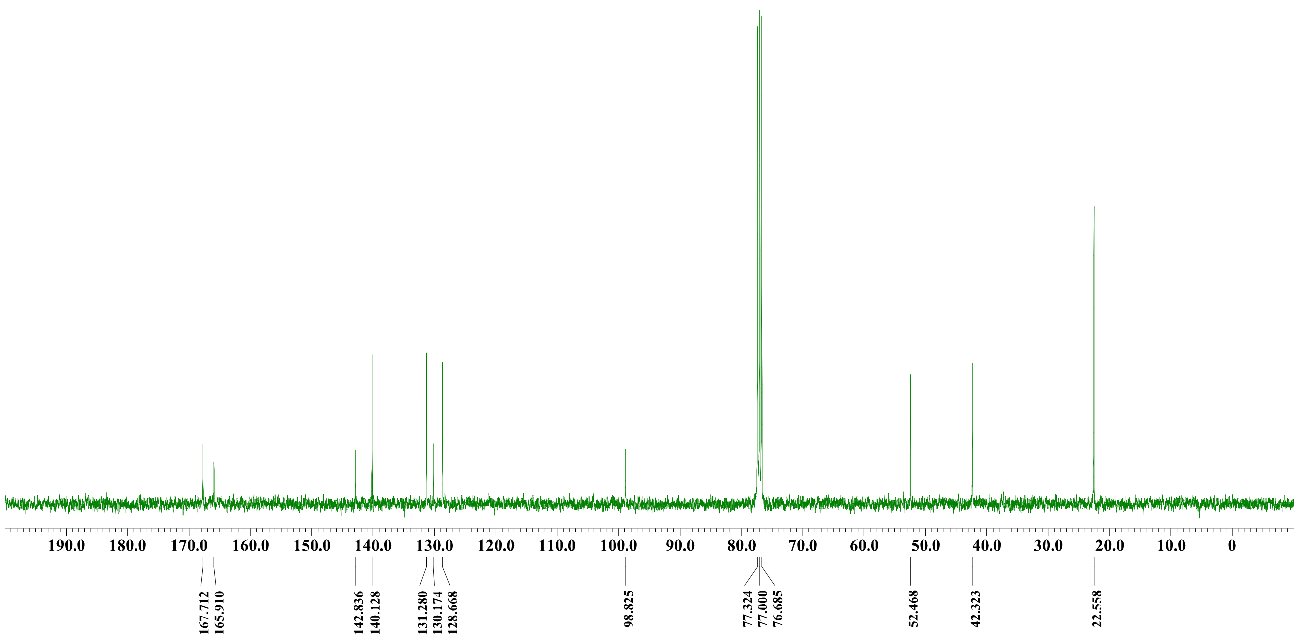

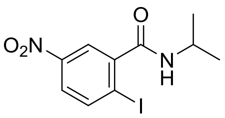

22

^{1}H NMR (400 MHz, $CDCl_3$) of **22**

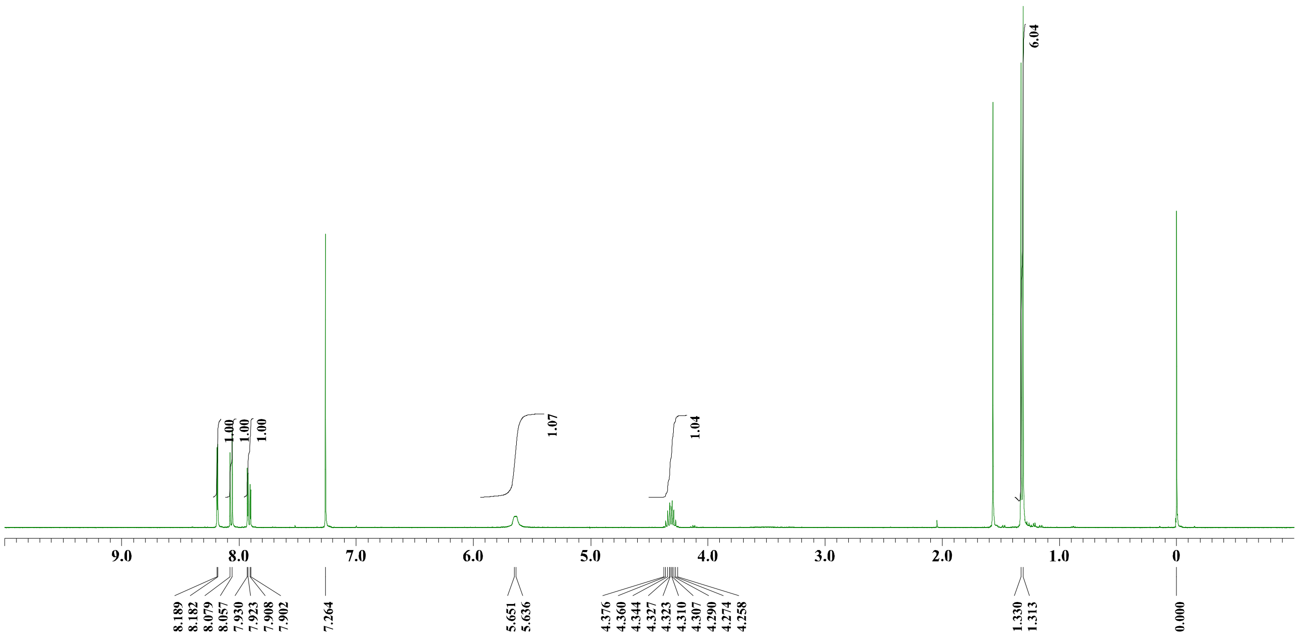

^{13}C NMR (100 MHz, $CDCl_3$) of **22**

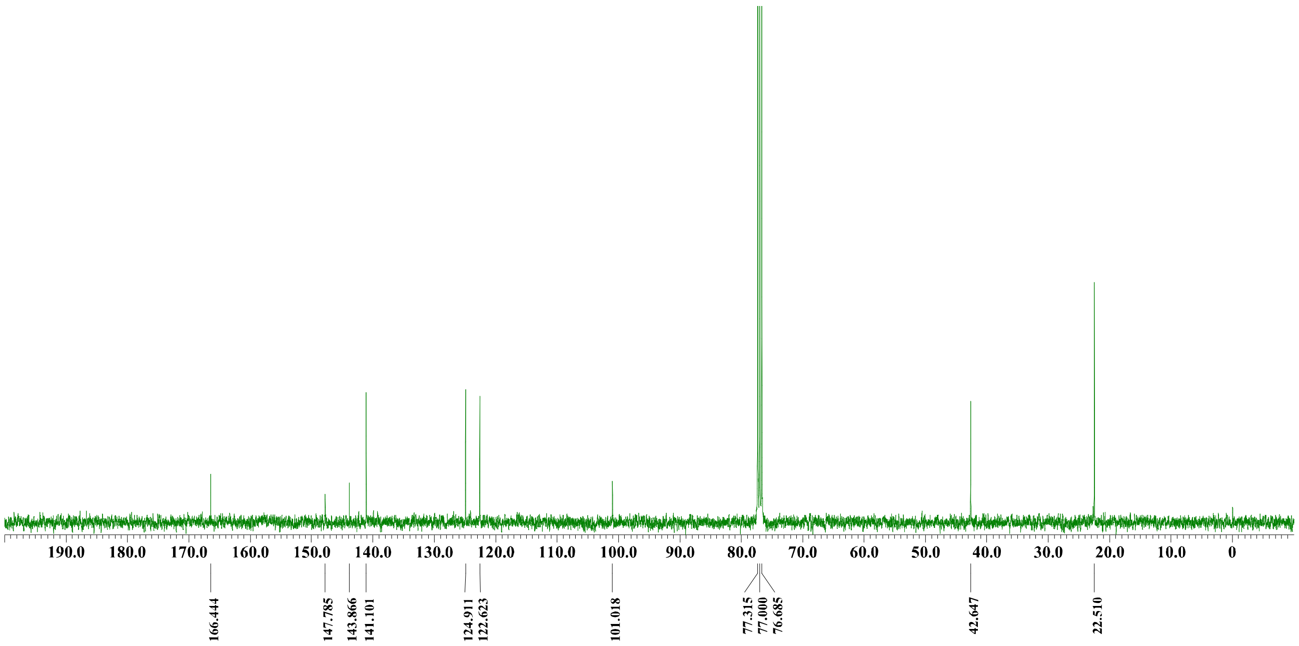

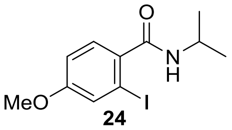

24

^{1}H NMR (400 MHz, $CDCl_3$) of **24**

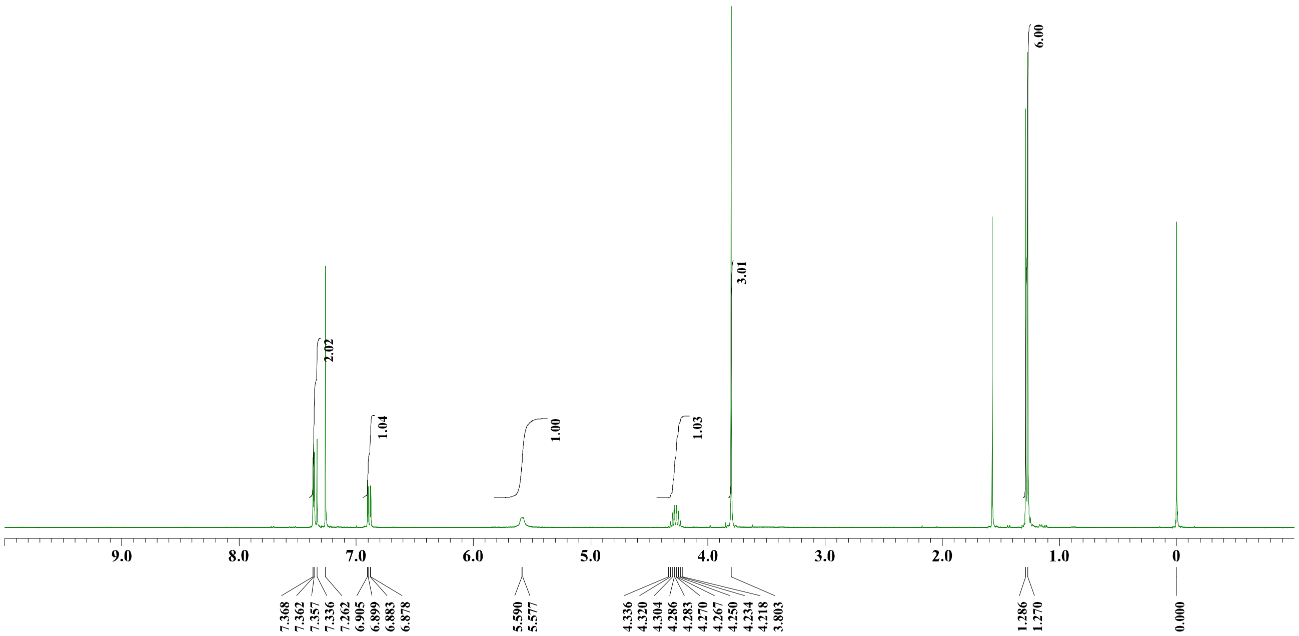

^{13}C NMR (100 MHz, $CDCl_3$) of **24**

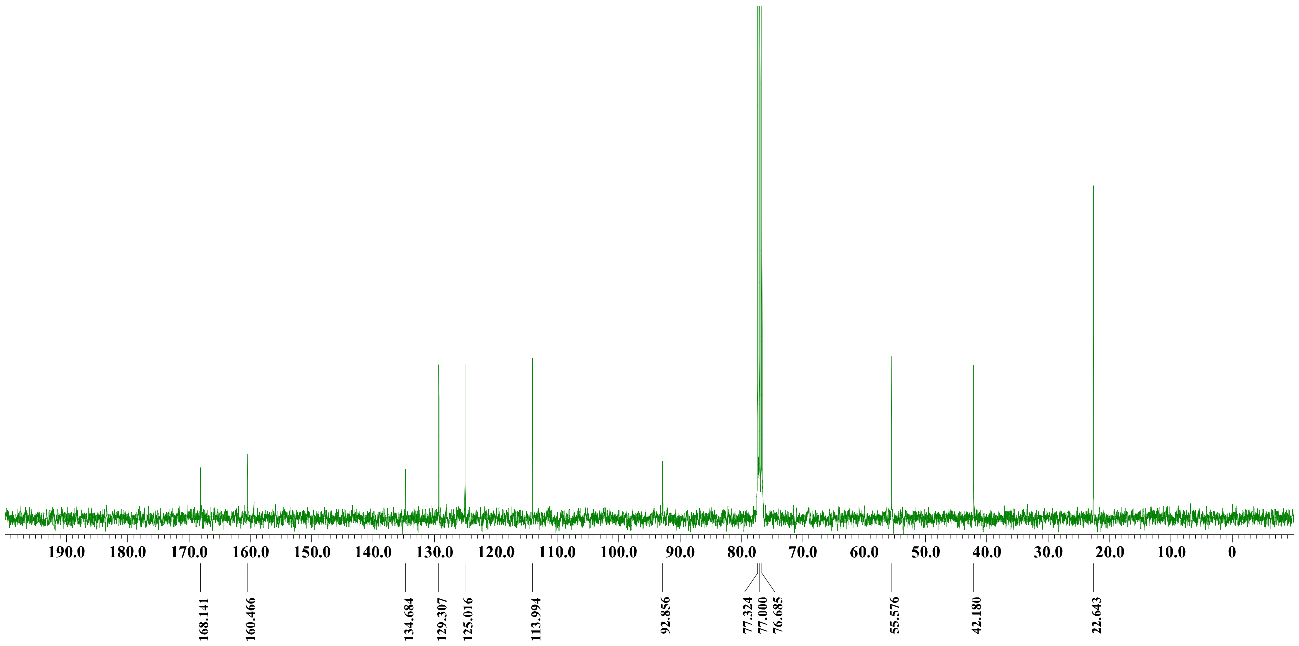

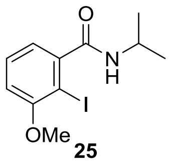

25

^{1}H NMR (400 MHz, $CDCl_3$) of **25**

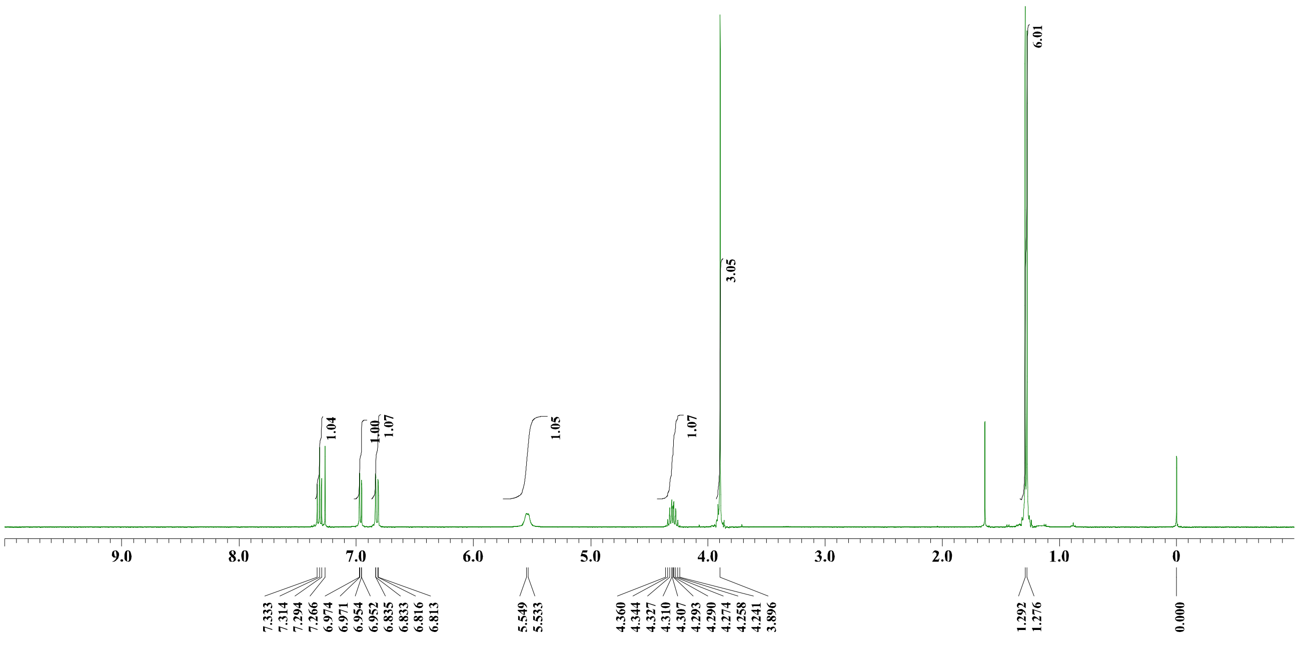

^{13}C NMR (100 MHz, $CDCl_3$) of **25**

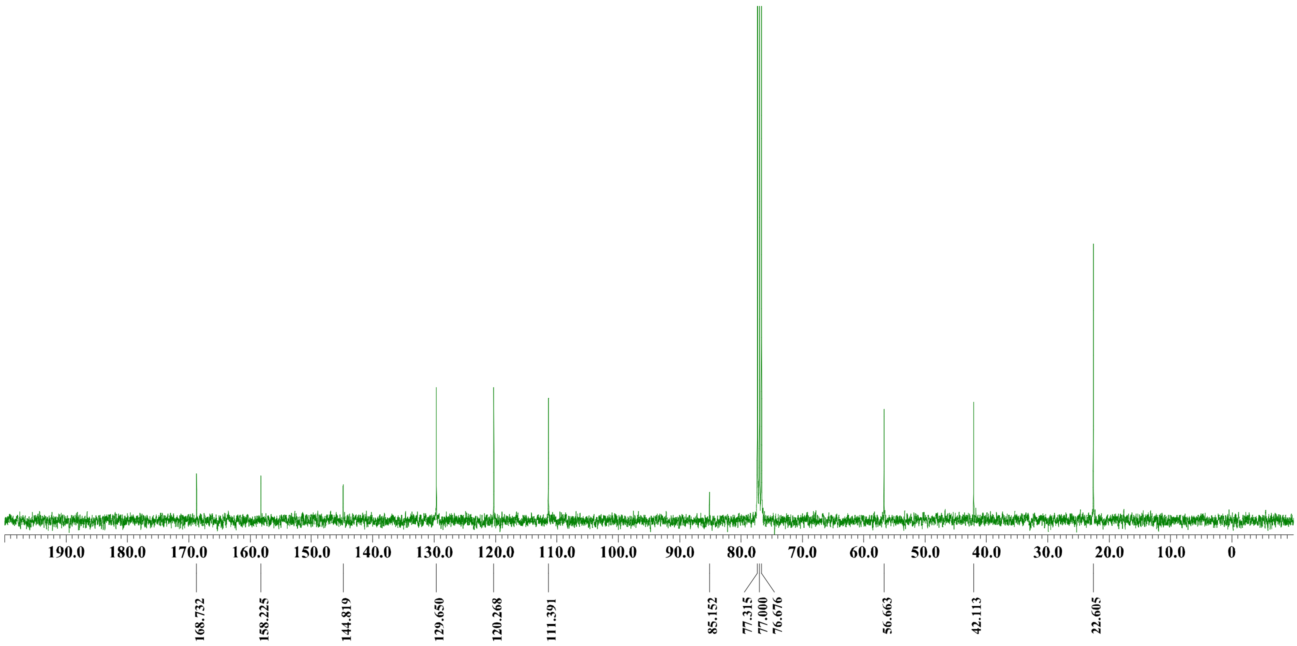

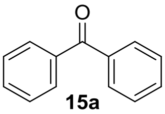

15a

^{1}H NMR (400 MHz, $CDCl_3$) of **15a**

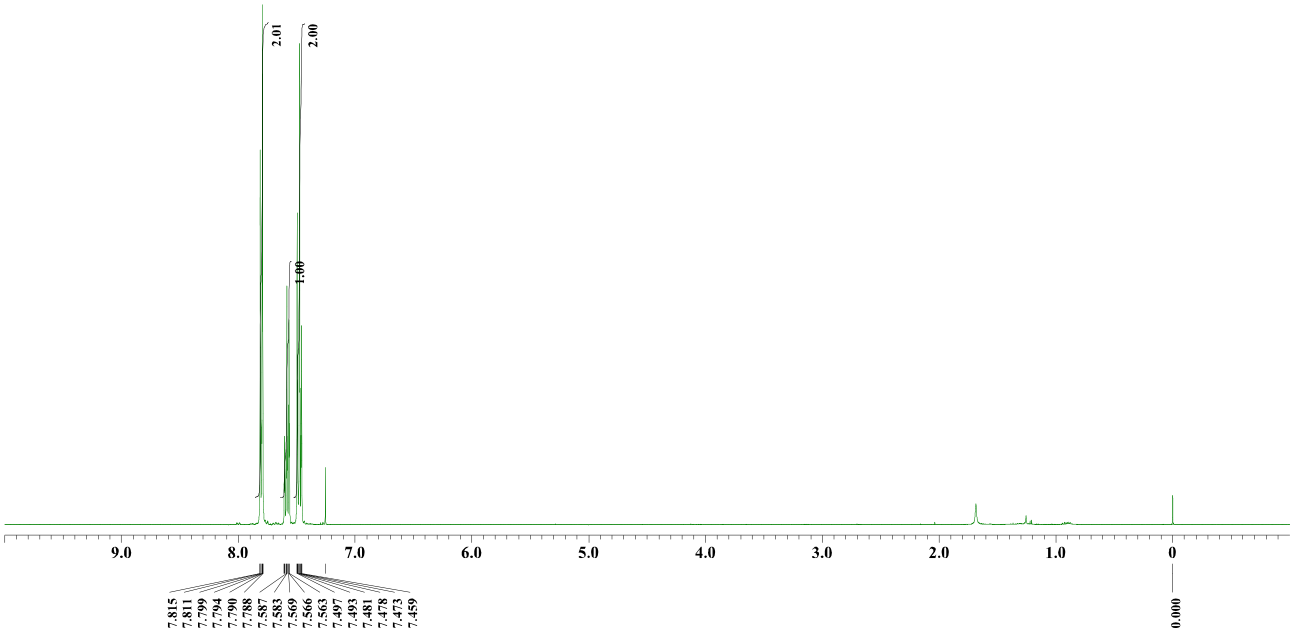

^{13}C NMR (100 MHz, $CDCl_3$) of **15a**

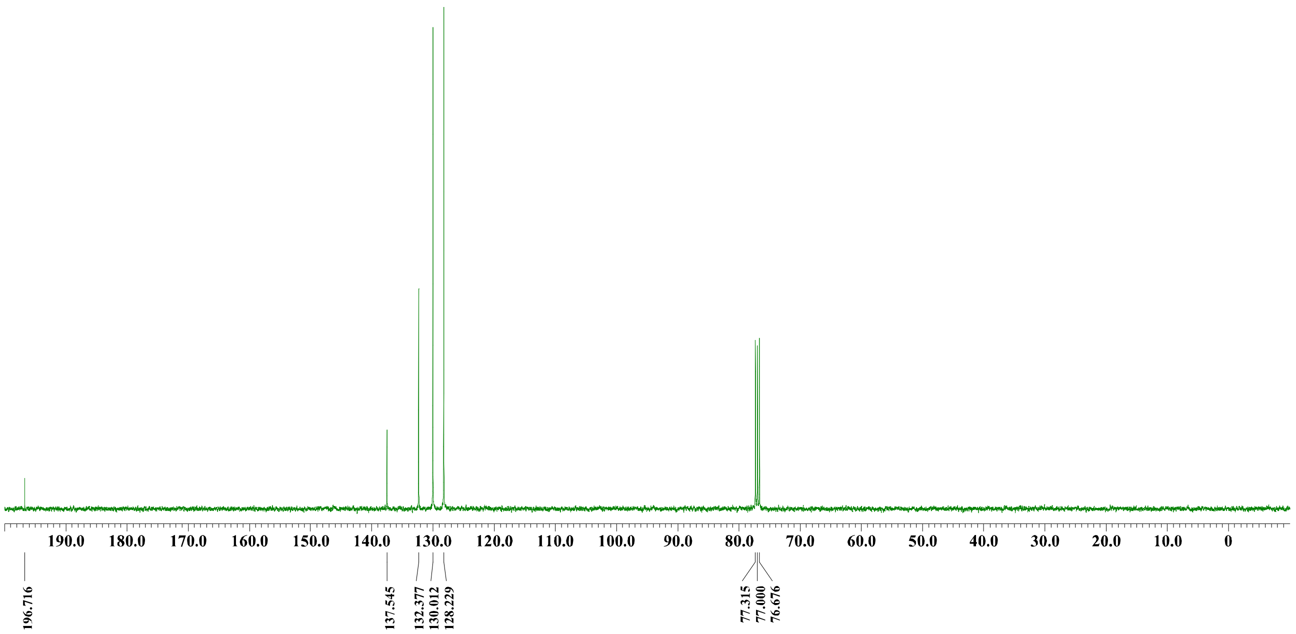

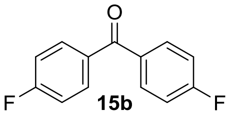

15b

^{1}H NMR (400 MHz, $CDCl_3$) of **15b**

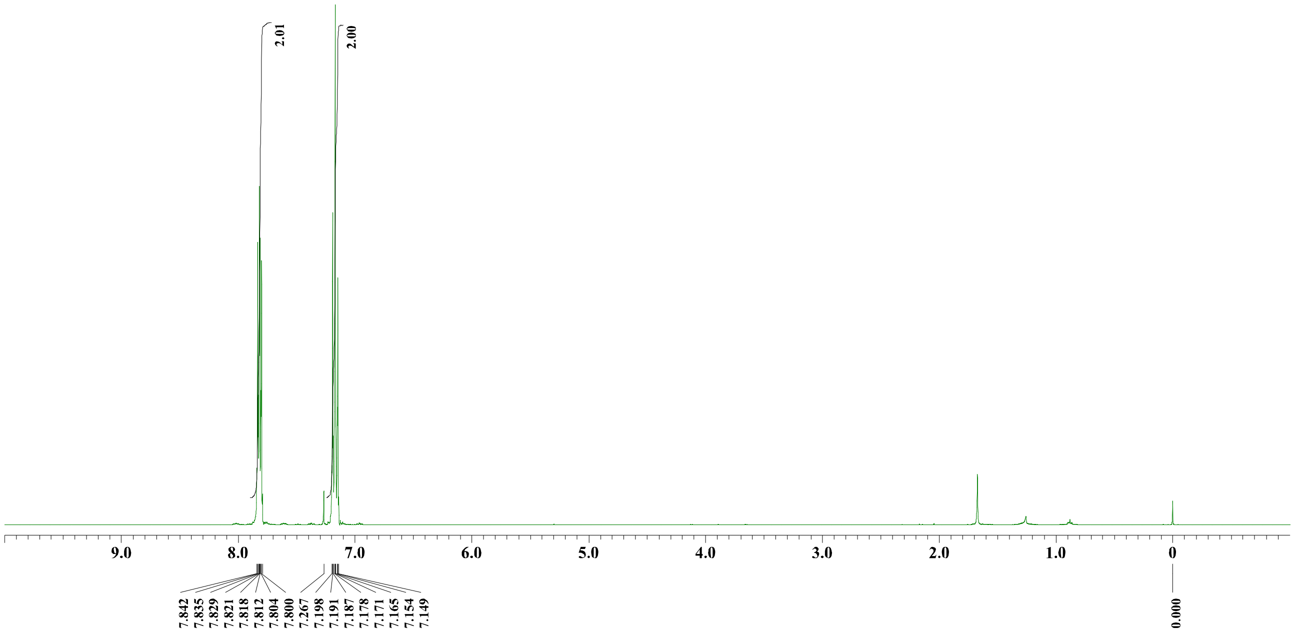

^{13}C NMR (100 MHz, $CDCl_3$) of **15b**

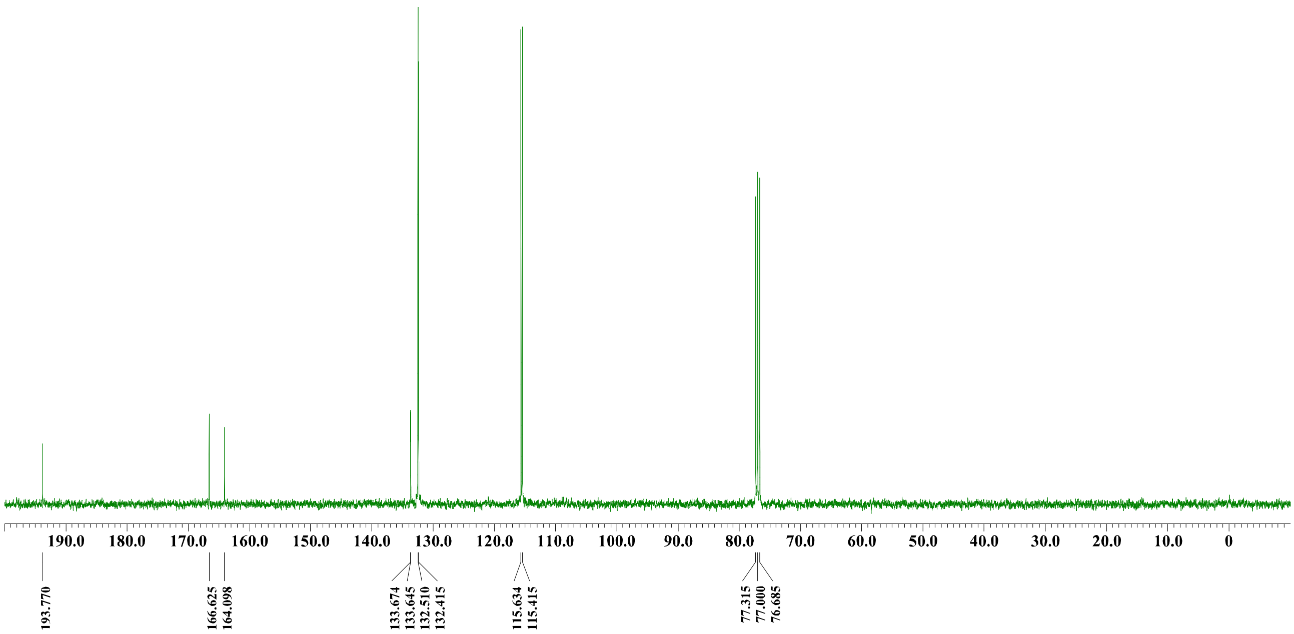

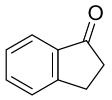

15c

^{1}H NMR (400 MHz, $CDCl_3$) of **15c**

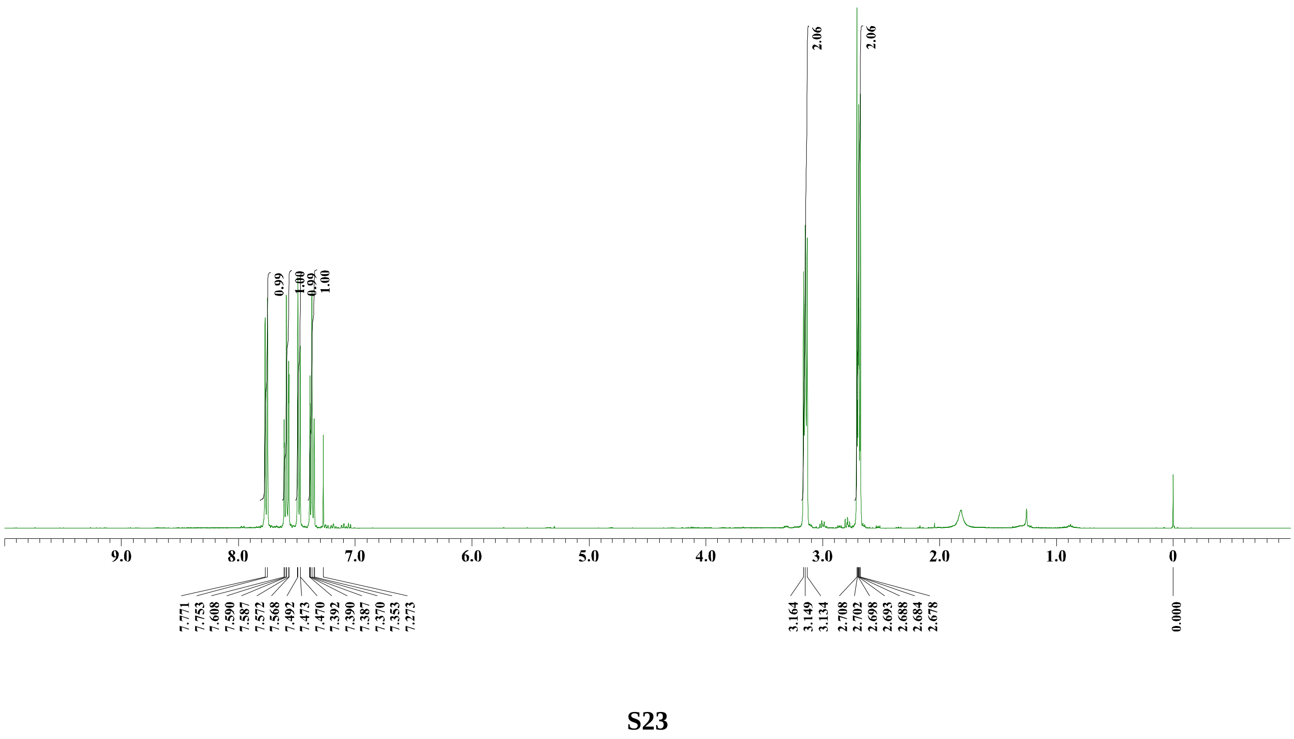

^{13}C NMR (100 MHz, $CDCl_3$) of **15c**

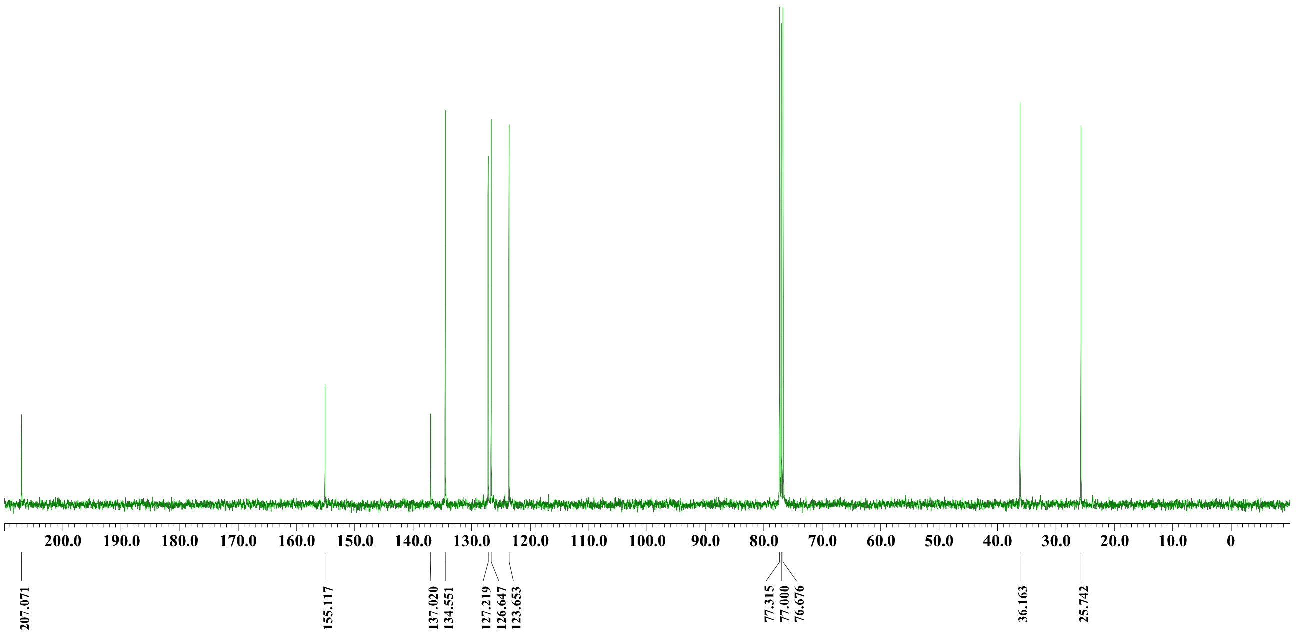

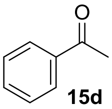

15d

^{1}H NMR (500 MHz, $CDCl_3$) of **15d**

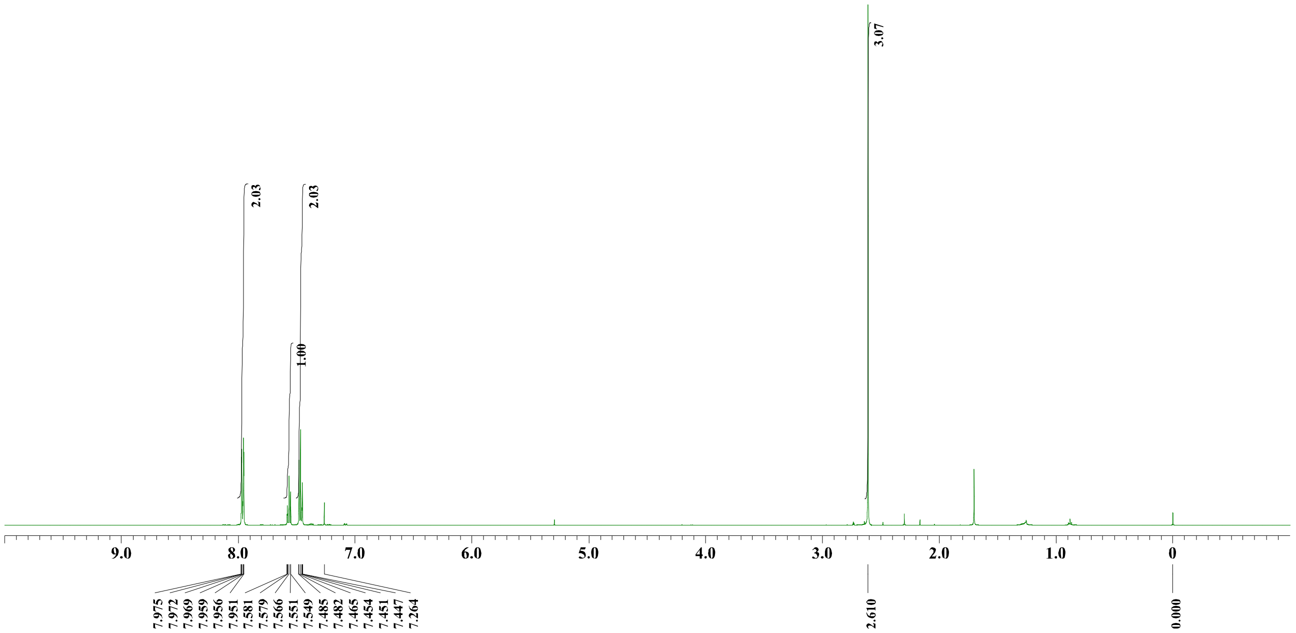

^{13}C NMR (126 MHz, $CDCl_3$) of **15d**

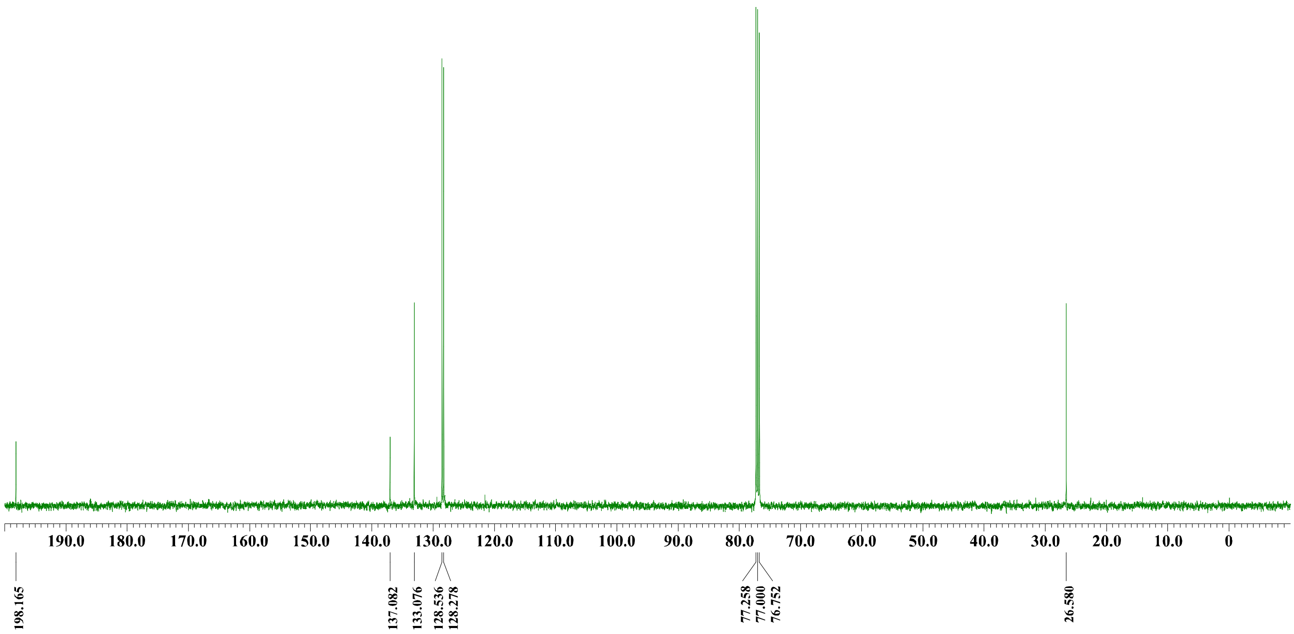

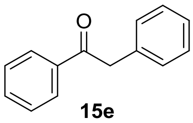

15e

^{1}H NMR (500 MHz, $CDCl_3$) of **15e**

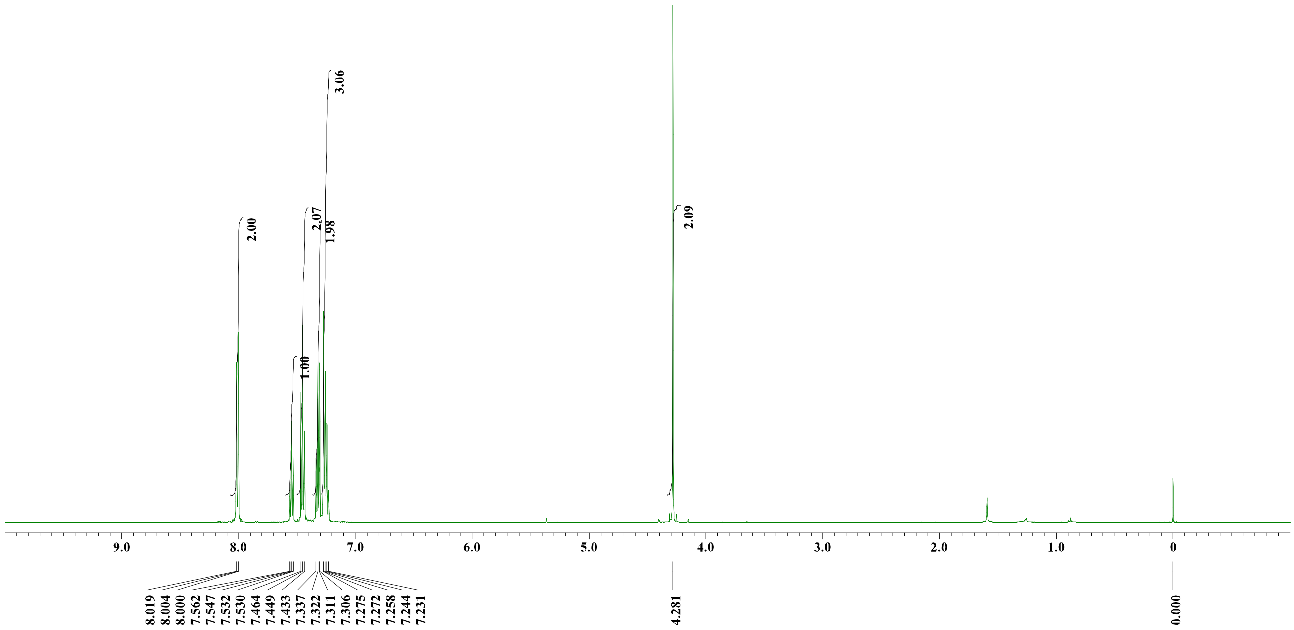

^{13}C NMR (126 MHz, $CDCl_3$) of **15e**

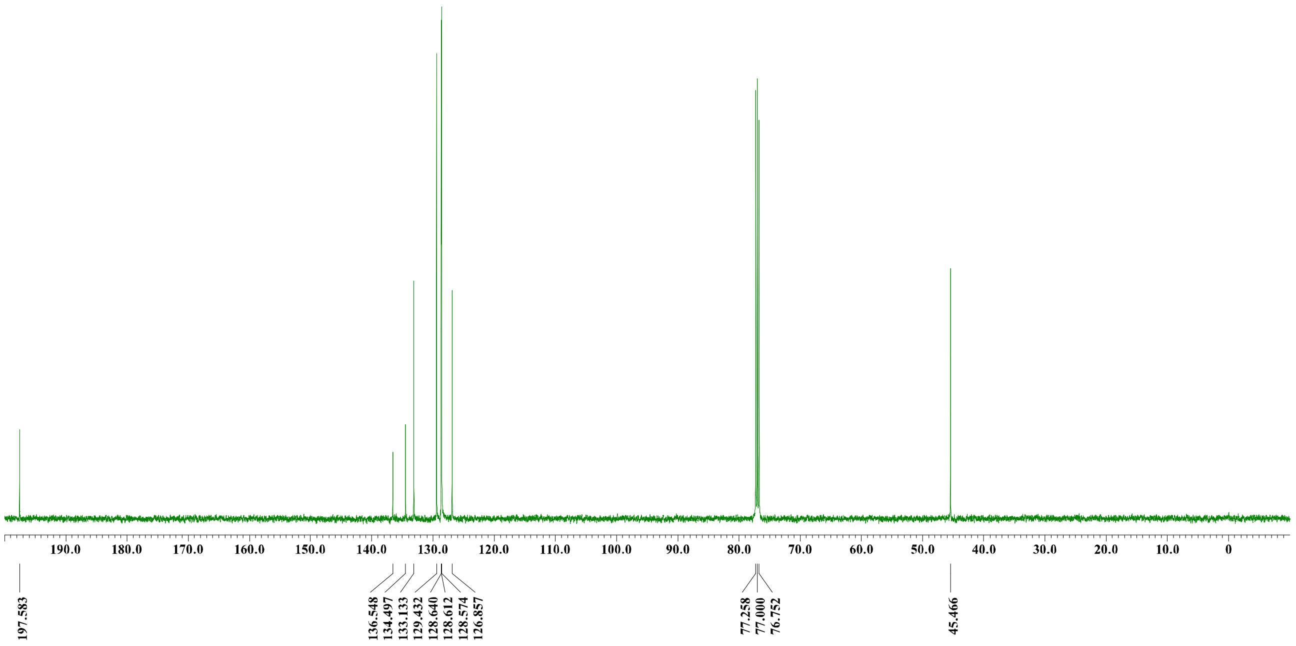

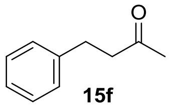

15f

^{1}H NMR (500 MHz, $CDCl_3$) of **15f**

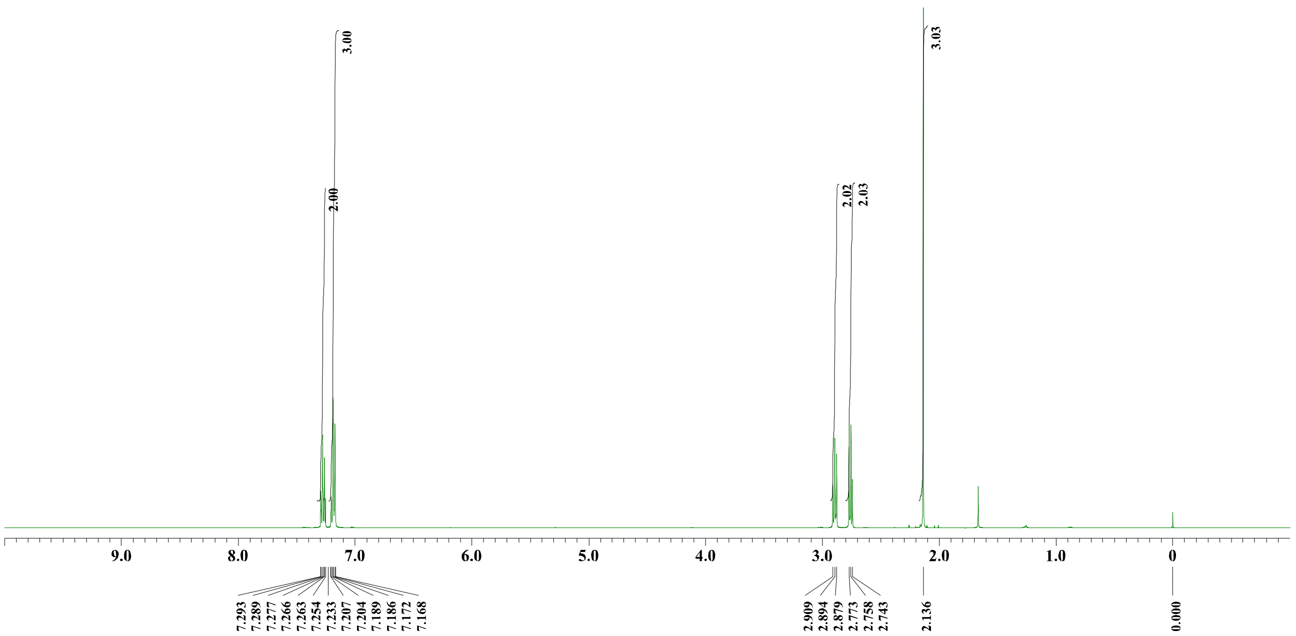

^{13}C NMR (126 MHz, $CDCl_3$) of **15f**

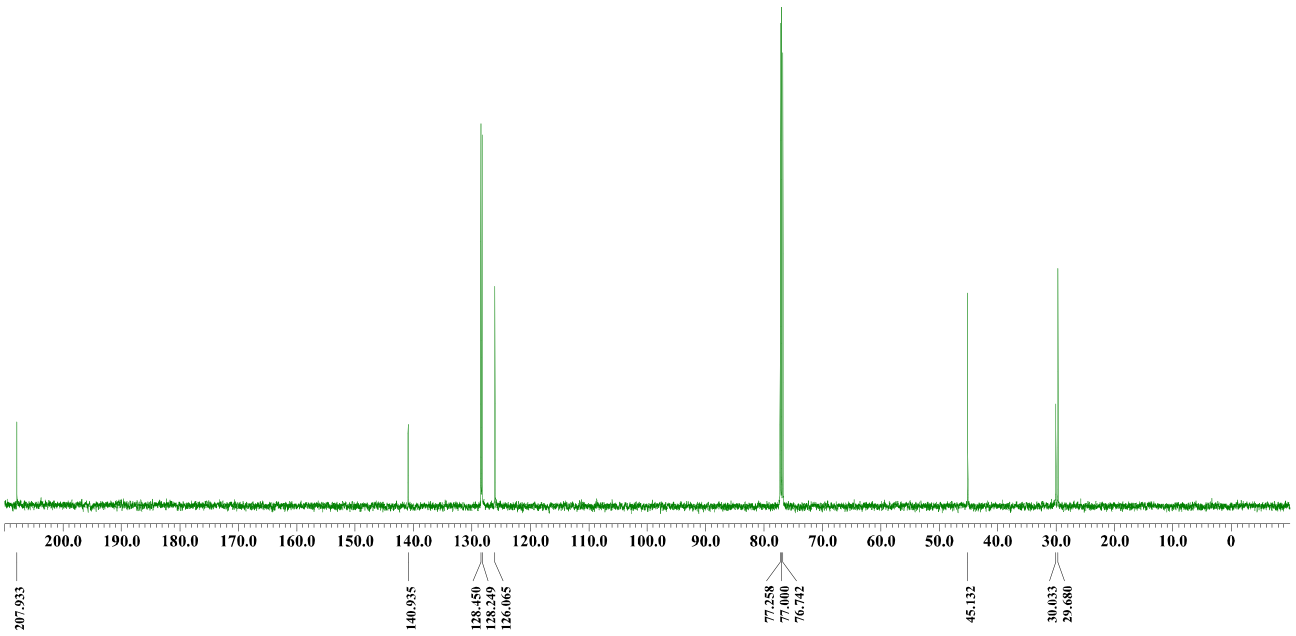

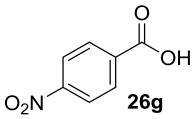

26g

^{1}H NMR (400 MHz, $CDCl_3$/DMSO-d_6 = 4/1) of **26g**

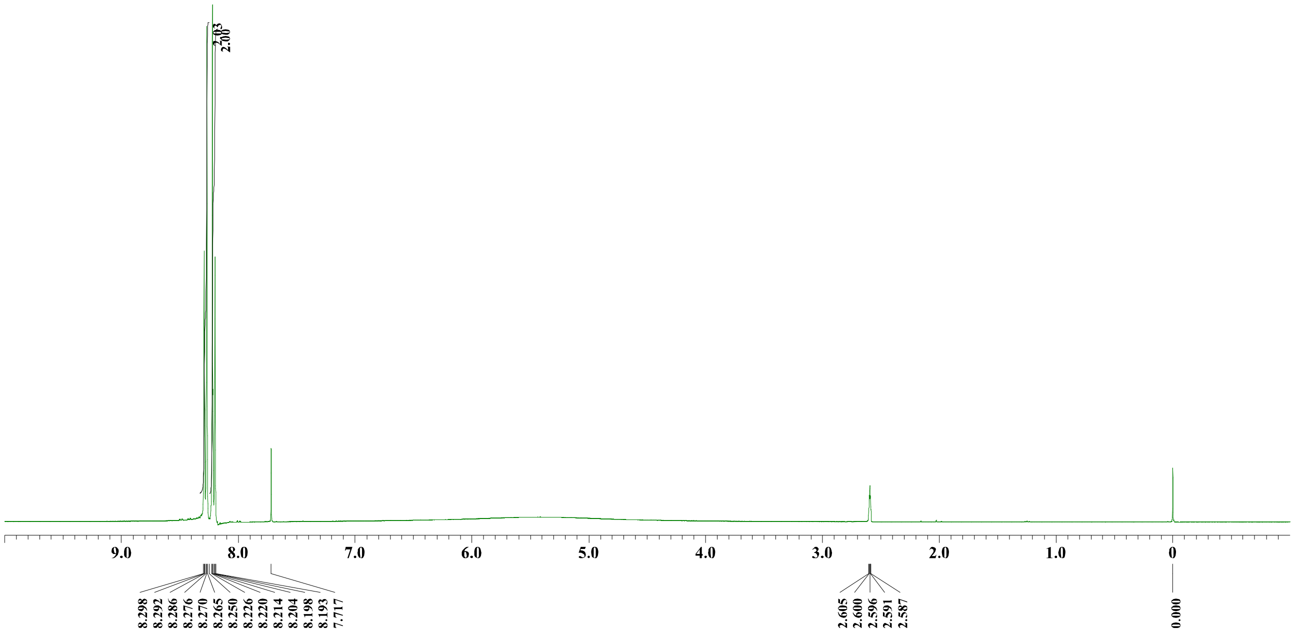

^{13}C NMR (100 MHz, $CDCl_3$/DMSO-d_6 = 4/1) of **26g**

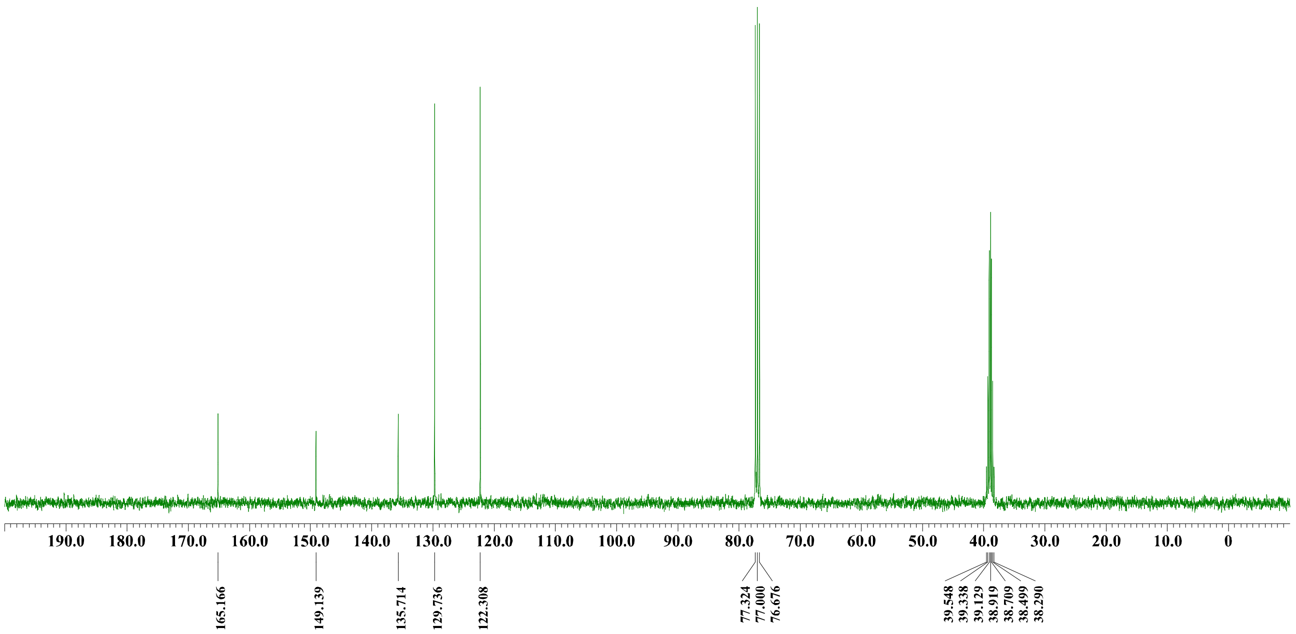

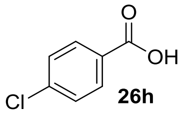

26h

^{1}H NMR (400 MHz, $CDCl_3$/DMSO-d_6 = 4/1) of **26h**

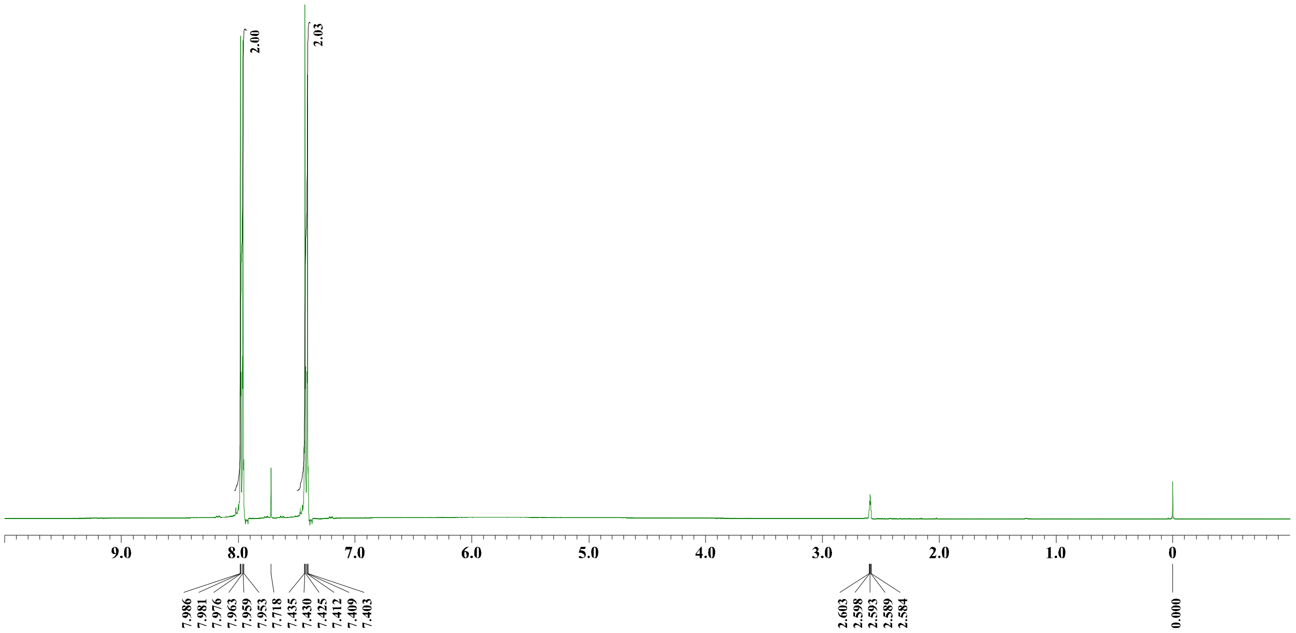

^{13}C NMR (100 MHz, $CDCl_3$/DMSO-d_6 = 4/1) of **26h**

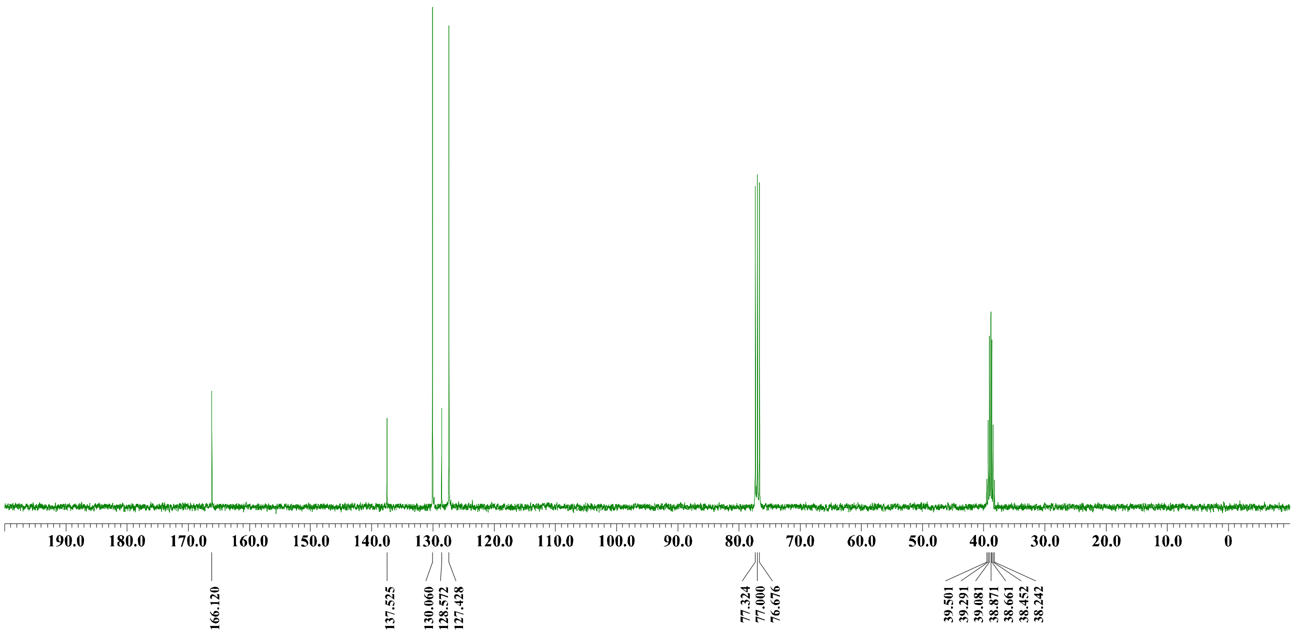

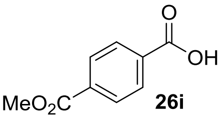

26i

^{1}H NMR (400 MHz, $CDCl_3$/DMSO-d_6 = 4/1) of **26i**

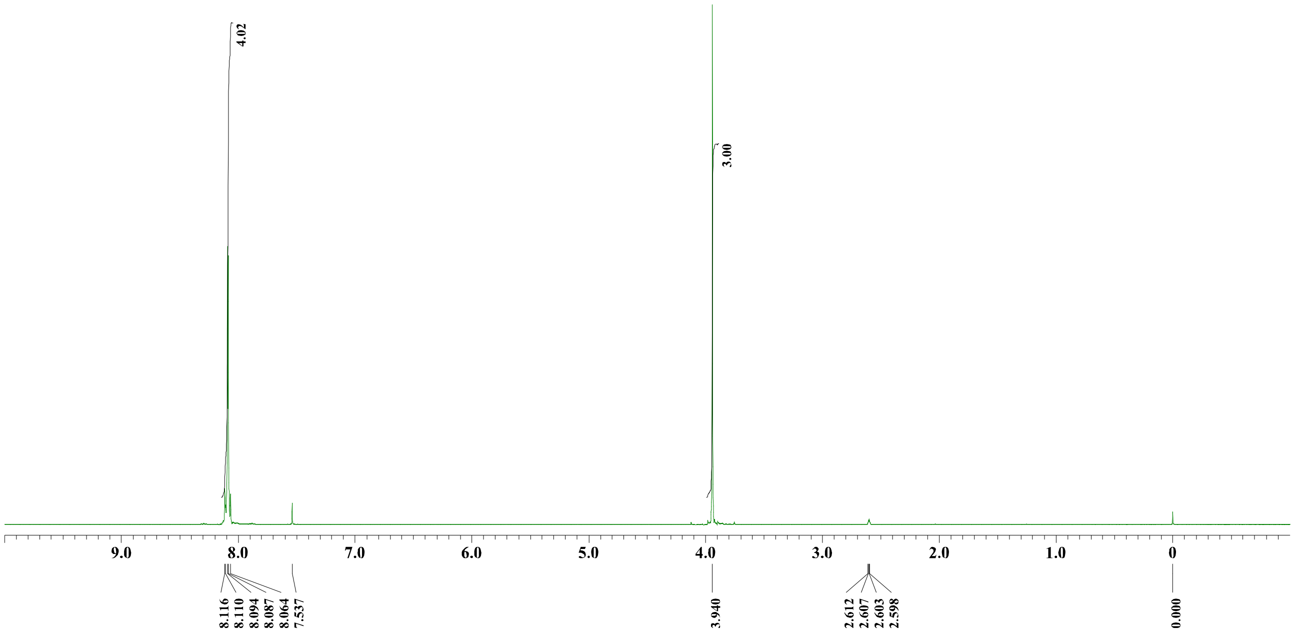

^{13}C NMR (100 MHz, $CDCl_3$/DMSO-d_6 = 4/1) of **26i**

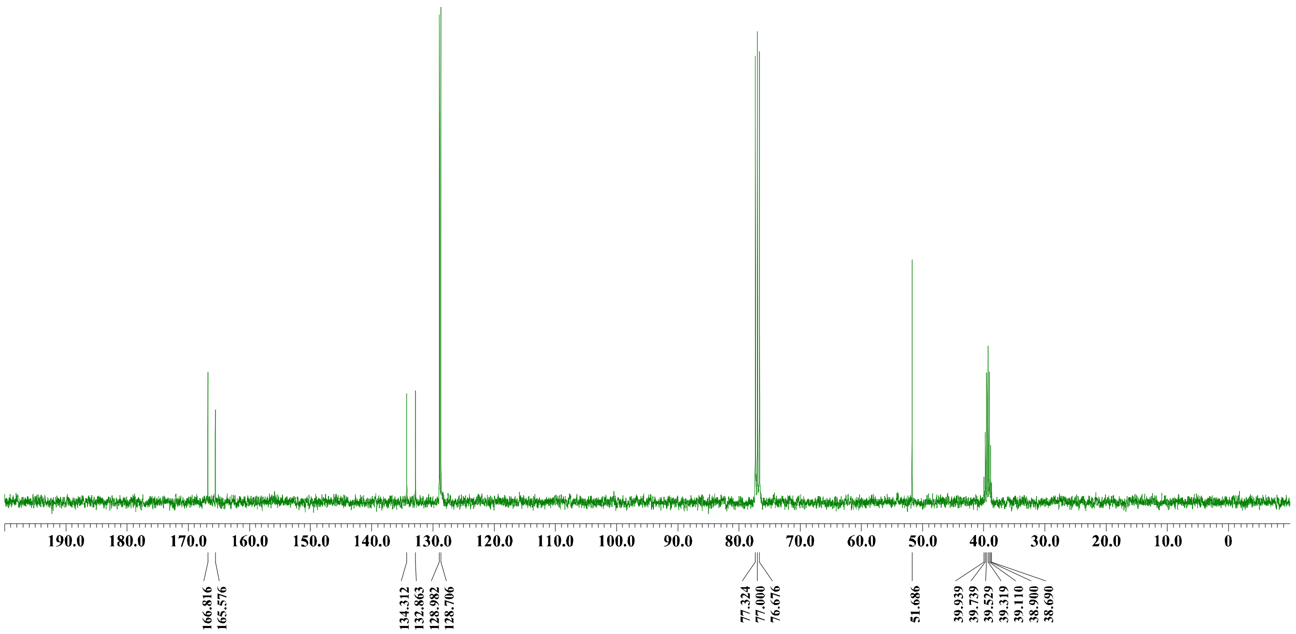

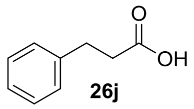

26j

^{1}H NMR (500 MHz, $CDCl_3$) of **26j**

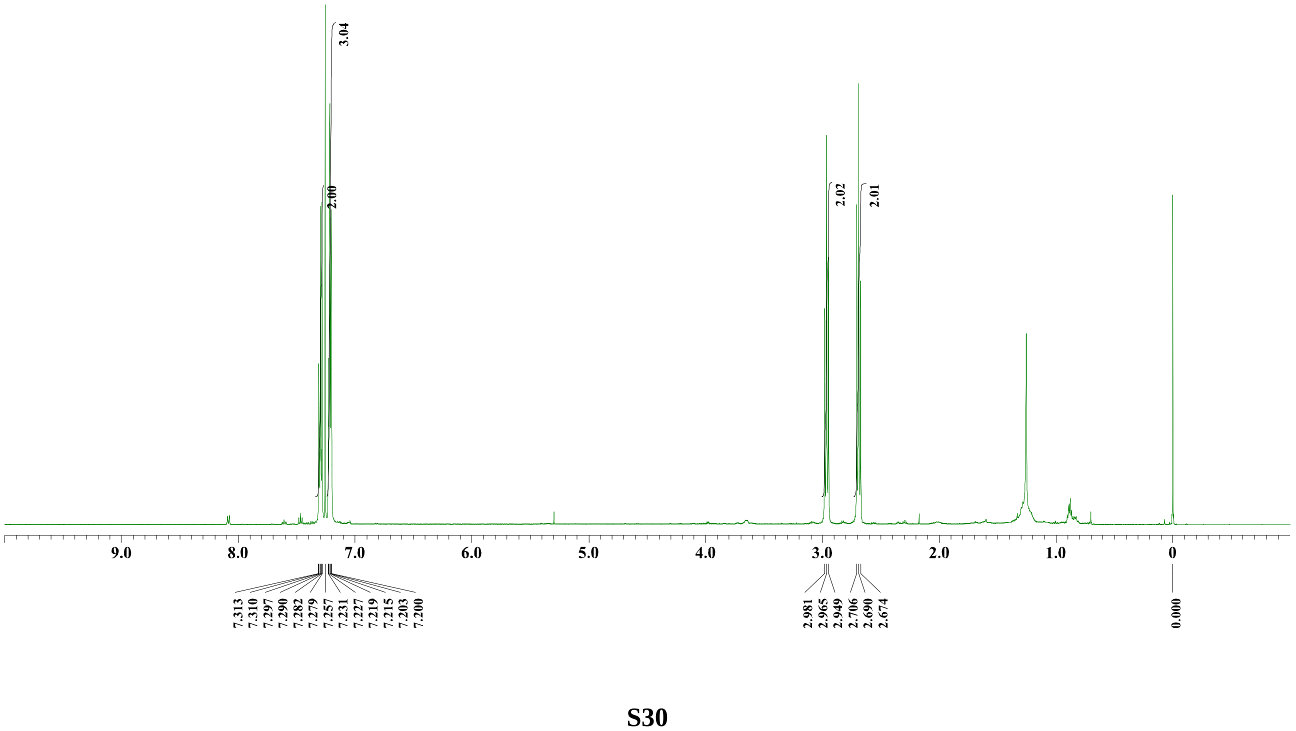

^{13}C NMR (126 MHz, $CDCl_3$) of **26j**

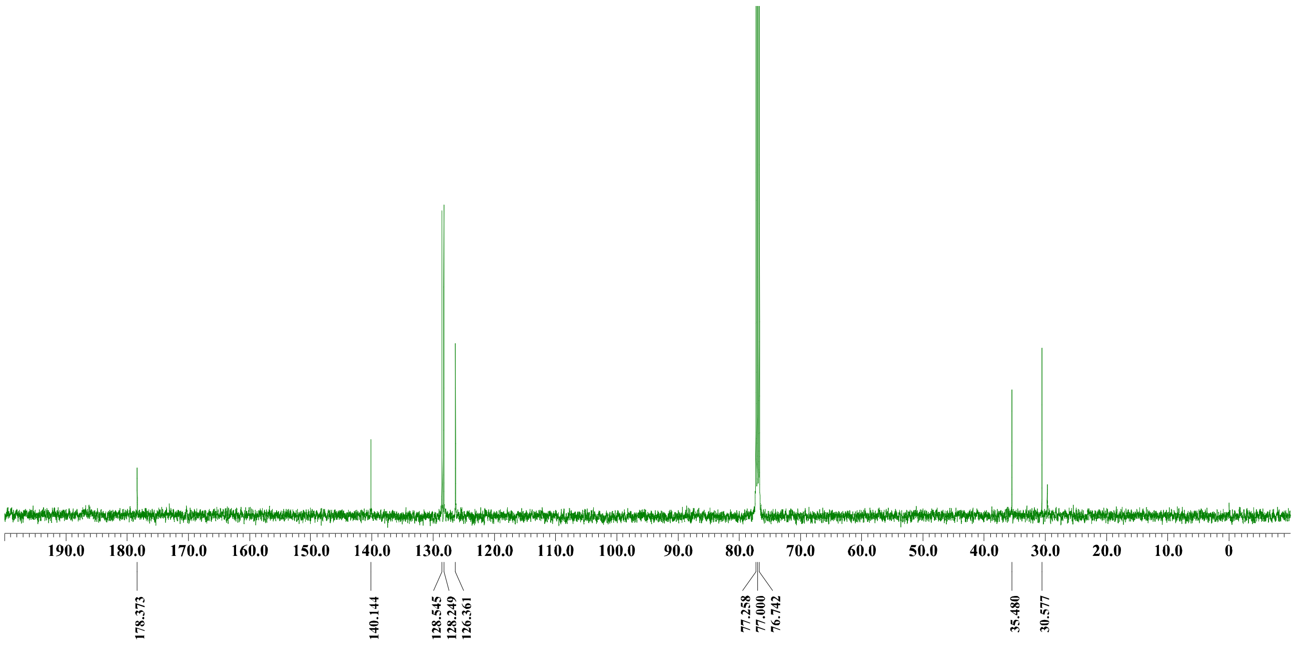

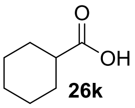

26k

^{1}H NMR (500 MHz, $CDCl_3$) of **26k**

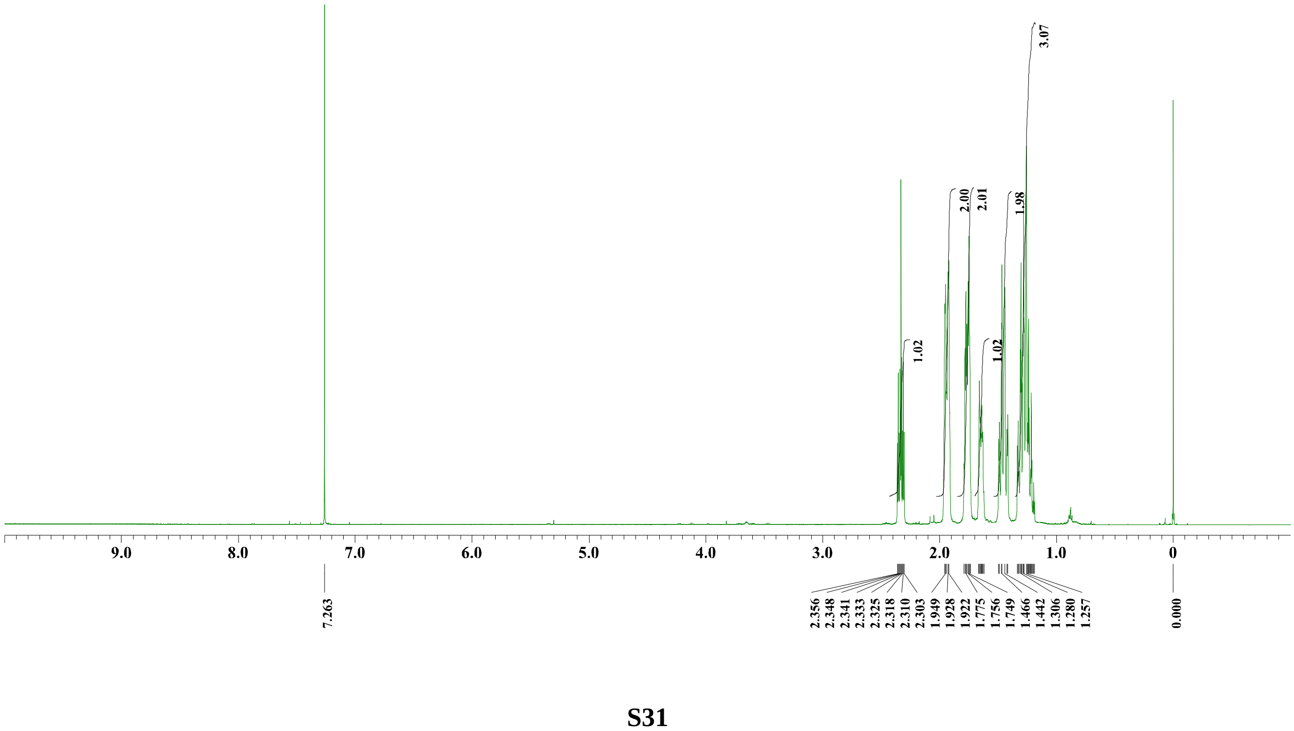

^{13}C NMR (126 MHz, $CDCl_3$) of **26k**

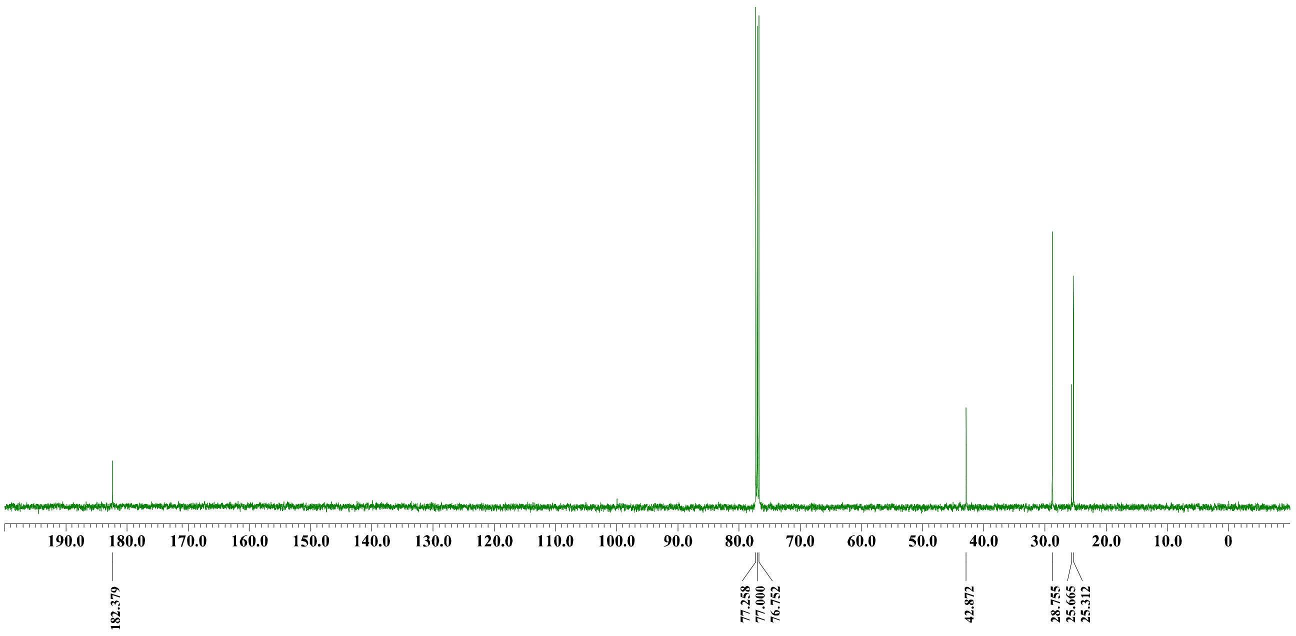

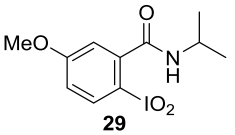

29

^{1}H NMR (400 MHz, DMSO-d_6) of **29**

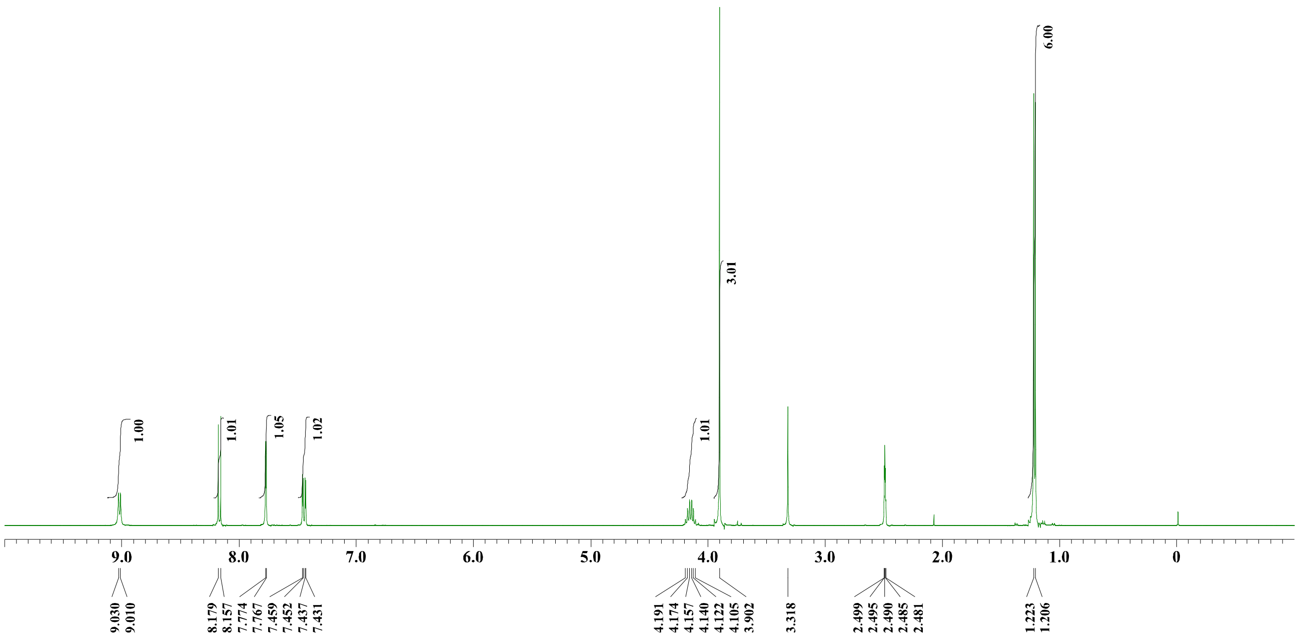

^{13}C NMR (126 MHz, DMSO-d_6) of **29**

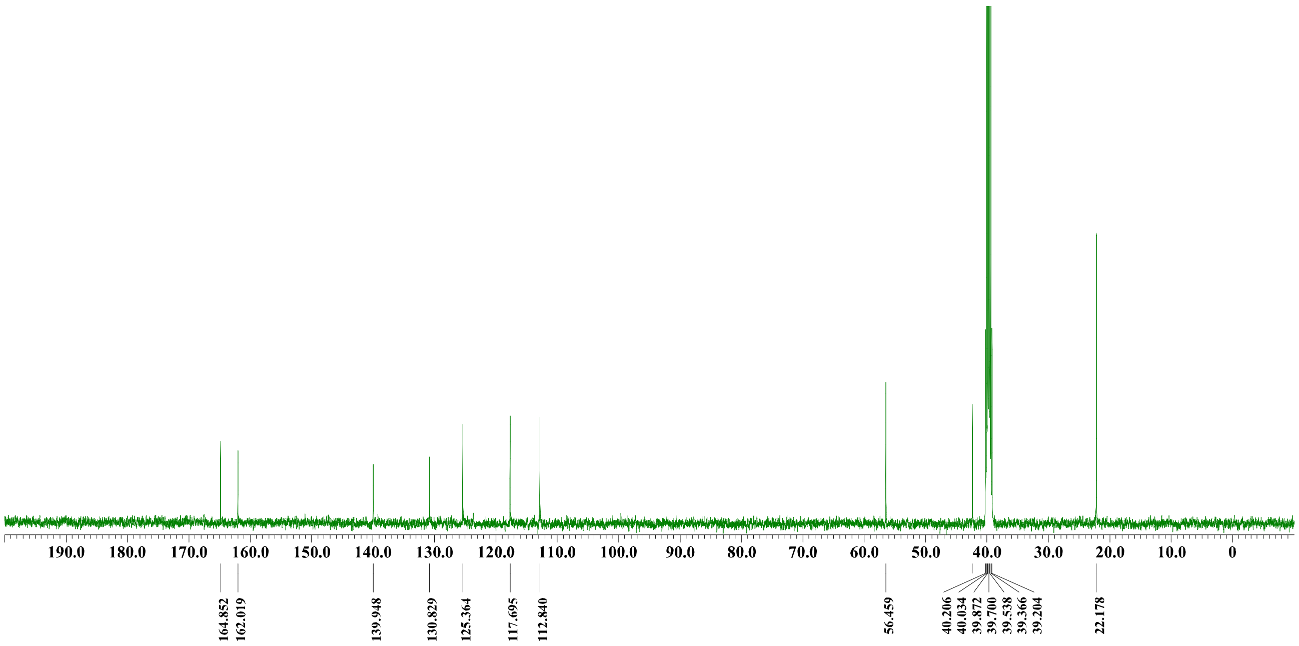

^{1}H NMR (400 MHz, CD_3CN/D_2O = 4) of **29** [as a mixture with $KHSO_4$ and Bu_4NHSO_4 (▲)]

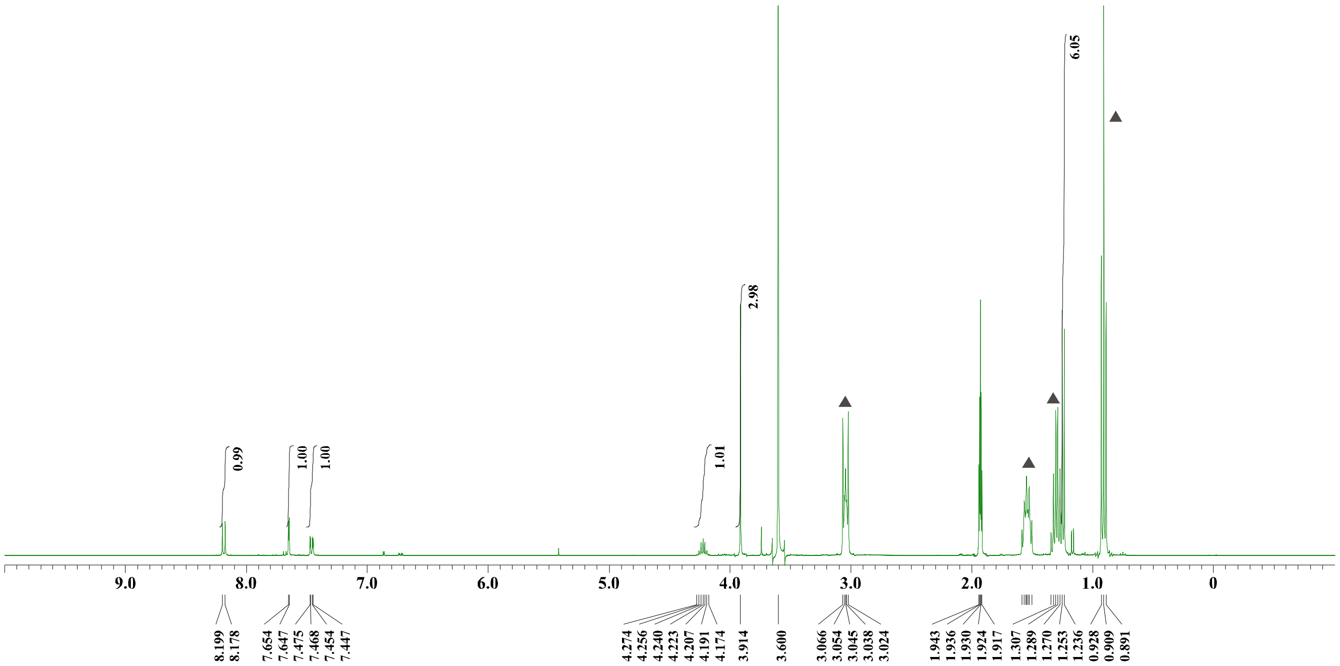

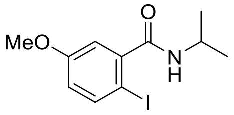

17

^{1}H NMR (400 MHz, CD_3CN/D_2O = 4) of **17** [as a mixture with $KHSO_4$ and Bu_4NHSO_4 (▲)]

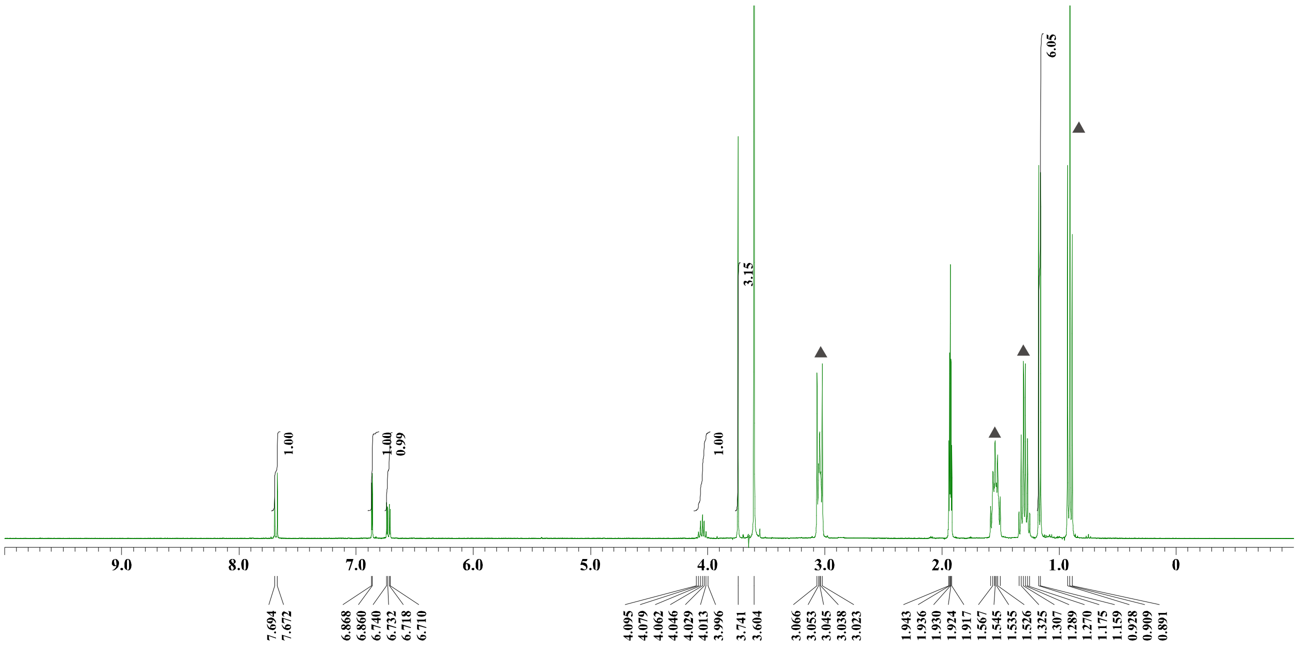

^{13}C NMR (100 MHz, CD_3CN/D_2O = 4) of **17** [as a mixture with $KHSO_4$ and Bu_4NHSO_4 (▲)]

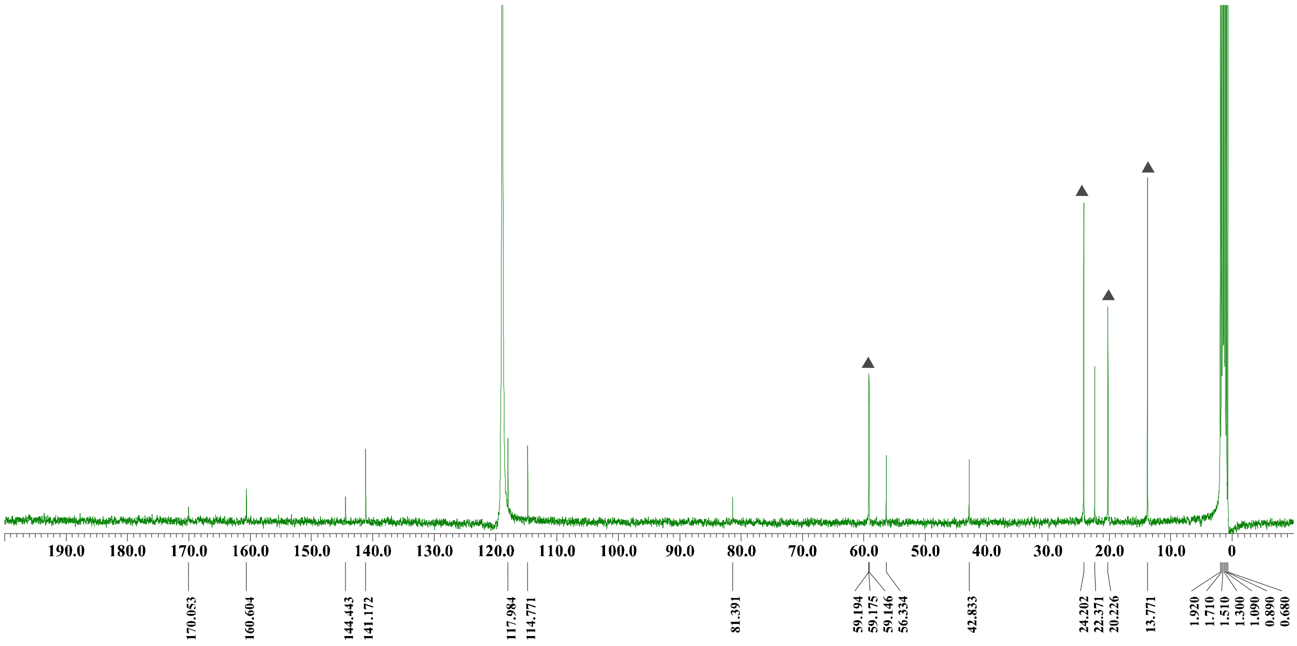

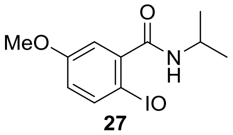

27

^{1}H NMR (400 MHz, CD_3CN/D_2O = 4) of **27** [as a mixture with **17**, $KHSO_4$, Bu_4NHSO_4 (▲), *i*-PrOH, and acetone]

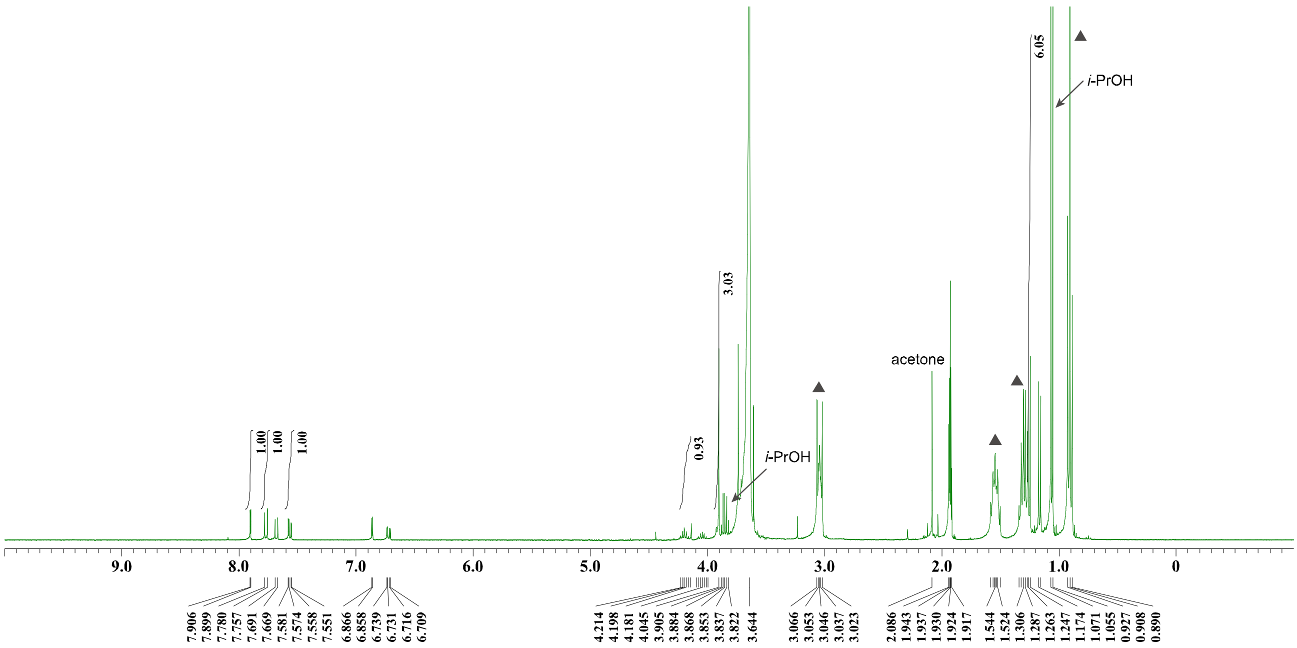

^{13}C NMR (100 MHz, CD_3CN/D_2O = 4) of **27** [as a mixture with **17**, $KHSO_4$, Bu_4NHSO_4 (▲), *i*-PrOH, and acetone]

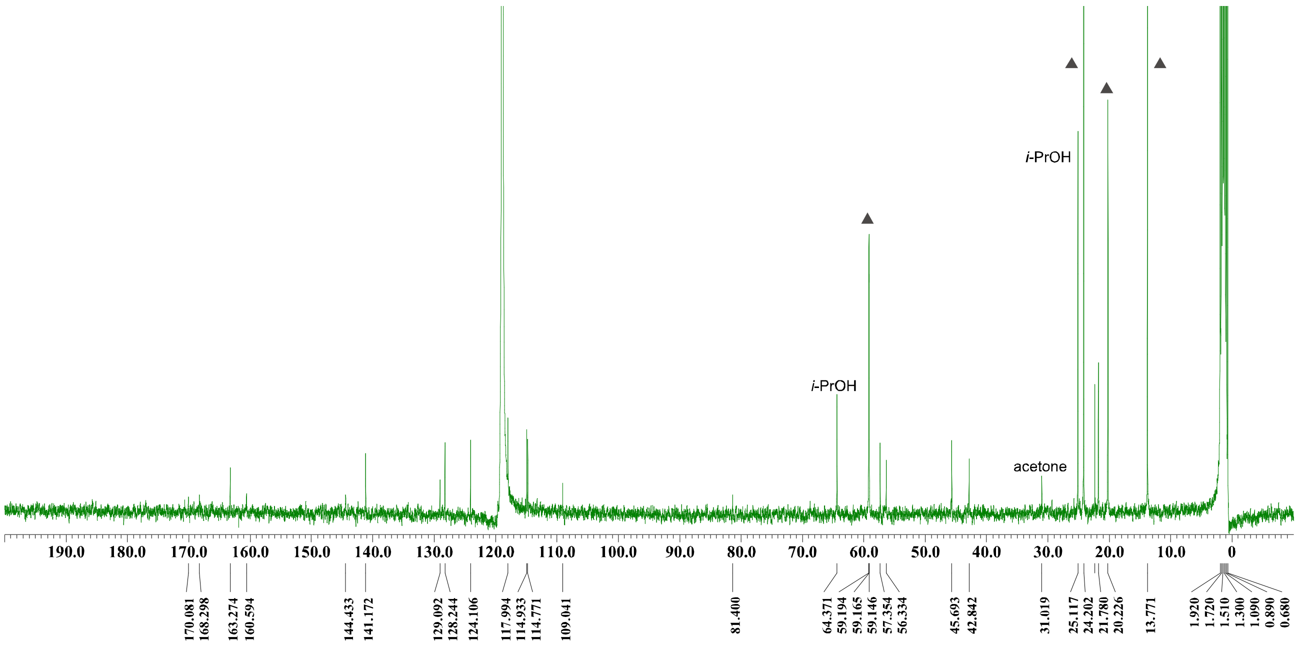

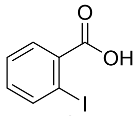

3

^{1}H NMR (400 MHz, CD_3CN/D_2O = 4) of **3** [as a mixture with $KHSO_4$ and Bu_4NHSO_4 (▲)]

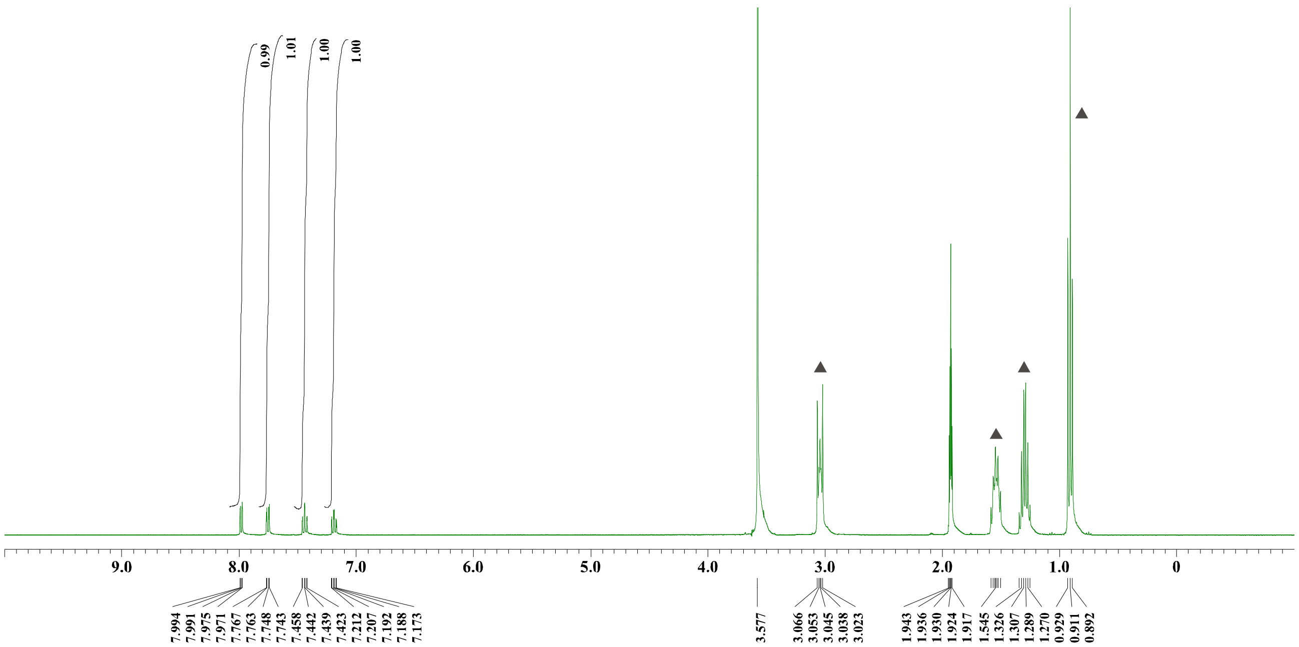

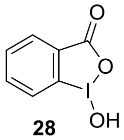

28

^{1}H NMR (400 MHz, CD_3CN/D_2O = 4) of **28** [as a mixture with $KHSO_4$ and Bu_4NHSO_4 (▲)]

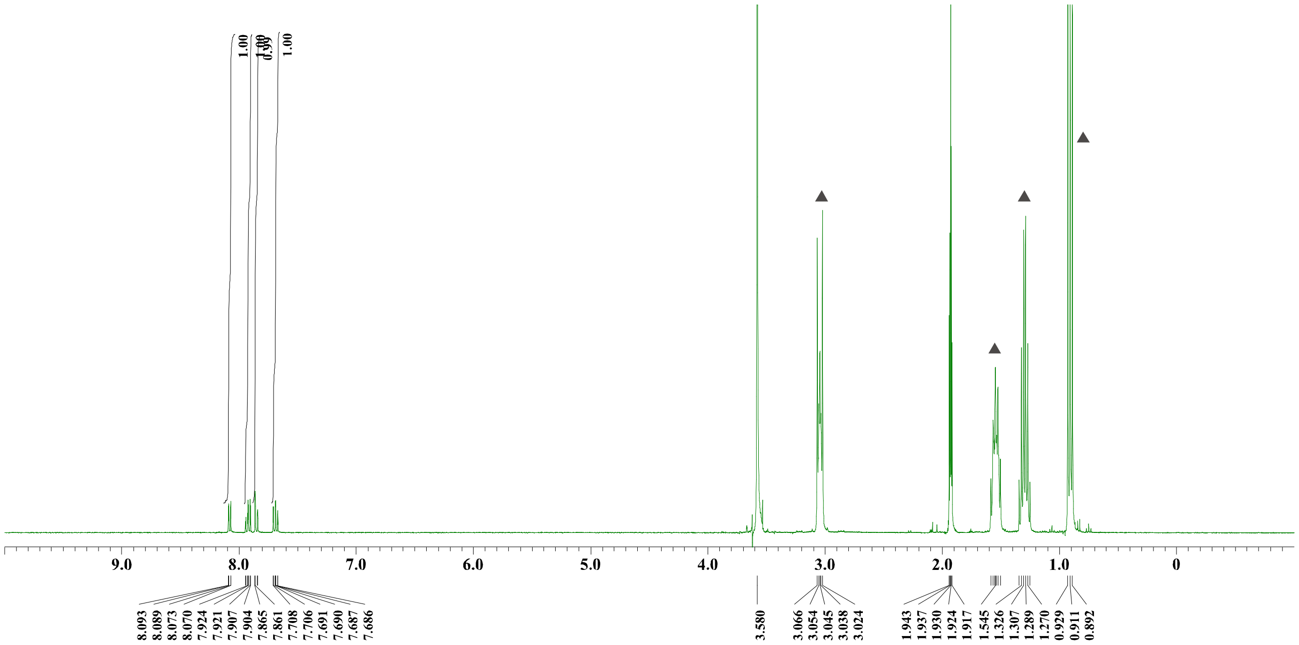

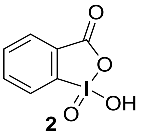

2

^{1}H NMR (400 MHz, CD_3CN/D_2O = 4) of **2** [as a mixture with $KHSO_4$, Bu_4NHSO_4 (▲), **3**, and **28**]

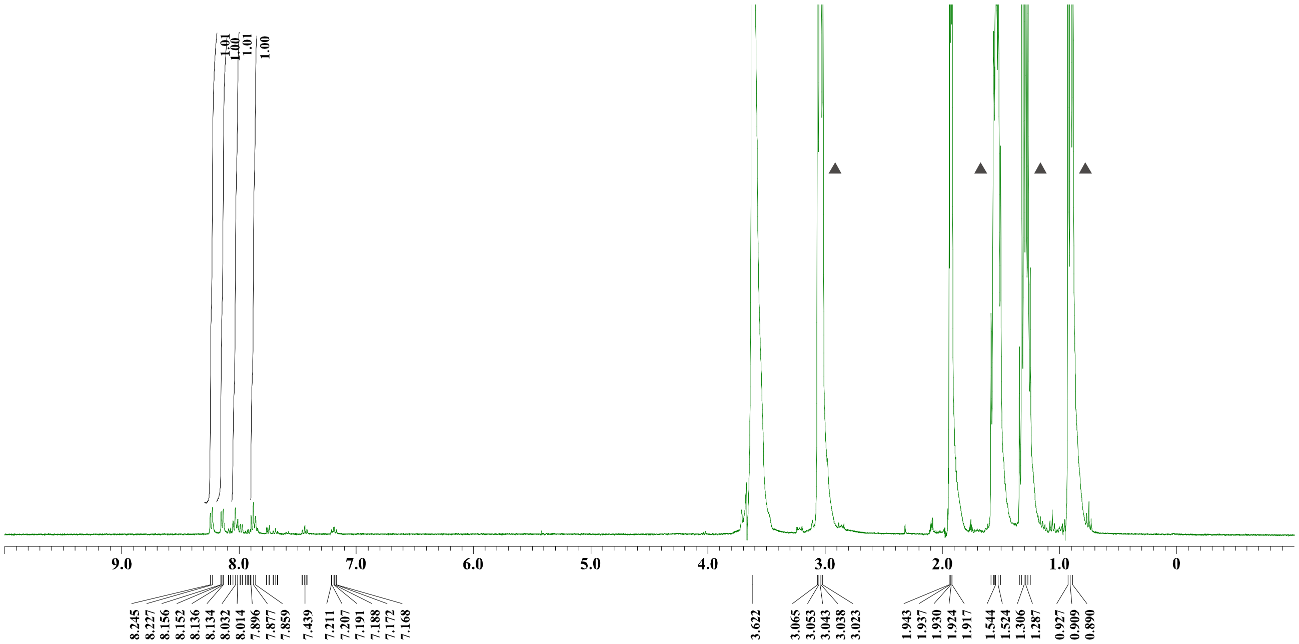

4. References

1. Furuyama, T.; Yonehara, M.; Arimoto, S.; Kobayashi, M.; Matsumoto, Y.; Uchiyama, M. *Chem. Eur. J.* **2008**, *14*, 10348–10356.
2. Patterson, S.; Lorenz, C.; Slawin, A. M. Z.; Westwood, N. J. *QSAR Comb. Sci.* **2004**, *23*, 883–890.
3. Mei, T.-S.; Giri, R.; Maugel, N.; Yu, J.-Q. *Angew. Chem. Int. Ed.* **2008**, *47*, 5215–5219.
4. Ahmed, S.; Hettwer, S.; Vrijbloed, J. W.; Farina, C.; Allgeier, H., PCT Int. Appl. WO2012059442A2, May 10, 2012.
5. Monnereau, C.; Blart, E.; Montembault, V.; Fontaine, L.; Odobel, F. *Tetrahedron* **2005**, *61*, 10113–10121.
6. Pollard, M. M.; ter Wiel, M. K. J.; van Delden, R. A.; Vicario, J.; Koumura, N.; van den Brom, C. R.; Meetsma, A.; Feringa, B. L. *Chem. Eur. J.* **2008**, *14*, 11610–11622.
7. Yakura, T.; Yamada, A.; Noda, N.; Fujiwara, T.; Nambu, H. *Asian J. Org. Chem.* **2014**, *3*, 421–424.
8. Zhdankin, V. V.; Koposov, A. Y.; Netzel, B. C.; Yashin, N. V.; Rempel, B. P.; Ferguson, M. J.; Tykwinski, R. R. *Angew. Chem. Int. Ed.* **2003**, *42*, 2194–2196.
9. Kraszkiewicz, L.; Skulski, L. *Arkivoc* **2003**, *6*, 120–125.
10. Katritzky, A. R.; Duell, B. L.; Gallos, J. K.; Durst, H. D. *Magn. Reson. Chem.* **1989**, *27*, 1007–1011.
11. Frigerio, M.; Santagostino, M.; Sputore, S. *J. Org. Chem.* **1999**, *64*, 4537–4538.
